# Tree selection in urban greening shapes air quality for global cities

**DOI:** 10.1126/sciadv.aee5583

**Published:** 2026-08-19

**Authors:** Xianjun He, Bin Yuan, Yibo Huangfu, Joost de Gouw, Sihang Wang, Suxia Yang, Yuwen Peng, David D. Parrish, Xiaobing Li, Ming Chang, Zhanyou Mo, Kyung-Eun Min, Woohui Nam, Yubin Chen, Xiaoxiao Zhang, Jipeng Qi, Qianqian Xie, Weihua Chen, Alex Guenther, Xuemei Wang, Douglas Worsnop, Thomas Karl, Min Shao

**Affiliations:** ^1^ College of Environment and Climate, Institute for Environmental and Climate Research, Guangdong-Hongkong-Macau Joint Laboratory of Collaborative Innovation for Environmental Quality, Jinan University, 511443 Guangzhou, China.; ^2^ Department of Chemistry and Cooperative Institute for Research in Environmental Sciences, University of Colorado, Boulder, CO 80309, USA.; ^3^ David D. Parrish LLC, 4630 MacArthur Ln, Boulder, CO 80303, USA.; ^4^ Department of Environment and Energy Engineering, Gwangju Institute of Science and Technology, Gwangju, Republic of Korea.; ^5^ Department of Earth System Science, University of California, Irvine, Irvine, CA 92697, USA.; ^6^ Institute for Atmospheric and Earth System Research/Physics, Faculty of Science, University of Helsinki, Helsinki 00014, Finland.; ^7^ Aerodyne Research Inc., Billerica, MA 01821, USA.; ^8^ Department of Atmospheric and Cryospheric Sciences, University of Innsbruck, Innrain 52f, 6020 Innsbruck, Austria.

## Abstract

Mitigating ozone pollution remains a pressing challenge for megacities worldwide. Biogenic volatile organic compounds (VOCs) are proposed as pivotal precursors to urban ozone formation. However, direct observational evidence is scarce and the extent of this effect across cities is unclear. Here, by combining an unprecedented eddy covariance flux dataset from Beijing with modeling analysis, we find that urban vegetation contributes over half of organic reactivity essential for ozone production, predominantly driving ozone exceedances during hot periods. The unexpectedly high biogenic influences are attributed to the abundance of urban trees with strong emission potentials of VOCs within the city. Model extrapolations reveal that similar or even greater challenges exist for other cities across Asia and Oceania, where urban greening strategies often favor highly emitting trees. Our findings underscore the critical need for selective urban planning in greening programs to counteract a warming climate.

## INTRODUCTION

Air quality management continues to be a central challenge for urban areas across the globe (*1*), with ground-level ozone pollution posing a particularly formidable and unresolved problem (*2*, *3*). Although substantial reductions in emissions have led to decreased ozone levels in Europe and North America over recent decades, noncompliance with WHO recommendations remains a persistent issue for numerous urban areas (*4*). Meanwhile, ozone concentrations are rising rapidly across many Asian metropolises (e.g., China and Korea) (*4*, *5*). Urban ozone is formed from photochemistry of volatile organic compounds (VOCs) and nitrogen oxides (NOx) through complex chemical reactions, resulting in a nonlinear response of ozone to its precursors (*6*, *7*). The reliability of ozone concentration predictions from chemical transport models is often limited by notable biases, particularly for daily peaks (*8*–*10*). These inaccuracies can substantially undermine the development of effective control strategies.

Numerous emission sources of VOCs in urban regions exist, emitting a wide variety of VOC species with diverse efficiencies in forming ozone (*11*, *12*). Key emission sources include vehicles, volatile chemical products (VCPs), and urban vegetation. VOC emission-control measures in urban regions have changed relative importance of different sources (*13*, *14*). For instance, although vehicular emissions have decreased markedly, emissions from VCPs remained relatively stable, yet their relative contributions to overall VOC emissions have increased in many US and European cities (*13*, *15*). In addition to anthropogenic sources, many studies, mainly relying on the MEGAN model (*16*), indicate that emissions from urban vegetation may contribute substantially to ozone formation in urban regions (*17*–*21*). However, the characterization of emissions from urban vegetation is still rather inaccurate as urban greening, which refers to the presence and integration of vegetation within built-up urban areas, including street trees, parks, urban forest, and other green infrastructure, and associated plant functional types, is not well represented through land cover data and/or tree species information for biogenic emissions models (*16*, *22*, *23*). A recent work showed that flux measurements of VOCs provide a direct approach to determining the importance of biogenic emissions, reporting ∼60% of VOC reactivity from urban vegetation in summertime Los Angeles (*24*). However, the applicability of these findings to other cities, particularly more polluted megacities in developing countries, is unclear. Moreover, the underlying drivers of intercity variability and environmental consequences for urban greening on air pollution constitute a critical knowledge gap. This limits the capacity to develop effective strategies for managing air quality impacts arising from urban greening.

Here, we present direct evidence for large contributions of urban greening to peak ozone concentrations in summertime Beijing in China, representative of a Chinese megacity, by integrating comprehensive eddy covariance (EC) flux and concentration measurements with multiscale atmospheric chemistry modeling [both zero-dimensional (0D) and three-dimensional (3D)]. We show that the emissions from urban vegetation in Beijing are high, due to abundant isoprene emitting trees within the city. Our prediction indicates that many other cities hold the same or even greater potential for the burden of air quality management from urban greening.

## RESULTS

### Changing VOC emissions and OH reactivity in Beijing

**Fig. 1.**
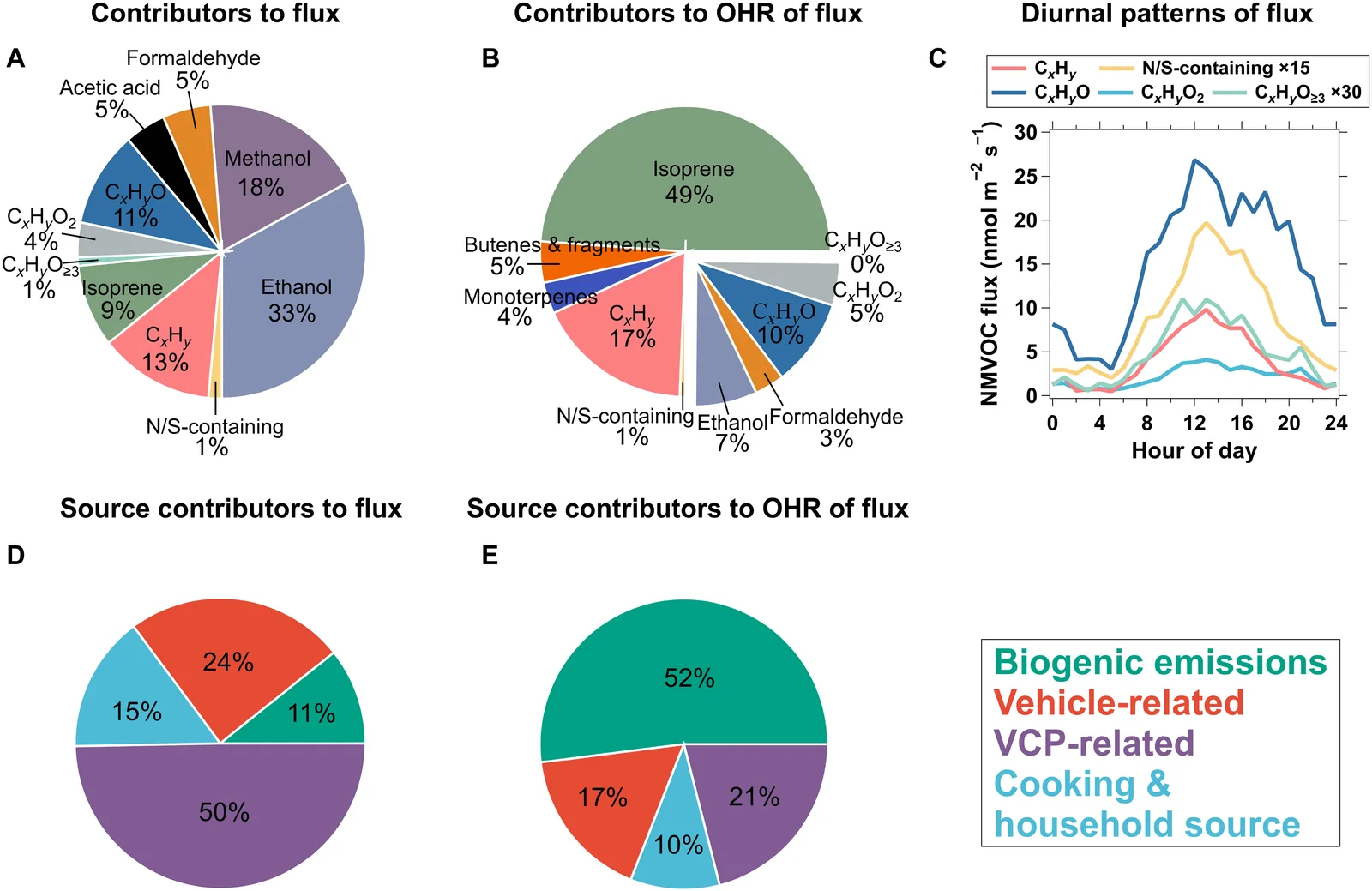
Overview of VOC flux measurements and flux-based source apportionment. Contributions of chemical composition to the (**A**) measured VOC flux and (**B**) measured OHR flux. (**C**) Diurnal patterns of the measured VOC flux. Contributions of the four emission sources from the PMF analysis to (**D**) the estimated total VOC flux and (**E**) the OHR of the estimated total VOC flux after adding the unmeasured species. The top five contributors of VOC species are identified in (A) and (B), and the remaining species were categorized by number of oxygens in the molecules as C*_x_*H*_y_*, C*_x_*H*_y_*O, C*_x_*H*_y_*O_2_, C*_x_*H*_y_*O_≥3_, and N/S-containing species. The VCP-related emission source combines the two resolved factors (VCP-related: OVOCs-rich and VCP-related: aromatics-rich) in the five-factor PMF solution.

VOC flux measurements were conducted in urban Beijing from May to July 2021, using the EC technique with a proton transfer reaction time-of-flight (PTR-TOF) mass spectrometer. This measurement period captures the transition from spring to summer, encompassing late spring (May) and early to mid-summer (June to July) based on meteorological definitions, with ambient temperatures ranging from 14° to 36°C. We found mixing ratios for 766 VOC species above the detection limits, of which 199 VOC species had a detectable exchange flux based on the workflow of flux data processing (fig. S1). Our measurements reveal substantially greater daytime emissions of VOCs, with relatively minor emissions at night (Fig. 1). Almost all species with detectable flux show net emissions. These urban observations contrast with flux observations in forests, where a higher fraction of deposition fluxes were detectable (*25*). Among the VOCs with detected flux, ethanol, methanol, isoprene, formaldehyde, and acetic acid are the highest in Beijing. We also quantify the hydroxyl radical reactivity (OHR) of VOC flux (termed as OHR_flux_ hereafter) as the sum of the product of each VOC’s flux and its OH reaction rate constant (detailed description of the calculation method in text S3 of the Supplementary Materials) (*26*). OHR_flux_ provides a direct indicator for the formation potential of secondary pollutants (e.g., ozone) of fresh VOC emissions from both anthropogenic and biogenic sources. However, OHR_flux_ does not account for potential influences from long-range transport, which is concluded as minor in this study (see Materials and Methods). On the basis of this calculation, we find that isoprene is the most important species, contributing 49 ± 5.4% to OHR_flux_ due to both its large flux and high reactivity.

The extensive VOC flux dataset offers the opportunity to directly extract urban emission sources using a positive matrix factorization (PMF) technique (Materials and Methods). Because EC flux is directly associated with emissions, PMF analysis of VOC fluxes establishes a direct link to emission sources, thereby substantially mitigating limitations inherent to source apportionment based on concentration data (*27*). We further estimate the total VOC emission flux by including potential emissions of unmeasured species based on PMF resolved source profiles (see details in the Supplementary Materials) as most alkanes and a few unsaturated (e.g., ethene and acetylene) species are undetected by PTR-TOF (*28*). Our estimated results (figs. S5 to S7) show that 23% (range: 18 to 28%) of the VOC flux was not detected by our measurements.

On the basis of PMF analysis, we show that a large fraction ($50_{-4}^{+3}$%) of VOC flux is associated with VCP-related sources (Fig. 1D), including one with rich oxygenates and the other with abundant aromatics (fig. S4A). The vehicle-related source, possibly a combination of tailpipe exhausts and evaporative emissions, contributes 24 ± 2% of the total VOC flux, followed by cooking and household sources (15 ± 1%) and biogenic emissions (10.7 ± 0.6%) (Fig. 1D). In terms of source contributions to OHR_flux_, biogenic emissions are identified as the most important source of OHR_flux_, accounting for $52_{-5}^{+4}$% (Fig. 1E), whereas the VCP-related source only accounts for 21 ± 2% of the OHR_flux_ (Fig. 1E). This difference is attributed to the distinct chemical compositions of the contributing sources. For instance, the weighted average for OH rate constants (*k*_OH_) estimated from source signatures for the VCP-related source is the lowest (*k*_avg_ = 5.2 × 10^−12^ cm^3^ molecule^−1^ s^−1^), substantially lower than the biogenic source (*k*_avg_ = 6.6 × 10^−11^ cm^3^ molecule^−1^ s^−1^). This is primarily because the weighted average *k*_OH_ for biogenic emissions is dominated by a highly reactive species—isoprene (1.0 × 10^−10^ cm^3^ molecule^−1^ s^−1^) (*29*), which alone accounts for 60.4% of the biogenic emissions and has a much higher *k*_OH_ than most anthropogenic VOCs (table S4). Correspondingly, the contribution of the vehicle-related source to measured OHR_flux_ is slightly lower (17 ± 2%) than the fraction in terms of flux (24 ± 2%) (Fig. 1E). Our PMF findings on VOC flux indicate two essential facts regarding VOC emissions in Beijing: (i) VCP sources surpass traffic emissions as the largest anthropogenic source of VOCs; (ii) biogenic emissions are the major contributor to OH reactivity of VOC emissions.

Similar to other megacities, VOC emissions in Beijing have changed due to various pollution mitigation measures. The changes can be reflected in the long-term trend of VOC concentrations in summer Beijing (fig. S8). Concentrations of aromatic species (good tracers for vehicles) decreased by a factor of 5 to 10 in the past two decades, whereas C3-C4 ketones (tracers for VCPs) declined by a much smaller portion. Consequently, concentration ratios of C3-C4 ketones to benzene increased by a factor of 3 to 4 during this period. In contrast to anthropogenic species, isoprene concentrations in Beijing changed little over this time, although there was considerable year-by-year variability. These results reveal that anthropogenic VOCs in Beijing decreased gradually, with the relative fractions of vehicle-related emissions diminishing and VCP emissions increasing, whereas the relative contribution of biogenic emissions became prominent.

Combining PMF analysis of flux measurements with long-term variations of VOC concentrations, we demonstrate that the substantial reduction of anthropogenic sources in Beijing has changed the pattern of VOC emissions, with only half of the OH reactivity of VOCs in summer stemming from anthropogenic sources at present. Recently, an airborne flux study showed a comparable contribution (∼60%) of biogenic emissions to VOCs reactivity in the summer of Los Angeles (*24*, *30*). Our results indicate that the relative importance of biogenic VOC (BVOC) emissions in Beijing is comparable to that of typical US cities (*13*, *31*), after a marked reduction of previously dominant vehicular emissions in Beijing in recent years. The high fraction of OHR_flux_ contributed by biogenic emissions is unexpected as Beijing, a representative megacity in the developing world, has been regarded as more polluted, with stronger anthropogenic emissions than megacities in the US and Europe, which would be expected to result in a minor importance of biogenic emissions.

### Strong influence of biogenic emissions on ozone formation

**Fig. 2.**
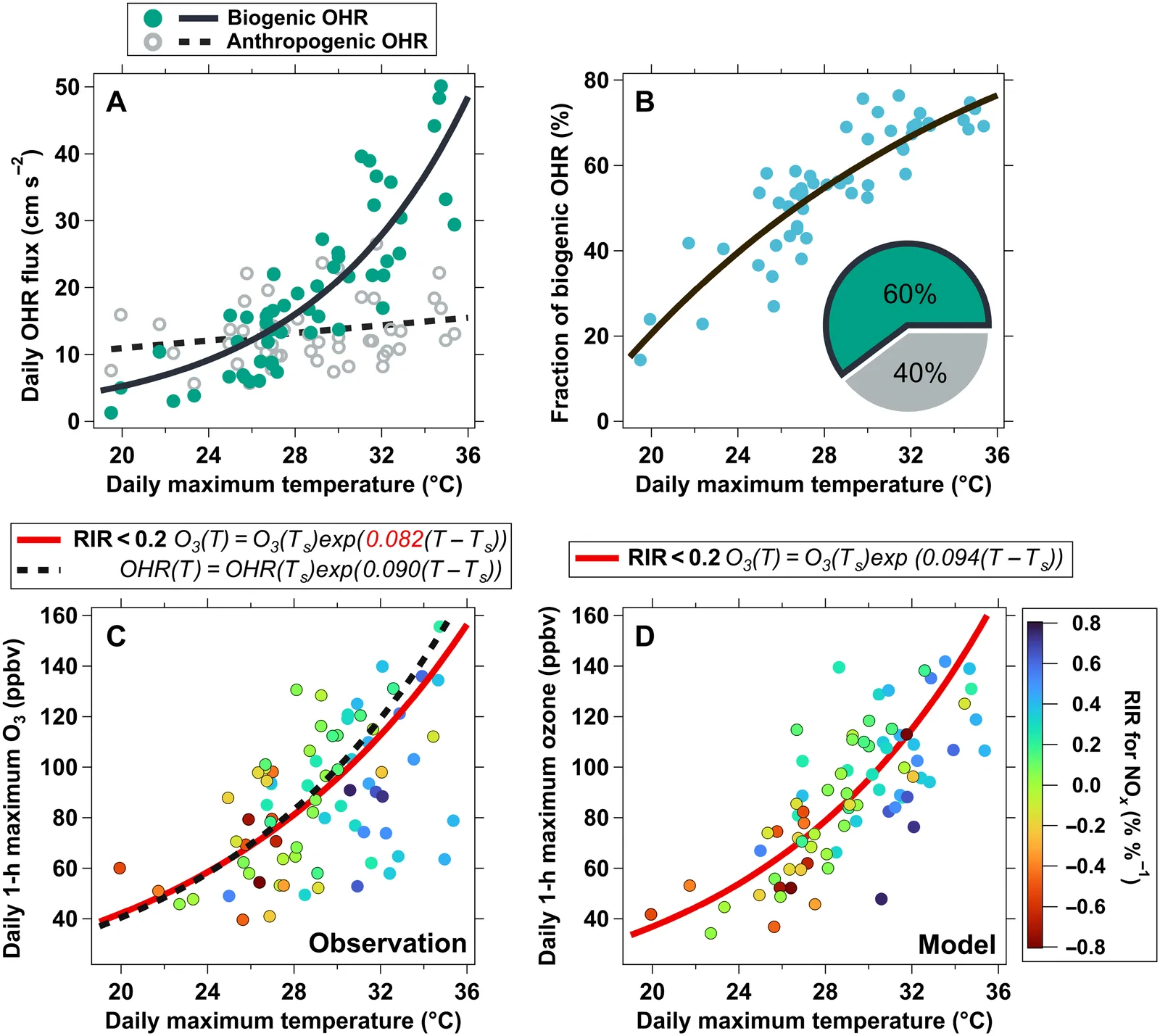
Summary of temperature dependence of OHR_flux_ and ozone. Temperature dependence for (**A**) biogenic OHR_flux_, anthropogenic OHR_flux_, (**B**) fraction of biogenic OHR_flux_, (**C**) observed ozone concentrations, and (**D**) modeled ozone concentrations. (A and B) Derived from measured daytime (08:00 to 18:00) data, with biogenic and anthropogenic emissions resolved using PMF analysis; the anthropogenic component represents the combined contributions from all four anthropogenic factors. The pie chart in (B) represents the average contributions of biogenic emissions (green) and anthropogenic emissions (gray) in the daytime. The dashed line in (A) is the linear fit to anthropogenic OHR_flux_ (*y* = 0.29*x* + 5.2). The black solid line in (A) and colored lines in (C) and (D) are fitted using *Y*(*T*) = *Y*(*T*_s_) × exp[β(*T* – *T*_s_)] from biogenic OHR_flux_ and ozone concentrations, where *Y*(*T*_s_) is the *Y* parameter at *T*_s_ (25°C), β is the temperature response factor, and *T* is the ambient temperature. The black dashed line in (C) represents the temperature response of the total OHR_flux_ as shown in fig. S11C. h, hour.

Extensive research has shown that BVOC emissions are largely dependent on temperature during the daytime (*32*). We observe a strong temperature dependence for typical BVOC (e.g., isoprene) from both concentration and flux datasets (fig. S11, A and B). We also observe a large temperature dependence of OHR_flux_ (fig. S11C). To systematically analyze the source of temperature dependence, we separate the total OHR_flux_ into the anthropogenic and biogenic components based on the PMF analysis. As shown in Fig. 2A, we obtain a profound temperature dependence of OHR_flux_ associated with biogenic emissions but much less for anthropogenic emissions (combined from all anthropogenic factors). The determined exponential factor for the OHR_flux_ associated with biogenic emissions (0.090; Fig. 2C) is comparable to a previous study (*16*). OHR_flux_ from biogenic and anthropogenic sources at 35°C increases by 700% and 40% compared to 20°C, respectively. This leads to a markedly larger fractional contribution of biogenic sources to OHR_flux_ at elevated temperatures (21% at 20°C to 74% at 35°C) than for anthropogenic sources (Fig. 2B). These results indicate that the temperature dependence of OHR_flux_ is primarily driven by biogenic emissions.

Temperature dependence of daily peak ozone concentrations was explored at both the flux site (Fig. 2C) and other monitoring sites in Beijing from the China National Environmental Monitoring Center (CNEMC) network (fig. S12). Previous works showed that the ozone temperature dependence is strongly affected by ozone chemical sensitivity, with stronger dependence for the VOC-limited regime than the NOx-limited regime (*33*, *34*). On the basis of comprehensive observation-constrained box model simulations, we show that ozone formation in Beijing varies drastically between the chemical regimes from day to day (fig. S14). For the investigation of the influence of OHR_flux_ on ozone formation, we intentionally select the periods of ozone formation mainly controlled by VOCs [i.e., relative incremental reactivity (RIR) for NOx < 0.2% %^−1^]. We show that the temperature dependence of ozone for VOC-controlling periods mirrors that of the OHR_flux_ with comparable exponential fitting coefficients (Fig. 2C, 0.082 for the flux tower site and 0.081 for all monitoring sites in Beijing). As the temperature dependence of OHR_flux_ is primarily driven by biogenic emissions, this similarity in temperature dependencies suggests a direct link between OHR_flux_ from urban vegetation and peak ozone formation—an aspect that warrants further consideration in urban air quality management.

Both simulations by the box model (fig. S15) and 3D chemical transport model (fig. S17, Materials and Methods, and Supplementary Materials) reinforce that urban vegetation strongly influences peak ozone formation in Beijing. Our observation-constrained box model indicates that biogenic emissions account for an average of 18.5 ± 8.6 parts per billion by volume (ppbv) for daily peak 1-hour ozone concentrations, which is comparable to the modeling results for Beijing’s built-up area from the chemical transport model (22.2 ± 10.2 ppbv). A further sensitivity simulation from the chemical transport model demonstrates that urban vegetation inside the built-up area contributes 57% of biogenic influences (i.e., 12.8 ± 6.8 ppbv) to peak ozone concentrations (fig. S18). Moreover, this ozone enhancement exhibits strong spatial consistency across the entire built-up area (fig. S17B), which underscores the spatial representativeness of our findings and supports the robustness of the conclusions across different modeling frameworks.

The temperature dependence analysis of ozone also provides critical information for effective ozone mitigation in the future. As shown in Fig. 2 (C and D), ozone formation tends to be more likely within the NOx-limited regime for days with higher temperature because more VOCs are available for photochemistry. Now, ∼1/3 of days with a daily maximum temperature above 32°C are in the NOx-limited regime. If NOx emissions are further reduced in Beijing, especially on hot days, chemical sensitivity is more likely to be NOx limited, which would restrict the ability of emissions from urban vegetation to produce ozone. Thus, emission reduction of NOx during hot days—such as enhanced controls on industrial and vehicular emissions—remains an effective ozone mitigation strategy in Beijing. Climate models predict that the daily maximum temperature in the North China Plain would increase by 0.8° to 3.0°C in 2050 and 2° to 8°C in 2100 due to climate change (*35*), which would inevitably lead to a pronounced increase in the frequency of urban heat waves. The strong rise of temperature would promote BVOC emissions markedly, necessitating more rigorous reduction measures for anthropogenic sources during heat waves, with a particular focus on NOx emissions. Another factor that makes urban vegetation unique from a natural ecosystem is the urban heat island effect. We estimate that, for a typical urban heat island intensity (0.8°C) (*36*) during summer daytime in Beijing (assuming a maximum daily temperature of 32°C), isoprene emissions from urban trees increase by 12%, resulting in a 7.4% increase in OHR_flux_ from biogenic emissions and a 6.8% (or 9.0 ppbv) increase in peak ozone concentrations.

## DISCUSSION

### Tree species composition shapes variability in biogenic emissions across cities

**Fig. 3.**
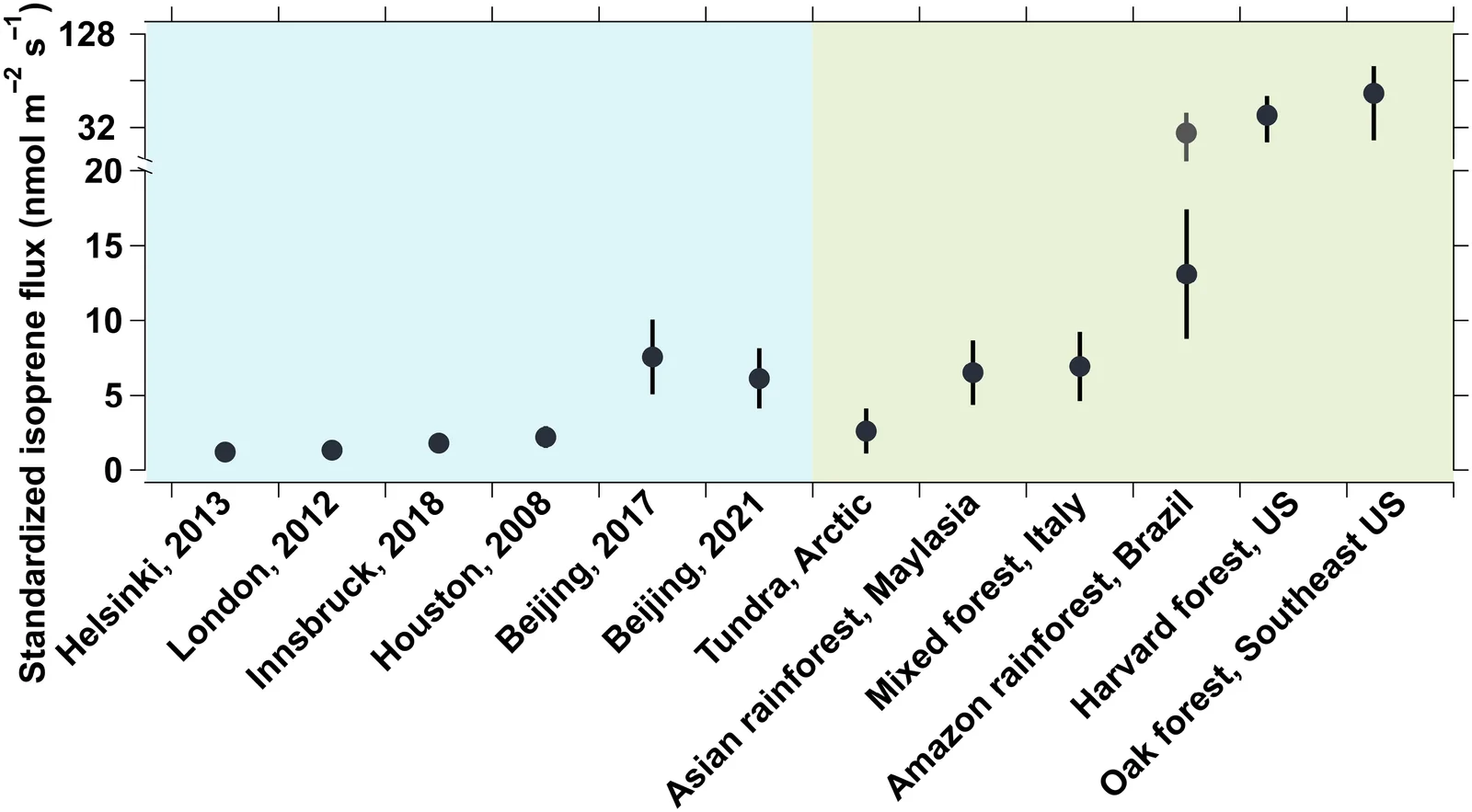
Standardized isoprene fluxes at a temperature of 30°C and PAR of 1000 μmol m^−2^ s^−1^ for urban regions and natural ecosystems. The blue and green shaded areas represent the standardized isoprene flux of urban regions and natural ecosystems in different climate zones, as presented in the published papers (*38*, *39*, *74*–*82*). The standardized isoprene flux in London in 2012 is estimated by the daytime average flux in August (*75*).

Our findings highlight that biogenic emissions are an important source of urban air pollution in Beijing. The compositions of BVOC emissions can be complex, but generally, isoprene emissions are shown as the dominant player for ozone chemistry in Beijing (Fig. 2). Isoprene is also thought to play the largest role in the global BVOC budget (*16*, *37*). To place our results within a global context, we compare our results to other available measurements in urban regions and natural ecosystems after normalizing isoprene fluxes to standardized conditions (Fig. 3 and fig. S9). The standardized isoprene flux in our study (6.13 nmol m^−2^ s^−1^) is comparable to earlier measurements in Beijing in summer of 2017 (7.57 nmol m^−2^ s^−1^) (*38*), indicating consistently high emissions in Beijing. The isoprene flux in Beijing is at the high end, compared to other cities with available flux measurements. Isoprene emissions observed in Beijing are unexpectedly close to those reported for some mixed temperate forests (*39*). Simultaneously, multisite observations in Beijing confirm that high isoprene concentrations are ubiquitous in the urban region, with levels comparable to or exceeding surrounding rural and mountainous regions (fig. S19). The vegetation cover rate in urban Beijing has increased steadily over the past two decades, as indicated by the normalized difference vegetation index (NDVI) (fig. S20), with an average rate of 2.2% year^−1^. However, it is not substantially higher than that in other urban areas (table S5). Isoprene emissions in urban Beijing may continue to rise in the future as the Beijing local administration persists in its efforts to green the city (*40*).

**Fig. 4.**
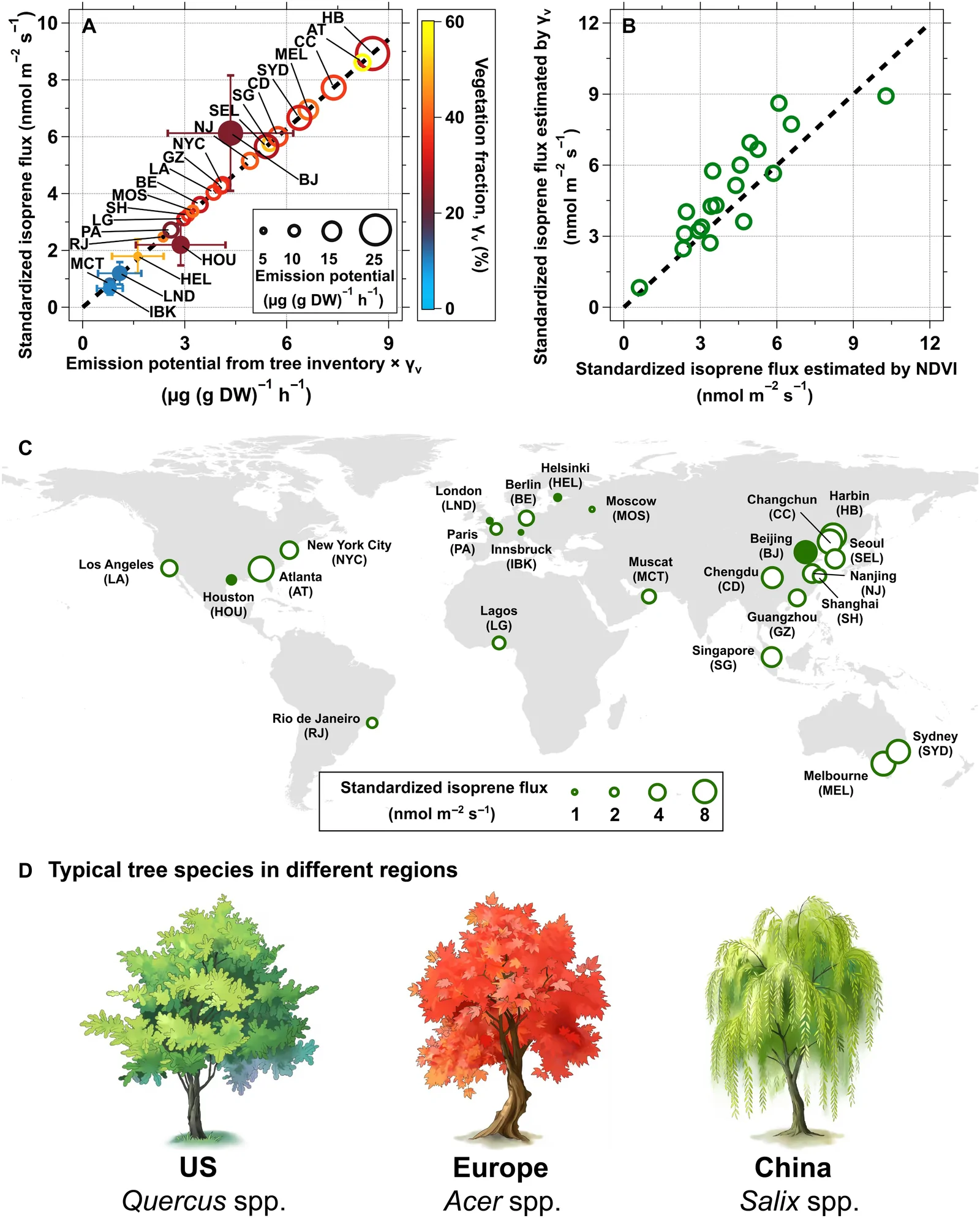
Estimated standardized isoprene emission flux of cities globally. (**A**) Scatterplots of the observed standardized isoprene flux versus the products of average isoprene emission potential and vegetation cover rate (γ_v_) for different cities. The solid circles represent data points obtained from flux observations around the world (*74*–*77*), and the hollow circles represent the predicted results for other cities. The black dashed line is the fitted results obtained using the weighted orthogonal distance regression (ODR) method based on the solid circles, with the slope representing leaf biomass density (*D*) determined at 257 g m^−2^. The symbol sizes are proportional to the average isoprene emission potential estimated from the tree inventory for corresponding cities. (**B**) Scatterplot of the standardized isoprene flux estimated by γ_v_ versus that estimated by the NDVI. (**C**) Geographical distribution of standardized isoprene flux for the surveyed cities. The symbol size is proportional to the measured (solid circles) or estimated (hollow circles) standardized isoprene flux for corresponding cities. (**D**) Typical urban tree species in different regions (US, Europe, and China). The specific tree species shown in (D) are *Quercus* spp., *Acer* spp., and *Salix* spp. (left to right). “spp.” represents the generalized reference to members of the genus.

To understand the main drivers of high isoprene emissions in Beijing, we link the isoprene flux to the average isoprene emission potential estimated from urban tree composition and vegetation cover rates (γ_v_) for each urban site, as shown in Fig. 4 (Materials and Methods and table S5). We derive a good linear relationship (*R*^2^ = 0.89) for the standardized isoprene emission flux with the average emission potential multiplied by γ_v_ (Fig. 4A), deriving the regression slopes as a constraint for biomass density (*D*) for urban trees. The obtained *D* (257 g m^−2^) lies well within the range of typically mixed temperate forests (189 to 323 g m^−2^) (*41*). With the obtained linear relationship, we estimate the standardized isoprene flux using reported urban tree inventories with collection of data for managed vegetation in streets or parks within the built-up areas of cities and γ_v_ for a total of 24 megacities globally (Fig. 4). Large variability is predicted for the standardized isoprene flux for the surveyed megacities, spanning in the range of 0.61 to 8.5 nmol m^−2^ s^−1^, well exceeding the overall uncertainty of our estimation method (∼58%). This indicates that the impacts of biogenic emissions on air quality for different cities can vary remarkably, which underlines the importance of localized assessment of these influences.

As mentioned above, of the cities with available flux measurements, the measured emission flux of isoprene in urban Beijing is the largest. The analysis in Fig. 4 demonstrates that the high isoprene flux in urban Beijing can be explained by the high-ranking average emission potential [18.9 μg (g DW)^−1^ hour^−1^] among all investigated cities (table S5), owing to large proportions of native trees known as isoprene emitters (i.e., *Salix babylonica*, 11.7%; *Populus tomentosa*, 9.1%) (*42*). This finding may apply to other cities in Northern China as reflected by similar or even larger average emission potential estimated for Harbin and Changchun as these cities have extensively planted willow and poplar trees over the past decades, given their rapid growth and climate resilience (*43*). Meanwhile, as illustrated in Fig. 4, isoprene emissions for a large portion of the investigated cities (mainly in Asia and Oceania) are predicted to be even higher than in Beijing, which may consequently result in potentially larger impacts on ozone pollution. These results imply that previous urban flux measurements of VOCs may only cover the lower end of the range of BVOC emissions for various cities, which advocates for more measurements in cities with potentially high impacts. A similar analysis using satellite-derived NDVI rather than γ_v_ yielded comparable results (Fig. 4B and fig. S22). On the basis of Fig. 4, we predict that emissions of isoprene in Guangzhou in southern China are comparable to those in Beijing. A parallel analysis of ozone temperature dependence in Guangzhou confirmed the anticipated strong influences of biogenic emissions (fig. S23).

**Fig. 5.**
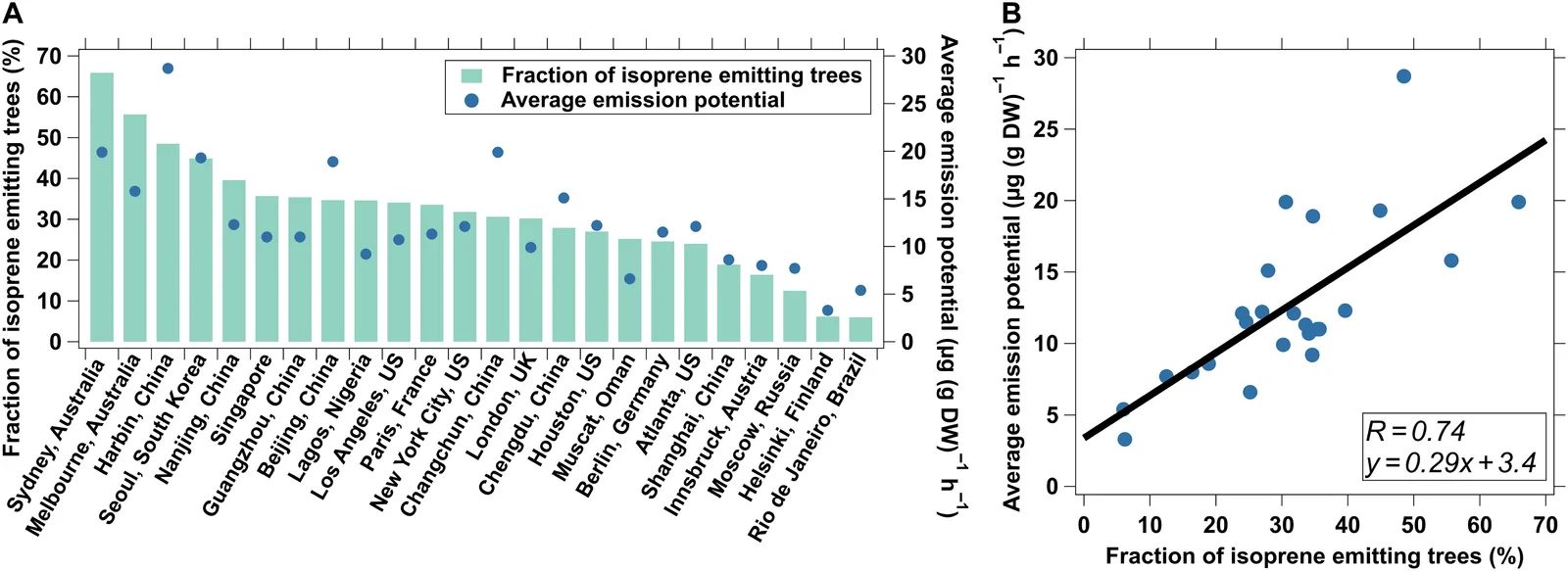
Average emission potential regulated by the fraction of isoprene emitting trees. (**A**) Category plot of the fraction of isoprene emitting trees and average emission potential for the investigated cities. (**B**) Scatterplot of average emission potential versus the fraction of isoprene emitting trees for all investigated cities. The isoprene emitting trees are defined as the tree species with an emission potential of ≥10 μg (g DW)^−1^ hour^−1^.

We find that only a small fraction (0.36) of the variance for the standardized isoprene flux is explained by γ_v_ (fig. S24), which implies that the emission potential estimated from urban tree inventories contributes substantially to the differences among cities. We demonstrate that emission potentials estimated from tree inventories are heavily controlled by the fractions of isoprene emitting trees (*R* = 0.74), which is defined as the fraction of urban trees with an emission potential of ≥10 μg (g DW)^−1^ hour^−1^ (Fig. 5 and table S5). The fractions of isoprene emitting trees in different cities span by more than one order of magnitude (6.0 to 66%), which is similar to the determined variability of isoprene emission flux for the surveyed cities. The important role of the single parameter, the fraction of isoprene emitting trees, in determining isoprene emission strength in urban regions has broad implications. First, replacing a small fraction of isoprene emitting trees can notably reduce the overall emissions of isoprene. Taking Beijing as an example, a megacity with 35% of the trees as isoprene emitters, replacing 10% of the trees from isoprene emitting trees with non-isoprene emitters would reduce at least 29% of isoprene emissions in urban Beijing. If the differences in isoprene emission potentials at the genus or species level can be considered using robust data, the reduction percentage of isoprene emissions can be even higher. As noted by previous evaluation (*16*, *44*), the existing emission capacity data of isoprene at species level in the literature might be strongly affected by environmental conditions during measurements and may not be representative of real-world differences of trees at species level. This implies the essential need for careful quality assurance of emission capacity measurements for urban trees. Second, the simple relationship of the fraction of isoprene emitting trees versus emission capacity shown in Fig. 5 provides a straightforward approach to determining the emission capacity of other cities. The determined emission capacity can be further used to adjust biogenic emissions estimated by regional models that currently rely on a limited number of plant functional types for emission factors (*22*).

The global comparison of cities highlights the implications for air quality management that can arise from tree selection in urban greening. The unexpectedly large impact of urban vegetation in Beijing, and potentially many other cities, is the result of planting trees with a high emission potential of isoprene in urban regions. Global warming (*45*) and larger urban heat island intensity (*46*) due to city expansion (*47*) are expected to induce enhancements of BVOC emissions. Other environmental stressors, such as drought (*48*, *49*), foliage damage caused by air pollution (*50*), and soil or water contamination, and extreme weather events (*51*), may also affect BVOC emissions. However, these effects remain poorly understood, and they are not explicitly considered in this study. Further urban greening activities (*52*) could also boost this change. Our results show that the replacement of a small fraction of isoprene emitting trees in urban regions can cut biogenic emissions considerably. However, the impacts of urban greening management on VOC emissions have not received enough attention in most cities. For example, the National Forestry and Grassland Administration of China issued a directive on urban greening to consider tree species diversity, aiming for more native and local tree species (*53*). However, many native trees are well-known isoprene emitters in both China and other regions, such as willow (*Salix* spp.) and poplar (*Populus* spp.) in Northern China, oak (*Quercus* spp.) in the US, and eucalyptus (*Eucalyptus* spp.) in Australia. In addition to urban greening in existing cities, planners of large-scale urban developments (e.g., Xiong’an New Area in China and NEOM in Saudi Arabia) may wish to consider urban tree selection in terms of VOC emissions to alleviate future air-quality management challenges.

## MATERIALS AND METHODS

### Flux measurements

The flux measurement site, situated on a 325-m meteorology tower (39°58′N, 116°22′E) near Beijing’s city center, features an integrated system that combines an open-path gas analyzer and a sonic anemometer (IRGASON, Campbell) both operated at 10 Hz. Positioned at 102 m on the tower’s southeast side to capture prevalent southeast winds during the summer of 2021, the system effectively measures high-frequency wind speed, CO_2_, and H_2_O mixing ratios. Adjacent laboratory facilities have been established using two sea containers.

Air samples are transported to the laboratory via ∼150 m of half-inch Teflon tubing using a vacuum pump with a flow rate of 45 liters min^−1^, resulting in a lag time of around 12 s. A PTR quadrupole interface time-of-flight mass spectrometer (Ionicon Analytik) uses hydronium ion chemistry to measure VOC concentrations. Calibration procedures include automatic zero calibration every 30 min and daily calibration using a 39-component gas standard (Apel & Riemer). Uncalibrated ions are quantified using a method outlined by Sekimoto *et al.* (*54*). The instrument achieves typical sensitivities ranging from 1300 to 2700 cps ppbv^−1^, with detailed setup parameters available in previous papers (*55*, *56*). To accurately quantify the isoprene signal (C_5_H_8_H^+^) in PTR-TOF, we corrected for interferences from the fragmentation of long-chain aldehydes and cycloalkanes (*57*). The evaluation results (fig. S10) indicated that fragmentation interferences have only a minor impact on flux determination (∼3.5%) but exert a much larger influence on the daytime concentration dataset (∼17%).

### VOC flux calculation and footprint estimation

VOC flux calculation and quality control are summarized in a workflow sketch depicted in fig. S1. These processes were performed by an open-access tool for EC analysis (InnFlux) (*58*). The lag times for VOCs were determined using the cumulated covariance method for each VOC species to ensure a more reliable estimation of lag time compared to individual covariance methods (fig. S2). The spectral analysis highlighted that our PTR-TOF system offers a reliable time response for measuring EC flux of VOCs with a small high-frequency loss (4% for toluene) (Supplementary Materials).

On the basis of the measured high-frequency wind speed and VOC data, the surface EC flux (*F*) was calculated at an average interval of 30 min by the following equation

F=w′c′¯ (1)

where the *w*′ and *c*′ denote fluctuations in vertical wind speed and concentration variation of certain VOC species.

As detailed in our earlier investigations (*59*), we estimated the flux footprint using measured wind data and urban underlying surface parameters with a 2D parameterization model (*60*). The estimation indicated that the 90% flux footprint encompassed ∼70 km^2^ of urban Beijing, covering residential areas, commercial districts, and urban green spaces, thereby capturing a representative spectrum of the city’s major functional zones.

### Source analysis from VOC flux

We applied PMF to the VOC flux dataset to resolve and quantify the major emission sources. PMF is a receptor model commonly applied in the source apportionment of environmental pollutants (*61*, *62*), which can be described as follows (Eq. 2). Flux-based PMF uniquely captures source profiles that are more physically meaningful and more representative of actual emission characteristics than concentration-based approaches as it effectively circumvents the interference from secondary chemical processing and regional transport. Consequently, this approach not only addresses the inherent limitations of concentration-based PMF in resolving reactive species and oxygenated VOCs (OVOCs) but also greatly enhances the robustness and reliability of source apportionment results (*27*).

The analysis was performed using the PMF Evaluation Tool (version 3.05) within the Igor Pro environment (*62*). The detailed PMF analysis has been explicitly documented by He *et al.* (*27*)

$$X_{\mathrm{ij}}=G_{\mathrm{ij}}F_{\mathrm{ij}}+E_{\mathrm{ij}}$$ (2)

*X* denotes the input flux matrix, whereas the output matrices *G*, *F*, and *E* represent the time series, the selected model solution profiles, and the residual matrix, respectively. The indices *i* and *j* correspond to the time series and mass dimensions for the input VOC species.

In total, fluxes of 193 VOC species at a 30-min resolution were included as inputs. The uncertainty matrix accounted for both PTR-TOF measurement errors and random uncertainties associated with the eddy covariance fluxes. The variation in the ratio of *Q*/*Q*_exp_ with increasing factor numbers was examined to identify the optimal factor solution, where *Q* represents the PMF objective function and *Q*_exp_ denotes its expected value under ideal conditions. After assessing a range of factor solutions, a five-factor model was identified as the most robust and interpretable. Factor identification was carried out through correlation analyses with characteristic tracers and comparison with localized source profiles. Detailed factor profiles and spectra used for the identification are provided by He *et al.* (*27*).

The five resolved factors were interpreted as follows: (1) biogenic emissions, (2) vehicle-related emissions, (3) VCP-related (OVOCs-rich), (4) VCP-related (aromatics-rich), and (5) cooking and household source (fig. S4A). For subsequent analyses, the two VCP-related factors were combined into a single VCP-related source category. Fluxes of VOC species not directly measured were estimated using localized source profiles, as detailed in the Supplementary Materials (text S2). Factors (2) to (5) were grouped together as anthropogenic emissions in our analysis.

### Other supporting measurements

In addition to VOC flux measurements, the concentrations of ozone (T400, Teledyne API), NOx (T200, Teledyne API), and ambient temperature were concurrently measured at both ground level and the 102-m platform at the flux tower site. Radiation parameters, including solar radiation, photolysis frequencies (PFS-100, Focused Photonics), and photosynthetically active radiation (PAR), were measured on the ground. These data were used as the input data for the box model and for other analyses in this work, e.g., the determination of the standardized isoprene flux.

Ozone data from ground observations at 12 monitoring stations in CNEMC network during our campaign period are used in this study. These stations are mainly located in urban areas of various districts in Beijing, reflecting the overall ozone pollution status of the entire urban area of Beijing.

Historical VOC concentration data (acetone, methyl ethyl ketone, benzene, toluene, and isoprene) in urban Beijing during summertime were obtained from the previous measurements conducted at the Peking University (PKU) site (*63*, *64*) and the flux tower site (*65*). These VOC species were measured either using online gas chromatography–mass spectrometry (GC-MS) systems or a PTR mass spectrometer. Measurements at multiple sites in Beijing (a total of 27 sites) for canister samples collected in 2010 are also used. These canister samples were analyzed using an offline GC-MS/FID (flame ionization detector) system (*63*, *66*).

### Atmospheric chemistry simulations using box and WRF-Chem models

A 0D box model based on the Framework for 0-D Atmospheric Modeling (F0AM) v3.1 (*67*) is used in this study to simulate ozone concentrations and the RIR of ozone precursors. Here, the box model is driven by the Master Chemical Mechanism (MCM) v3.3.1. Measurements of meteorological parameters, photolysis rates, and trace gas concentrations are used as constraints for the box model. The RIRs for VOCs and NOx are derived from the simulations of the box model by changing the concentration of NOx and all VOC species by 10%, respectively. The influences of biogenic emissions on ozone are obtained from two parallel simulations: one with measured isoprene, monoterpenes, and MVK&MACR as constraints and the other one with concentrations of these species contributed only by anthropogenic sources (i.e., without biogenic emissions).

An additional box model simulation is performed with measured VOC fluxes as constraints, and modeled VOC concentrations are compared with measurements to explore the contributions of local emissions to VOC concentrations. We obtained reasonable agreement between modeled and measured VOC concentrations, e.g., aromatics (fig. S26), which indicates that VOC concentrations in urban Beijing are governed by local emissions, rather than advection or long-range transport. The details of the box model settings are described in the Supplementary Materials.

To comprehensively quantify the contribution of urban vegetation to the urban ozone budget while accounting for transport processes, we used the 3D chemical transport model WRF-Chem (version 4.1.2) to simulate ozone formation in Beijing and its surrounding regions. Two nested domains were used, with horizontal resolutions of 9 and 3 km covering most of northern China and the Beijing municipality with surrounding regions, respectively. Biogenic emissions were calculated online using the MEGAN model (version 2.1) (*16*), whereas anthropogenic emissions were derived from the Multi-resolution Emission Inventory for China (MEIC) (version 1.3) (*68*), with the addition of monoterpene emissions from anthropogenic sources (*69*). The Regional Atmospheric Chemistry Mechanism version 2 (RACM2) was used to simulate gas-phase reactions of organic and inorganic species in the regional atmosphere (*70*). Additional details on model configuration and sensitivity simulations are provided in the Supplementary Materials and our previous paper (*69*).

### Temperature dependence analysis

In this study, we investigate the temperature dependency of VOC flux and ozone concentrations by plotting the daytime (8:00 to 18:00) average for VOC flux and daily 1-hour maximum for ozone versus daily maximum ambient temperature (*T*_max_). Daytime averaging of VOC fluxes minimizes the influence of light variability and reduces the impact of short-term extremes, allowing the temperature dependence of VOC emissions to be largely isolated. The temperature dependency of VOC fluxes that are relevant to biogenic emissions is fitted using an exponential temperature function (Eq. 3) (*32*, *71*)

$$M\left(T\right)=M\left(T_{s}\right)\text{exp}\left[\beta \left(T-T_{s}\right)\right]$$ (3)

where *M*(*T*_s_) is the metric at a standard daily maximum air temperature *T*_s_ (°C), which was set to 25°C. β denotes the temperature response factor.

As the temperature dependence of ozone can differ substantially under different ozone formation regimes, we used the box model to determine the RIR of NOx and VOCs for ozone by changing concentrations of NOx or VOCs by 10% (Supplementary Materials), respectively. On the basis of the results in Fig. 2 (C and D) and figs. S12 to S14, we use a threshold of RIR for NOx lower than 0.2% %^−1^ to select the period with ozone mainly controlled by VOCs. Last, the temperature dependence of ozone for these periods is regressed using the same equation (Eq. 3) as OHR_flux_, and the obtained temperature dependence curves are compared to those of VOC flux. Using this approach, our findings suggest that ozone temperature dependence can be more accurately represented using a nonlinear (exponential) relationship after considering ozone chemical sensitivity (figs. S12, S13, and S23). This is in contrast to previous works that determined ozone temperature dependence by directly averaging daily data points and performing linear regression for the slope as the dependence parameter with a unit of ppb °C^−1^ (example in fig. S13) (*33*, *72*). To demonstrate the statistical superiority of the exponential fit over the linear fit, we use data from the Guangzhou case, where the chemical regimes are all within the VOC-limited regime, as shown in fig. S23. We calculated the root mean square deviation (RMSD) between the fitted curves and the data points for both methods to quantify the fitting performance. The RMSD values from the exponential fits were lower than the linear fits for both observational data (linear: ∼27; exponential: ∼20) and model data (linear: ∼21; exponential: ∼14). This indicates that, from a purely mathematical standpoint, the exponential fit provides a substantially better representation of ozone dependence on temperature within the VOC-limited regime compared to the linear fit.

Beyond its statistical advantage, the exponential fit is also preferable as it provides deeper mechanistic insight into the factors driving ozone concentrations, such as temperature-driven VOC emissions and ozone formation regimes. The fitted exponential scaling factors for ozone range from 0.082 to 0.094 (for both observed and modeled data), closely aligning with the expected relationship between VOC emissions and temperature (*16*).

### Emissions from urban greening for cities worldwide

Biogenic isoprene emissions are light and temperature dependent (*73*), making it improper to directly compare isoprene emissions under different environmental conditions. Here, we standardized the isoprene emission flux to the condition of temperature at 30°C and PAR at 1000 μmol m^−2^ s^−1^ (Eq. 4) (fig. S9) (*73*)

$$E_{\text{isoprene}}=E_{0,\text{isoprene}}*\gamma_{T}*\gamma_{P}$$ (4)

where γ*_T_* and γ*_P_* are the emission activity parameters for temperature and light, respectively, associated with the environmental conditions of the past 24 to 240 hours.

Following a previous study (*41*), the standardized isoprene emission in certain regions (e.g., forests) can be described by the following equation

$$E_{0,\text{isoprene}}=\varepsilon *D*\gamma_{v}$$ (5)

where *E*_0,isoprene_, *D*, and γ_v_ represent the standardized isoprene flux, the leaf biomass density, and vegetation cover rate for the region. ε denotes the average emission potential from the vegetation, which is determined by tree composition information from tree inventory and the reported emission potentials for various tree species (Supplementary Materials). The tree species assessment methodologies for determining average emission potential involve including as many tree species as possible from each tree inventory, depending on the number of tree species available in the reported datasets. For tree inventories with fewer than 100 species, all species are considered in the calculation. However, for more detailed inventories (e.g., Paris, with over 800 species), we limit calculations to the top 100 species (accounting for more than 94% of the total tree population in Paris) to ensure representativeness while reducing computational complexity. This approach also mitigates uncertainties arising from less abundant tree species with limited or unavailable emission potential data.

In this study, we apply Eq. 5 to estimate isoprene emissions in urban areas. We compiled all available isoprene flux data from direct measurements in urban environments (i.e., Beijing, London, Helsinki, Houston, and Innsbruck), along with tree inventory data and γ_v_ within the measurement footprints (table S5). A linear relationship was established (black line in Fig. 4A) between the measured standardized isoprene flux and the product of average emission potential and γ_v_ at each flux site (solid circles). This relationship is then extrapolated to predict standardized isoprene emissions for a total of 19 cities. The 19 cities spanning all six continents are representative megacities in different continents, with reported ozone pollution issues and available information for urban tree inventory. The urban tree inventory and vegetation cover rate information for these cities is mainly from the published literature and/or open-access government databases, which are provided in detail in the Supplementary Materials. In collecting this information, we made every effort to ensure that the area represented by the urban tree inventory aligns with NDVI and γ_v_ data, thereby ensuring the representativeness of our calculations. For example, in our data (both inventories and vegetation data), Paris, Lagos, and Sydney represent Paris City, Lagos Metropolis, and the City of Sydney, respectively (fig. S21). Furthermore, we encountered limitations due to the availability of data, which resulted in varying degrees of representativeness of the tree inventories. To address these challenges, we categorized the inventory data and explicitly acknowledged the associated uncertainties. Urban tree inventories were categorized on the basis of their coverage, with those including only street or park trees (e.g., Rio de Janeiro) considered less representative, whereas those encompassing both (S+P) offered a more comprehensive depiction of urban tree composition (table S6). Overall, most cities in our study were categorized as S+P, ensuring the overall representativeness of the data used in our analysis. In addition, we also performed a similar analysis using the NDVI as a surrogate parameter for vegetation cover rate (fig. S22). NDVI data, derived from satellite monitoring, serve as a widely accessible and consistently updated indicator for urban vegetation for global cities. Here, the summertime mean NDVI values in the urban built-up areas were obtained from the MOD13Q1 product from MODIS (250-m spatial resolution) for various cities (see the Supplementary Materials).

## Supplementary Materials

### The PDF file includes:

Supplementary Text S1 to S8 Figs. S1 to S26 Tables S1 to S6 Legend for dataset S1 References
