# Supplementary material for: Tree selection in urban greening shapes air quality for global cities
Source: Sci Adv. 2026 Aug 19;12(34):eaee5583. doi: 10.1126/sciadv.aee5583 (PMC13488892; doi:10.1126/sciadv.aee5583)
Supplement: Supplementary file 1 [file sciadv.aee5583_sm.pdf]

Science Advances
AAAS

Supplementary Materials for

Tree selection in urban greening shapes air quality for global cities

Xianjun He *et al.*

Corresponding author: Bin Yuan, byuan@jnu.edu.cn; Thomas Karl, thomas.karl@uibk.ac.at; Min Shao, mshao@jnu.edu.cn

Sci. Adv. **12**, eaee5583 (2026)
DOI: 10.1126/sciadv.aee5583

The PDF file includes:

Supplementary Text S1 to S8
Figs. S1 to S26
Tables S1 to S6
Legend for dataset S1
References

Other Supplementary Material for this manuscript includes the following:

Dataset S1

Supplementary Text

S1. Spectral analysis for flux data

Co-spectral density analysis provides the frequency distribution of co-spectrum for VOC concentration and vertical wind, which identifies the inertial subrange carrying most turbulent transfer energy. According to Fig. S3A, the inertial sub-range was about 0.02–0.4 Hz. We comprehensively investigated the co-spectrum density of VOC species with effective saturation concentration (c*) >10^6 μg m^{-3}. The investigated species showed similar distribution and followed the -7/3 slope quite well (*83*, *84*), similar to the sensible heat flux. Although the VOCs co-spectral density captured by our proton transfer reaction-time of flight mass spectrometer (PTR-ToF) showed an off-line trend at frequencies higher than 0.4 Hz, the density was orders of magnitude lower than the main energy-carrying range. The results show that the time response of our PTR-ToF system was adequate for VOC flux measurements.

To evaluate this spectral loss, theoretical transfer functions are commonly used to quantify high-frequency attenuation due to sensor separation, sonic path averaging, sensor path averaging, and tubing attenuation. We adopted the method outlined by Striednig et al. (*58*) to perform spectral analysis on VOC flux, resulting in the obtained attenuated co-spectrum. Then, the spectral loss can be quantified by comparing the attenuated co-spectrum with the calculated model co-spectrum (*83*), as depicted in Fig. S3B. The comparison showed that high-frequency loss was minor (<5%) for most VOCs, indicating our flux measurement captured most of the high-frequency spectrum.

S2. Correction of unmeasured VOCs for total VOC flux

In this study, the VOC emission flux was measured by PTR-ToF, which effectively detects OVOCs, aromatics and alkenes well (*28*, *85*). However, as key components of VOC mass, most alkanes and some small alkenes (i.e., ethene and acetylene) cannot be detected by PTR-ToF (*86*, *87*). To comprehensively evaluate the total VOC emission flux, we estimate the emission flux for these species from PMF results. Based on our PMF analysis results, five factors are resolved, including biogenic emissions, vehicle-related emissions, cooking & household source, and two VCP emissions (OVOCs rich and aromatics rich). According to measurements by previous studies, PTR-ToF can measure VOCs from biogenic emissions (e.g., isoprene and its oxygenated products) and cooking emissions well (*88–90*). However, VCP-related and vehicle-related sources may emit non-negligible alkanes and small alkenes (*13*, *91–93*). Thus, based on our VOC flux results, we can estimate the total VOC emission flux by adding the estimated contribution of alkanes and small alkenes for VCP-related and vehicle-related emissions using the following equation:

$$F_{total} = F_{VCP} * R_{VCP} + F_{Vehicles} * R_{Vehicles} + F_{detected} \qquad (Equation\ 1)$$

where F_{total}, F_{VCP} and $F_{Vehicles}$ represent the estimated total VOC flux, the measured VCP emission flux assigned by PMF analysis, the measured vehicle-related emission flux assigned by PMF analysis, respectively. $F_{detected}$ and R represent the VOC flux detected by flux measurement and the ratio of the combined emissions of alkanes, ethene, and acetylene to the combined emissions of OVOCs and aromatics ((alkanes + ethene + acetylene)/(OVOCs + aromatics)). These key ratios are all determined by the source measurements in China (*91*, *94*).

Table S2 summarizes the values of R derived from source measurements for VCP emissions and vehicle exhausts. For vehicle exhaust emissions, we use a ratio of 2.19 for gasoline vehicles reported by Wang et al. (*91*) because the majority of vehicles in Beijing are gasoline vehicles (*95*). For VCP emissions, we compared the ratio of 6 domestic VCP categories obtained from VCP source measurements and found large differences among them (from 0.01 to 2.34) (*94*). These categories include personal care products, printing inks, cleaning products, adhesives, coatings, and pesticides. We determined an average R of the six VCP categories by considering the emission fraction for each category from the VCP inventory in China (*96*), which can be considered as the best estimate using currently available information. Although the category of pesticide has the highest R, its emission is lowest among all categories (*96*). We evaluate the influences of uncertainty from the R parameter of VCP by setting the R parameter to personal care products (with the second highest R and contributing more than half of domestic VCP emissions) as the upper limits, and coatings (with the lowest R) as the lower limits.

Based on our estimation method with assumed source emission ratios, the total VOC flux during the campaign was estimated. The results show that on average 23% (18%–28%) of VOC flux was not detected by PTR-ToF and the estimated bulk OVOC flux ratio (i.e., OVOC/VOC flux) is 60% (56%-64%) (Fig. S5). Furthermore, based on the estimation of total VOC flux, corrected source apportionment results are shown in Fig. S6. The correction for vehicle-related and VCP-related emissions increased the total measured VOC flux by approximately 22% and 8%, respectively. The best estimates for their relative contributions are 24 ± 2% for vehicle-related emissions and 50% (46%–53%) for VCP-related emissions.

S3. Calculation of hydroxyl radical reactivity (OHR) for flux

The OH reactivity of VOCs (OHR_{conc}) is usually quantified based on the measured concentrations of VOCs, providing a way to estimate the relative importance of individual ozone precursors in the atmosphere (*97*, *98*). In this study, we further calculated the OH reactivity of VOCs via our measured emissions flux (OHR_{flux}), which is a more direct way to evaluate the importance of emissions for each individual ozone precursor (*26*, *30*, *99*). We calculated the OHR for the 199 species with detectable fluxes measured by PTR-ToF by the following equation:

$$OHR_{conc} = \sum k_{OH+VOC} * c_{VOC} \qquad (Equation\ 2)$$

$$OHR_{flux} = \sum k_{OH+VOC} * F_{VOC} \qquad (Equation\ 3)$$

where c_{VOC} and F_{VOC} are the average concentrations for VOC species and the average emissions flux for VOC species. k_{OH+VOC} is the reaction rate coefficient of VOC species with the OH radical, obtained from the literature or kinetic databases (Table S3). For the k_{OH+VOC} values that cannot be found in the reference, we use the median value of the rate coefficients (1.5×10^{-11} cm^3 molecules^{-1} s^{-1}) listed in Table S3 as the best estimated reaction rate constant for these species.

We also investigated the total OHR_{flux} (measured + unmeasured VOC species) by estimating the OHR_{flux} from the undetected species in our measurements. Based on the PMF analysis, the total molar flux of undetected VOC species has been estimated by the method mentioned above. Our calculation shows that only ~8% OHR_{flux} was undetected in our measurements.

Large discrepancies of k_{OH+VOC} among different VOC species result in variations in OHR among various emission sources with different chemical compositions. We determined the weighted average k_{OH} ($k_{OH,avg}$) for each emission source derived from PMF analysis using the following equation:

$$k_{OH,avg} = \sum k_{OH+VOC} * f_{[VOC]} \qquad (Equation\ 4)$$

where $f_{[VOC]}$ represents the fractions of individual VOCs within the emission factor.

S4. Isoprene emissions from urban vegetation in Beijing

4.1 Standardization of isoprene emission flux

It is known that biogenic isoprene emissions are light- and temperature-dependent (*32*), making it complicated to compare isoprene emissions under different environmental conditions. To address this issue and compare the isoprene emissions ($E_{isoprene}$) between Beijing and other urban areas or other ecosystems (e.g., forests, wetlands), we standardized the isoprene emission flux to the condition of temperature at 30 °C and Photosynthetic Active Radiation (PAR) at 1000 µmol m^{-2} s^{-1} as the model proposed by Guenther et al. (Equation 5) (Fig. S9) (*73*). The standardized isoprene flux ($E_{0,isoprene}$) normalizes for variability of temperature and light conditions, providing a basis for comparing the isoprene emissions across different studies.

$$E_{isoprene} = E_{0,isoprene} * \gamma_T * \gamma_P \qquad (Equation\ 5)$$

where γ_T and γ_P are the emission activity parameters for temperature and light, respectively, associated with the environmental conditions of the past 24 to 240 hours.

4.2 Variation of urban vegetation in Beijing

We use the normalized difference vegetation index (NDVI) to evaluate the change in urban vegetation in the past two decades. We show that NDVI for winter months increased from 0.1 to 0.2 between the years 2000 and 2020, whereas NDVI increased from 0.36 to 0.42 during summer (Fig. S20A). The trend of NDVI amplitude corresponding to the 1-year period of the wavelet analysis (Fig. S20B) increased by 16% over the same period. These results suggest that Beijing has been greening for the past 20 years as inferred from NDVI products, which agree well with previous studies (*100*). The urban greening in Beijing may further improve, as outlined in an official report by the local government of Beijing in 2017, which set the goal of becoming a national forest city by 2035 (*40*). This implies that the urban greening rate of Beijing's urban built-up areas will reach over 49% in 2035.

4.3 Representativeness of flux measurement in Beijing

The representativeness of the flux measurements is evaluated from the following perspectives.

First, in terms of spatial representativeness, the flux footprint covers ~70 km^2 in central Beijing, accounting for ~5% area within Beijing's six central urban districts (>11 million population). It includes a wide range of major urban functional zones with a composition broadly consistent with the overall built-up area (*27*, *101*). The average NDVI within the footprint is also comparable to the urban average, indicating that vegetation abundance is representative of city-scale conditions.

Furthermore, multi-site observations across Beijing show broadly consistent isoprene concentrations (Fig. S19), suggesting relatively homogeneous emissions at the city scale and supporting the representativeness of the flux measurements for urban biogenic emissions.

S5. Temperature dependence analysis

Previous studies have widely investigated the phenomenon of ozone increasing with temperature (T), attributing this trend to three major components: emissions, chemistry and stagnation conditions (*34*, *102*). Among these drivers, emissions still remain largely unclear, as the emissions of ozone precursors (e.g., VOCs) are difficult to quantify directly (*103*, *104*). Our direct VOC flux measurements provide a valuable opportunity to explore the temperature dependence of emissions.

In this study, we mainly focus on the ozone-related issue. Therefore, the temperature dependence analysis was performed using the measured daily maximum surface temperature and the daily maximum/average metric (concentration, flux or OHR) of pollutants in the daytime (8:00–18:00). The OHR flux was separated into biogenic and anthropogenic sources based on the PMF analysis.

The temperature dependence for biogenic-related parameters (i.e., isoprene flux, concentration and OHR_{flux}) is fitted by an exponential temperature function commonly applied to simulate the biogenic emissions (Equation 6)(*32*, *71*).

$$M(T) = M(T_s)\exp\big(\beta(T - T_s)\big) \qquad (Equation\ 6)$$

where *M(Ts)* is the metric at a standard daily maximum air temperature T_s (25 °C). β denotes the temperature response factor. Different from biogenic sources, we use a linear function to fit the temperature dependence of OHR_{flux} from anthropogenic sources.

The temperature dependence of ozone has been reported extensively (*33*, *34*, *72*, *102*, *105*). Ozone data is typically binned to certain temperature degree intervals (e.g., 1, 2 or 5 °C), and linear fits are performed on the investigated temperature range (see example in Fig. S13). The temperature dependence of ozone contributed by emissions of precursors can be separated into two terms, namely the dependence of ozone on changes in precursor emissions ($\frac{dO_3}{d[Emissions]}$) and the dependence of precursor emissions on changes in temperature ($\frac{d[Emissions]}{dT}$) (Equation 7) (*34*, *102*):

$$\frac{dO_3}{dT} \propto \frac{dO_3}{d[Emissions]} * \frac{d[Emissions]}{dT} \qquad (Equation\ 7)$$

The term $\frac{dO_3}{d[Emissions]}$ is usually called ozone sensitivity to precursors, i.e., NOx and VOCs. Based on this idea, increasing evidence shows that ozone formation regimes strongly affect the observed ozone temperature dependence, with much higher dependence on the VOC-limited regime and lower dependence on the NOx-limited regime (*33*, *34*). However, this influence of ozone sensitivity on temperature dependence has not been taken into consideration in deriving the ozone-temperature relationships from the observations or modeling results.

S6. Description of model simulations

6.1 Box model simulations

To better understand how precursor sensitivity influences ozone–temperature relationships, we simulated ozone concentration and relative incremental reactivity (RIR) of precursors by using a zero-dimensional box model based on the Framework for 0-D Atmospheric Modeling (F0AM) v3.157 (*67*). The chemical mechanism driving the model is derived from the Master Chemical Mechanism (MCM) v3.3.1 database developed by the University of Leeds. The input data include relative humidity, ambient temperature, pressure, concentrations of trace gases and VOCs, which were measured at the flux site and interpolated to a 5-min time resolution. Prior to the main simulation,

the model was spun up for 72 hours to establish steady-state concentrations for secondary pollutants. An empirically determined physical dilution rate (12 h^{-1}) was applied to prevent excessive buildup of long-lived species (*106*). We assessed the significance of biogenic emissions in daytime ozone formation by comparing two box-model scenarios: one constrained by the observed concentrations of isoprene, monoterpenes, and MVK&MACR, and the other constrained only by the anthropogenic contributions of these species, with the biogenic component removed. The anthropogenic contributions were estimated from the PMF results.

Here, we use the RIR of precursors to indicate ozone formation regime for the ozone temperature dependence analysis. In practice, the RIR[X] represents the relative change of photochemical ozone production over a certain period per percent change of precursor X (equation 8) (*107*).

$$RIR[X] = \frac{\frac{\Delta P_{O_3}([X])}{P_{O_3}([X])}}{\frac{\Delta[X]}{[X]}} \qquad (Equation\ 8)$$

where $[X]$, $\Delta[X]$, $P_{O3}[X]$, and $\Delta P_{O3}[X]$ represent the concentration or emission of precursor X, the change for concentration or emission of X, the production rate of ozone without changes of precursor X, and the change for production rate of ozone due to the alteration of precursor X, respectively. The RIR values for VOCs and NOx are derived from box-model simulations by changing the concentration of all VOC species and NOx by 10%, respectively.

With the modeled RIR from the box model, we calculate the daily averaged RIR of NO_x and VOCs by averaging over the period from 08:00 to the hour of daily 1-h maximum for ozone concentration for all individual days, to better represent the most critical period for ozone formation. A strong negative correlation between RIR for NOx and RIR for VOCs is obtained, which is mainly controlled by ambient NOx concentrations (Fig. S14). Based on this relationship, data with RIR for NOx < 0.2 % $\%^{-1}$ are classified as VOC-limited regimes in our study.

In addition to analyzing ozone sensitivity and precursor reactivity, we further evaluate the relative contribution of advection and long-range transport to VOC levels in the urban area of Beijing by incorporating the measured VOC emission fluxes (in nmol m^{-2} s^{-1}) into the F0AM box model. This was done by adding zero-order emission terms for each chemical compound, assuming instantaneous emission and mixing within a box defined by the boundary layer height. The boundary layer height (BLH) data were obtained from simulations using the Weather Research and Forecasting model coupled with Chemistry (WRF-Chem). Accordingly, the emission rates ($k_{Emission,i}$ in molecules cm^{-3} s^{-1}) in the flux-driven box model are expressed by Equation 9, based on the method described by Panji et al. (*108*) for incorporating BVOC fluxes into the model.

$$k_{Emission,i} = \frac{F_i * N_A * 10^{-9} * 10^{-6}}{BLH} \quad (Equation\ 9)$$

where F_i is the measured emission flux for compound i (in nmol m^{-2} s^{-1}), N_A is Avogadro's constant (in molecules mol^{-1}), and BLH is the boundary layer height (in m).

The comparison between VOC concentrations simulated from emission flux data and the observed concentrations of aromatic species (Fig. S26) suggests that local emissions were the primary source of VOCs during the campaign period, with minor contributions from advection or long-range transport for these compounds.

6.2 WRF-Chem simulations

The three-dimensional (3-D) regional chemical transport model, WRF-Chem (version 4.1.2), was employed to simulate air pollution over Beijing and its surrounding regions from 20 May to 30 June 2021. Two nested domains were set up using the Lambert Conformal Conic projection, with horizontal resolutions of 9 km (108 × 99 grid cells) and 3 km (100 × 109 grid cells). Both domains include 36 vertical layers from the surface to 50 hPa. The outer domain covers most of northern China, while the inner domain encompasses the Beijing municipality and its surrounding regions (*69*). The simulation results in the inner domain are shown in Fig. S17, where thin black lines represent the administrative boundary of Beijing and thick black lines delineate the urban built-up area. Biogenic emissions were calculated using the Model of Emissions of Gases and Aerosols from Nature version 2.1 (MEGAN v2.1), which has been incorporated into the WRF-Chem (*16*). However, recent research indicates that default MEGAN systematically underestimates biogenic volatile organic compound (BVOC) emissions in urban regions due to the mischaracterization of urban land use, especially its reliance on Leaf Area Index (LAI) data (*109*). To account for this bias, we applied the adjustment proposed by Gao et al. (*21*), which adds an extra 15% of total BVOC emissions of Beijing Municipality to its urban core based on vegetation survey data reported by Ren et al. (*110*). This adjustment substantially improved the agreement between modeled and observed isoprene concentrations in urban Beijing and successfully reproduced the observed diurnal variability. The anthropogenic emissions were derived from the Multi-resolution Emission Inventory for China (MEIC, version 1.3) (*68*), with the addition of monoterpene emissions from anthropogenic sources (*69*). We employed the Regional Atmospheric Chemistry Mechanism version 2 (RACM2) to simulate gas-phase chemical reactions (both organic and inorganic chemistry) in the regional atmosphere. Further details on the model configuration are available in our previous study (*69*).

The decision to adjust biogenic emissions rather than anthropogenic emissions is supported by observational evidence, as summarized below. First, the flux-based PMF analysis suggests that 95±2% of the observed isoprene originates from the biogenic factor (Fig. S4). Consistently, an independent estimate based on the nighttime

anthropogenic isoprene-to-CO flux ratio (Fig. S16B) indicates that anthropogenic sources contribute only 7±1% of the observed isoprene emissions, in close agreement with the PMF-derived estimate of 5±2%. Second, isoprene fluxes and concentrations show strong temperature dependence (Fig. S11A-B) and near-zero nighttime values in the absence of solar radiation, consistent with the characteristic light- and temperature-dependent behavior of biogenic emissions (*16*, *111*). Third, although anthropogenic sources such as vehicle exhaust and combustion processes have been reported to emit isoprene in urban environments (*76*, *112*), weak correlations between isoprene fluxes and traffic- and combustion-related tracers (Fig. S16) indicate that such sources play only a marginal role in Beijing. Overall, the available evidence suggests that uncertainties in biogenic emission estimates are the primary cause of the model underestimation, whereas missing anthropogenic emissions likely play only a minor role.

In the simulations, a "Base" scenario was conducted accounting for all emissions, along with two sensitivity cases: one excluding urban vegetation emissions within Beijing's built-up area (Case 1) and another excluding all biogenic emissions in the inner domain (Case 2). By comparing the Base scenario with the sensitivity cases, we estimated that urban vegetation contributes approximately 60% to the daytime peak isoprene concentrations in the built-up area (Fig. 18A-B). Building on this, WRF-Chem results indicate that urban vegetation emissions (Base − Case 1) and regional transport/background (Case 1 − Case 2) contribute an average of 12.8±6.8 ppbv and 9.4±4.4 ppbv, respectively, to the average daily peak 1-hr ozone in Beijing's built-up area (Fig. S18C-D). Notably, the combined contribution (22.2±10.2 ppbv) is comparable to our 0-D box model estimate of 18.5±8.6 ppbv. Furthermore, the ozone enhancement from urban vegetation shows strong spatial consistency across the built-up area (Fig. S17B), highlighting the broader representativeness of our results rather than localized effects. These results reveal the substantial contribution of urban vegetation to the urban ozone budget, consistent with findings from the 0-D box model, further reinforcing the representativeness and robustness of this conclusion.

S7. Comparison of isoprene emission potential for different cities

Biogenic isoprene emissions are influenced by various parameters, including plant species, leaf biomass density and plant species distribution (*16*, *113*). The ability to emit isoprene from different tree species can vary significantly, with variability up to three orders of magnitude (*114*, *111*, *115*). The isoprene emission potentials for specific tree species have been measured extensively from branch-enclosure/leaf-enclosure measurements (*41*, *116*, *117*). These measurements provide the emission rates expected for specific tree species at a temperature of 30 °C and photosynthetically active radiation (PAR) at 1000 μmol m^{-2} s^{-1}. There have been some available emission potential databases/literature compiled and some were updated periodically (*111*, *114*, *118*). In this study, all emission potential for different trees used in this study is derived

from the literature. However, some studies have suggested large differences in isoprene emission capacities between species, which is not accurate. Geron et al. investigated the emission potential of 18 North American oak species and species from six other isoprene-emitting genera, finding that reported differences in isoprene emission capacity were due to environmental conditions, measurement techniques, and a lack of environmental data in earlier studies, rather than true species differences (*44*). This implies that the variability in species-specific emission potentials within the same genus reported in the literature may be less pronounced than previously estimated. Therefore, we did not distinguish emission potential differences among species within the same genus and used genus-specific average emission potentials from the literature for individual tree species in our calculations, rather than species-specific values. To assess the reliability of genus-specific emission potential data, we compared average emission potential estimates derived from both genus-specific and species-specific emission potential data (Fig. S25). The comparison indicates that the majority of the data from both datasets show strong consistency, with most data points clustering around the 1:1 line. The mean relative difference between the two approaches is 33%. This demonstrates that genus-specific emission capacity data, while requiring less effort, can still yield accurate estimates of average emission potential. Using the genus-specific dataset, we identified tree species with an emission potential of ≥10 μg (g DW)$^{-1}$ h^{-1} as isoprene emitting trees and calculated their proportion within the urban tree population of each surveyed city (Table S5).

For a city, the average isoprene emission potential from urban trees (ε) can be obtained using the following relationship:

$$\varepsilon = \sum \varepsilon_{trees} \times f_{[trees]} \qquad (Equation\ 10)$$

where ε_{trees} is the standardized isoprene emission potential (temperature at 30 °C and PAR at 1000 μmol m^{-2} s^{-1}) of individual tree species, and $f_{[trees]}$ is the composition of tree species for the urban region of the city, typically derived from the city's tree inventory.

The tree inventories of the surveyed cities were collected from the literature or open-access websites. The detailed sources for this information are provided in Dataset S1. For the five urban sites with available flux measurements, we prioritize using the tree inventory within the footprint. If the footprint-specific inventory is not available in the literature, such as at the London site (*75*), we use the tree inventory for the broader Westminster district where the measurement site is located. For the 19 predicted cities in our analysis, when multiple urban tree inventories are available, we prioritize the one with the most detailed species composition, the highest number of trees, or the largest coverage area, ensuring it overall represents the entire city.

The ε can be related to the standardized isoprene flux (see definition in section 5.1) using the following equation (*41*):

$$E_{0,isoprene} = \varepsilon \cdot D \cdot \gamma_v \qquad (Equation\ 11)$$

where $E_{0,\ isoprene}$, D and γ_v represent the standardized isoprene flux, the leaf biomass density and vegetation cover rate for a region (footprint of measurement site) or a whole city. The vegetation cover rate for each city is obtained from city websites, government reports or published literature, which are summarized in Table S5.

Normalized Difference Vegetation Index (NDVI) is a well-established metric for evaluating the density of vegetation cover by remote sensing data (e.g., satellite spectrometric data) (*119*, *120*). To determine the average NDVI for certain cities, we use the global NDVI datasets and global urban boundary datasets to perform spatial analysis for certain cities. For the NDVI dataset, we utilize the MOD13Q1 product derived from MODIS, which provides global NDVI data with a time resolution of 16 days and a spatial resolution of 250 m (*121*). We compute the average NDVI for both measurement sites and surveyed cities within their respective boundaries (Table S5). For the measurement sites, we calculate the average NDVI within the footprint of flux measurements throughout the measurement period. For surveyed cities, the average NDVI in urban areas of targeted cities is calculated for the urban built-up areas during local summertime (Fig. S21): June to August for cities in the Northern Hemisphere and December to February for cities in the Southern Hemisphere. The boundary of the urban built-up area for each city is obtained from the global urban boundaries (GUB) dataset for the year 2018 (*122*), which was determined from the 30 m global artificial impervious area (GAIA) data.

S8. Uncertainty analysis

VOC flux measurement uncertainty. The uncertainty in VOC flux measurements consists of three main components (*103*): (1) uncertainty in VOC concentration measured by PTR-ToF, (2) systematic errors in measured fluxes, which are considered minor, and (3) random errors in flux measurements, which average 24% across all species, following the methods of Langford et al. (*123*).

For VOC concentration measurements, the uncertainty for compounds calibrated using standards is estimated to be 15%. For uncalibrated ions, calibration factors are estimated based on molecular properties, with an associated uncertainty of 50% (*54*). Given that calibrated and uncalibrated species contribute approximately 85% and 15% of the total detectable VOC concentration, respectively, the overall uncertainty in VOC concentration measurements using PTR-ToF is estimated at 20%. Consequently, the general uncertainty in VOC flux measurements is estimated to 44%.

Uncertainty for determination of the isoprene standardized flux. Isoprene concentration is calibrated using standards, with an uncertainty of 15%, while the random error in flux measurements is 16%. The uncertainties in temperature and light (PAR) measurements are both estimated to be 5%. The combined uncertainty in the standardized isoprene flux is determined as:

$$\frac{\delta E}{E} \leq \sqrt{\left(\frac{\Delta F_{isoprene}}{F_{isoprene}}\right)^2 + \left(\frac{\Delta T}{T}\right)^2 + \left(\frac{\Delta PAR}{PAR}\right)^2} = 33\% \qquad (Equation\ 12)$$

where $\frac{\Delta F_{isoprene}}{F_{isoprene}}$, $\frac{\Delta T}{T}$ and $\frac{\Delta PAR}{PAR}$ represent the uncertainty of the measured isoprene flux, temperature, and photosynthetically active radiation, respectively.

VOC flux-based PMF analysis. The uncertainty in the PMF analysis is evaluated using the bootstrap method with 100 iterations (*124*). The uncertainties for the resolved sources are as follows: (1) biogenic emissions factor—5%, (2) vehicle-related factor—26%, (3) VCP: OVOCs-rich factor—9%, (4) VCP: aromatics-rich factor—20%, and (5) cooking & household source factor—17%. The overall uncertainty associated with the PMF analysis is ~15%.

OH Reactivity for VOC flux. As detailed in Text S3 (SI), we assigned exact k_{OH} to 100 species based on the NIST dataset and literature, while we estimated k_{OH} values for unidentified VOCs and those with unknown rate constants (99 species) using a weighted-average *k* value to calculate the OHR. Based on our statistics, the 100 species with known k_{OH} can contribute ~96.5% of the measured total VOC flux, while those with unknown rate constants contribute ~3.5%.

The uncertainty associated with assigning exact k_{OH} values to these 100 species is estimated to be 15%. In contrast, the estimation of k_{OH} for unidentified VOCs and those with unknown rate constants may introduce an uncertainty of up to 200% (*125*). The total uncertainty in determining k_{OH} is calculated using the following function:

$$\delta k_{OH,total} = (f_{known} * \delta k_{OH,known}) + (f_{unknown} * \delta k_{OH,unknown}) \quad (Equation\ 13)$$

where f_{known}, $\delta k_{OH,known}$, $f_{unknown}$ and $\delta k_{OH,unknown}$ are the relative contributions and the uncertainty of determining k_{OH} of VOCs with known and unknown k_{OH} values, respectively.

Thus, the uncertainty in determining k_{OH} for all VOC species is ~22%. The overall uncertainty in the amount of OHR_{flux} is ~49%, obtained by combining the uncertainty in VOC flux measurement (44%) and the uncertainty in determining k_{OH} (22%). The uncertainty of relative contributions to OHR_{flux} for different emission sources or compound categories in Fig. 1 is significantly lower than ~49%, due to the interplay between error cancellation and propagation effects.

0-D chemical model for ozone. Gaussian error propagation, following the approach described in Lu et al. (*106*), was used to evaluate the model performance and assess uncertainties associated with various measurement parameters, including VOCs, trace gases, and meteorological variables, which range from 0% to 20%, while reaction rate uncertainties are ~20%. Additionally, the dilution rate was determined based on sensitivity tests comparing simulated and observed concentrations of unconstrained OVOCs and ozone. The uncertainty in determining $k_{dilution}$ is estimated to be around

10%. Considering these factors, the overall uncertainty of the box model is 30%–50% (*126*).

The temperature analysis using the exponential fit. The temperature dependence for ozone is determined by the exponential fit and used to predict ozone increases for future extreme high temperatures in our analysis. The uncertainty of the coefficient obtained from the exponential fit was determined by calculating the 95% confidence interval, which resulted in an estimated uncertainty of 19%.

Estimating isoprene emission flux in cities worldwide. We performed the regression-based extrapolation of isoprene emission flux to other cities based on Equation 4 in the main text. This estimation is based on an important assumption: Measured fluxes at the measurement sites are representative of each city as a whole, which assumes a similar and uniform distribution of trees throughout each urban area. This means that the estimated flux represents the total emissions from urban trees included in the city's tree inventory, assuming they are uniformly distributed across the urban area. It does not account for emissions from non-urban-managed trees, such as the extensive natural forests surrounding Atlanta or trees within large national parks.

The uncertainty in isoprene flux estimations for cities worldwide stems from:

1. Uncertainty of standardized isoprene fluxes at urban sites is 34% as estimated earlier in this section.

2. The accuracy of the average emission potential for cities is influenced by uncertainties in tree composition within urban tree inventories and the use of genus-specific emission potentials in its estimation. The uncertainty in urban tree inventories is estimated to be 25% for inventories that include both street and park trees, while it increases to 50% for less representative inventories that account for only street trees or park trees (Table S6). The uncertainty associated with genus-specific emission potentials can be separated into two components: (1) the inherent uncertainty in species-specific emission potentials (15%), and (2) the discrepancy between estimates based on genus-specific and species-specific emission potentials, quantified as 33% based on the mean relative difference (Fig. S25). These uncertainties are combined using standard error propagation (in quadrature), resulting in an overall uncertainty of 36% for genus-specific emission potentials. Incorporating this with the uncertainty in urban tree inventories, the overall uncertainty in the average emission potential is estimated to be 46%.

3. The uncertainty associated with the vegetation cover rate (γ_v) or NDVI data for each city. It should be noted that vegetation cover rates for different cities are derived from various literature sources or government websites, which may introduce inconsistencies due to differences in data collection methods, spatial resolutions, and temporal variations. We consider the uncertainties in NDVI and γ_v data to be comparable. Based on the standard deviation of NDVI values within the statistical regions of all cities, the associated uncertainty is estimated to be 28%.

4. Uncertainty arising from the linear regression method used to determine biomass density (*D*) for urban trees. *D* is derived using data from five measurement sites, but it appears to be heavily influenced by the high value observed in Beijing, as the other four sites are clustered around lower values. This discrepancy introduces potential errors, particularly when extrapolating to values beyond those observed in the measurement sites. Such extrapolation may not accurately reflect the biomass density in other urban contexts, leading to increased uncertainty in the estimation of *D*. To mitigate these uncertainties, we employ weighted orthogonal distance regression (ODR) (*127*), incorporating weights based on the uncertainties in standardized isoprene fluxes, γ_v, and the averaged emission potential (Fig. 4a). This approach ensures that the fitted *D* remains both scale-invariant and slope-invertible. The uncertainty associated with the weighted ODR regression is quantified as 14%, based on the standard deviation of the fitted coefficient. Beyond regression, *D* inherently varies due to species composition and canopy structure. Urban vegetation is similarly heterogeneous, but urban-specific reference values are lacking of reference values; thus, we adopt the range for mixed temperate forests (189–323 g m^{-2}) (*41*), corresponding to 26% relative uncertainty. Combining this with uncertainty from regression gives an overall *D* uncertainty of 30% when scaled by vegetation cover.

To estimate the total uncertainty in isoprene emission estimation, we combine the individual uncertainties using standard error propagation formulas. The total uncertainty (δE_{total}) can be expressed as follows:

$$\frac{\delta E_{total}}{E_{total}} \leq \sqrt{\left(\frac{\Delta F_{isoprene}}{F_{isoprene}}\right)^2 + \left(\frac{\Delta \gamma_v}{\gamma_v}\right)^2 + \left(\frac{\Delta \varepsilon}{\varepsilon}\right)^2 + \left(\frac{\Delta D}{D}\right)^2} \qquad (Equation\ 14)$$

where $\frac{\delta E_{total}}{E_{total}}$, $\frac{\Delta F_{isoprene}}{F_{isoprene}}$, $\frac{\Delta \gamma_v}{\gamma_v}$, $\frac{\Delta \varepsilon}{\varepsilon}$ and $\frac{\Delta D}{D}$ represent the total uncertainty in isoprene flux estimations, the uncertainties associated with site-level standardized isoprene fluxes, the estimation of the averaged emission potential, vegetation cover determination, and *D*, respectively.

Thus, the overall uncertainty in isoprene flux estimation is 70% for cities with both street and park tree inventories. If only street or park tree inventories are available, the uncertainty would be higher due to the increased uncertainty in emission potential.

Our uncertainty analysis underscores the need for caution when directly applying the predicted standard isoprene emission fluxes in subsequent analyses and interpretations. A fundamental assumption of our predictive approach is that the flux measurements at the sites represent the entire urban area, based on the premise of a uniform and similar distribution of trees across the city. However, given the inherently heterogeneous spatial distribution of urban vegetation, our predictions primarily reflect an estimation of the city's overall emission levels. The estimated flux represents the total emissions from the city’s urban tree inventory, assuming a uniform distribution.

Despite these limitations, our analysis places greater emphasis on the relative relationships of emissions across cities and the key factors driving urban biogenic emissions. By focusing on comparative emission patterns across different urban environments, our approach provides more robust insights with reduced uncertainty, offering a reliable basis for future research and policy development.

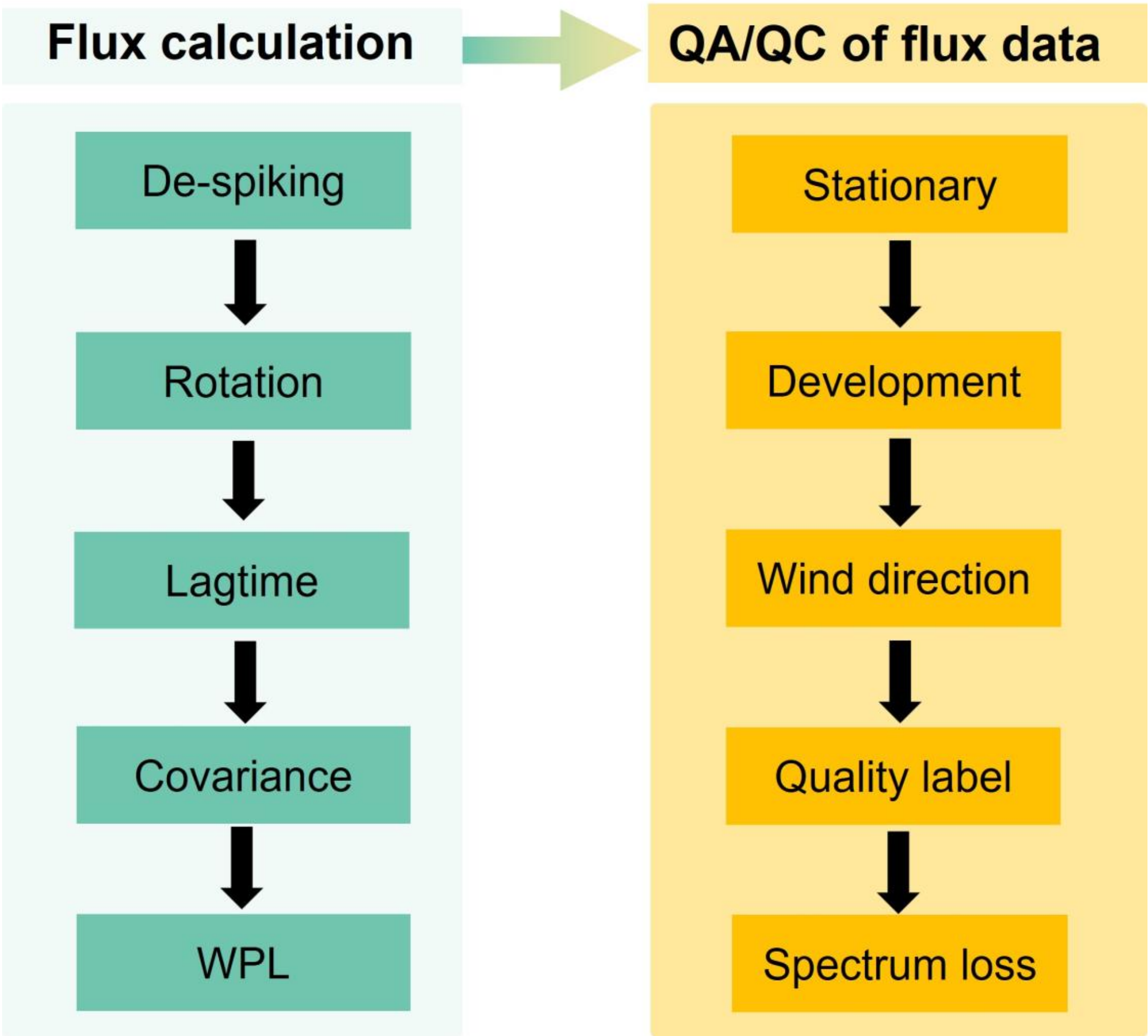

Fig. S1. Workflow of VOC flux calculation and quality assurance (QA)/quality control (QC) for VOC flux data. WPL: Webb, Pearman, and Leuning correction.

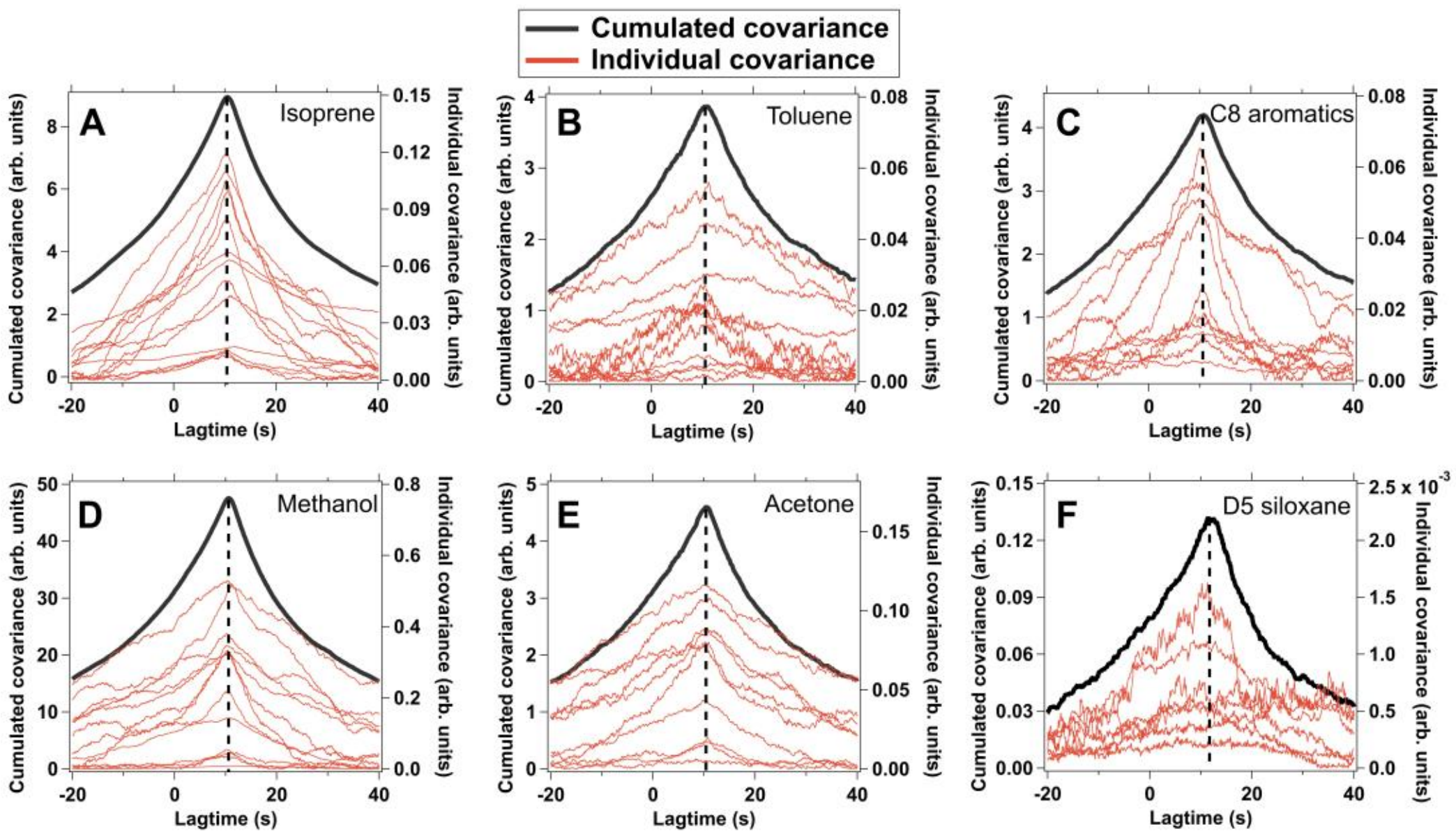

Fig. S2. The lag time of VOCs is determined by the cumulated covariance method. The lag time determination for (**A**) Isoprene, (**B**) Toluene, (**C**) C8 aromatics, (**D**) Methanol, (**E**) Acetone, and (**F**) D5 siloxane.

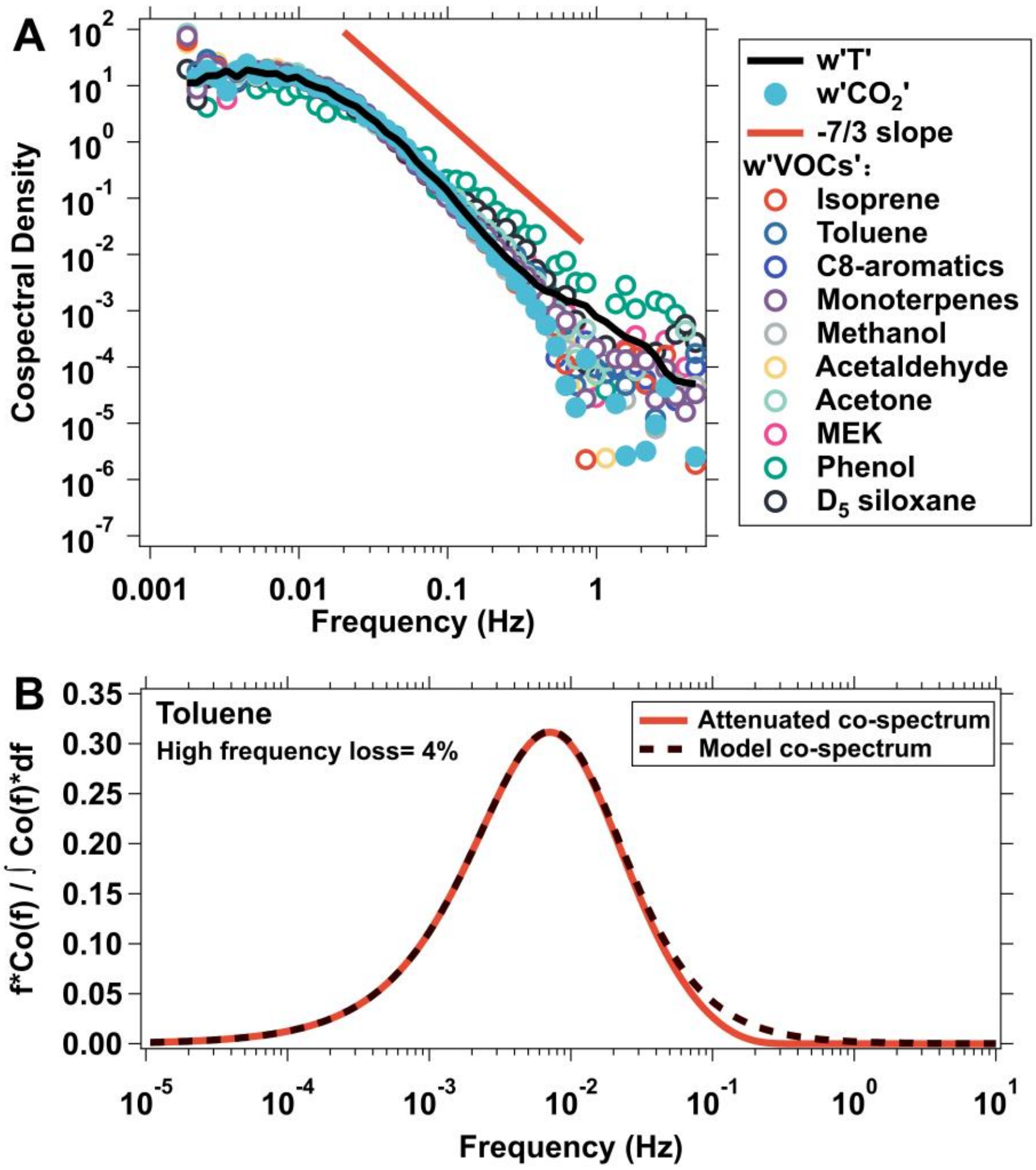

Fig. S3. Spectral analysis of VOC flux measurements. (**A**) The co-spectral density for fluxes of common VOCs species and sensible heat flux (w'T', in black dashed line) indicates the frequency distribution of unattenuated co-spectrum followed the -7/3 slope (solid black line) quite well during the inertial sub-range (0.02–0.4 Hz). (**B**) Modeled co-spectrum (black) and the attenuated co-spectrum (red). The calculated high-frequency attenuation indicates a 4% loss of flux signal at the high-frequency end.

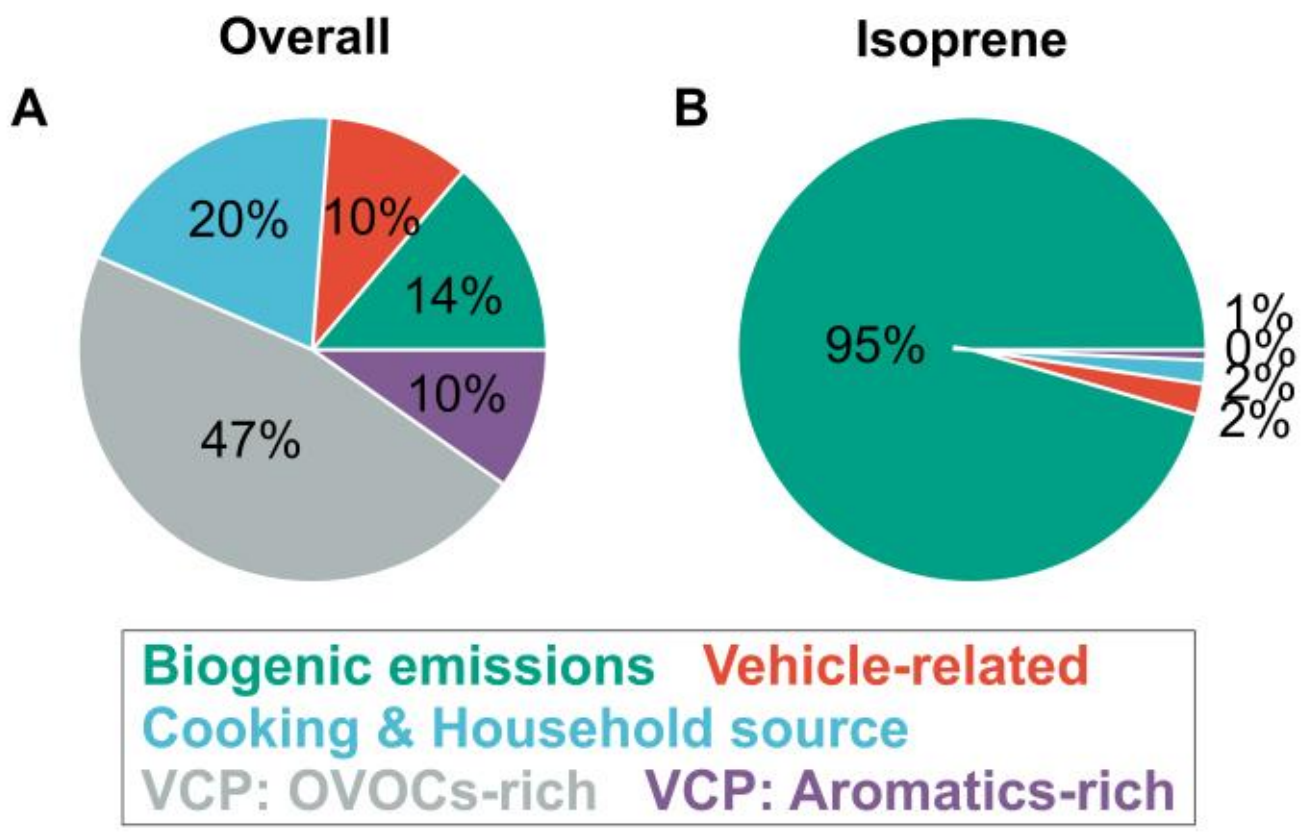

Fig. S4. Source apportionment results from flux-based PMF analysis. Contributions of the five PMF factors to (**A**) total measured VOC flux and (**B**) isoprene flux during the campaign.

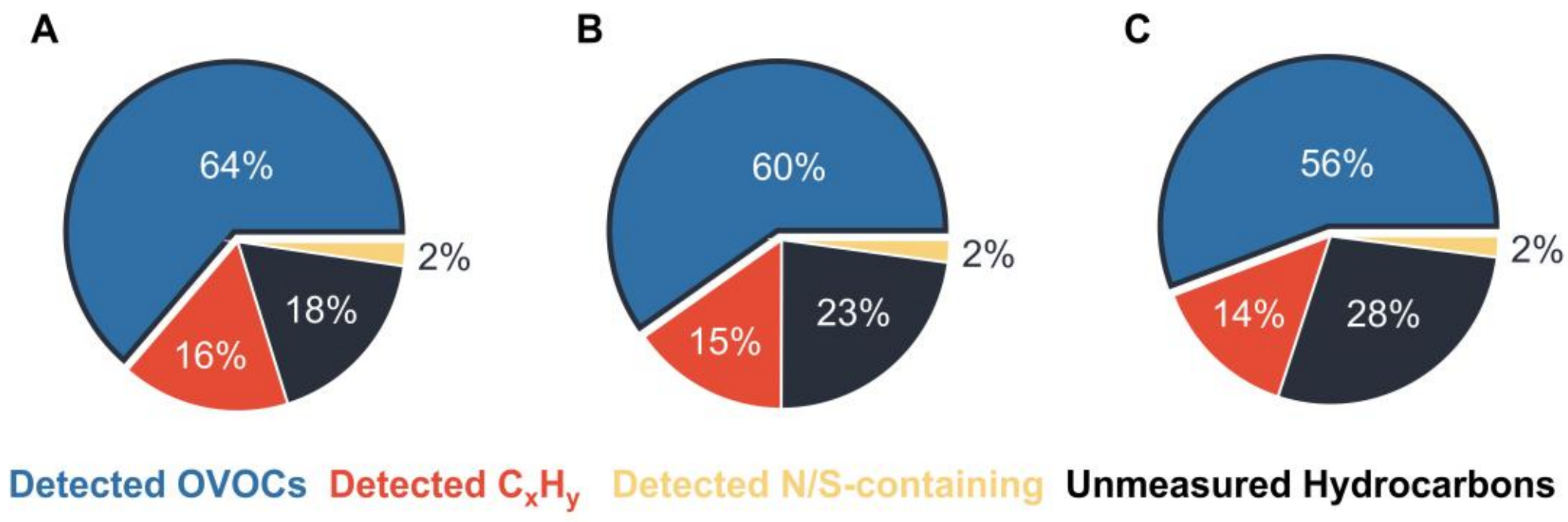

Fig. S5. The contributions of the four categories of VOC species to the corrected total VOC flux. (**A**)-(**C**) represent the lower limit, median and upper limit of the estimated total VOC flux, respectively.

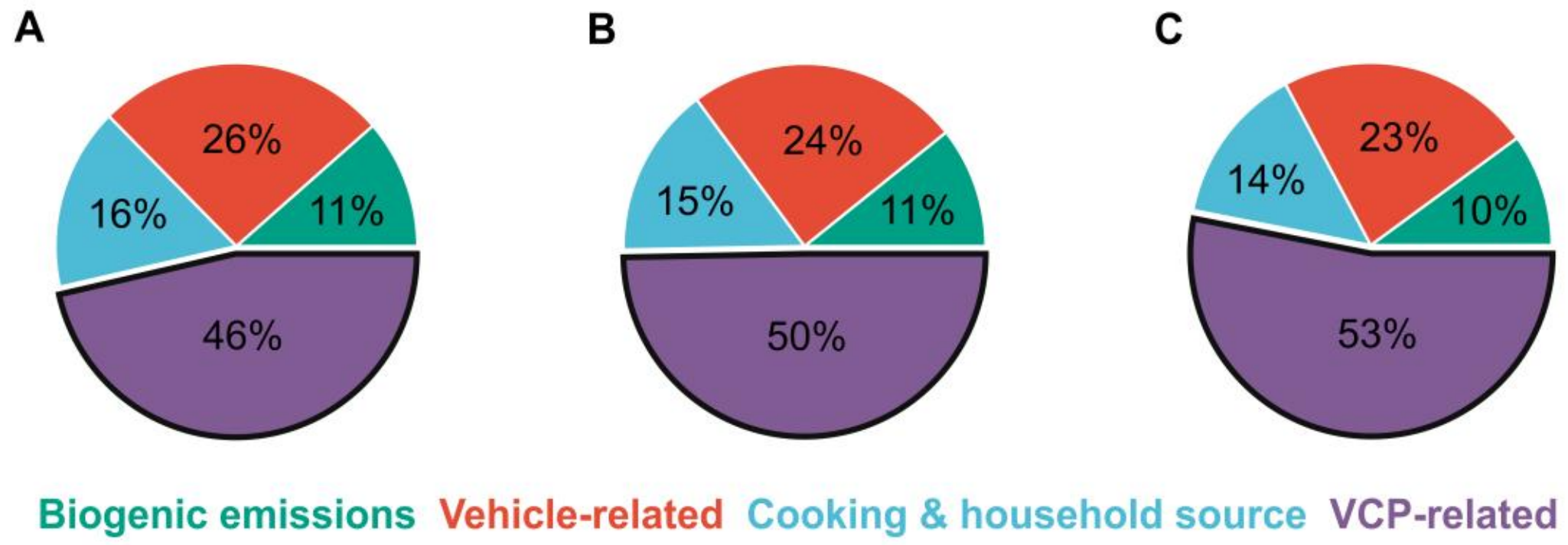

Fig. S6. The contribution of the four emission sources to the corrected total VOC flux. (**A**)-(**C**) represent the lower limit, best estimate, and upper limit of the estimated total VOC flux, respectively.

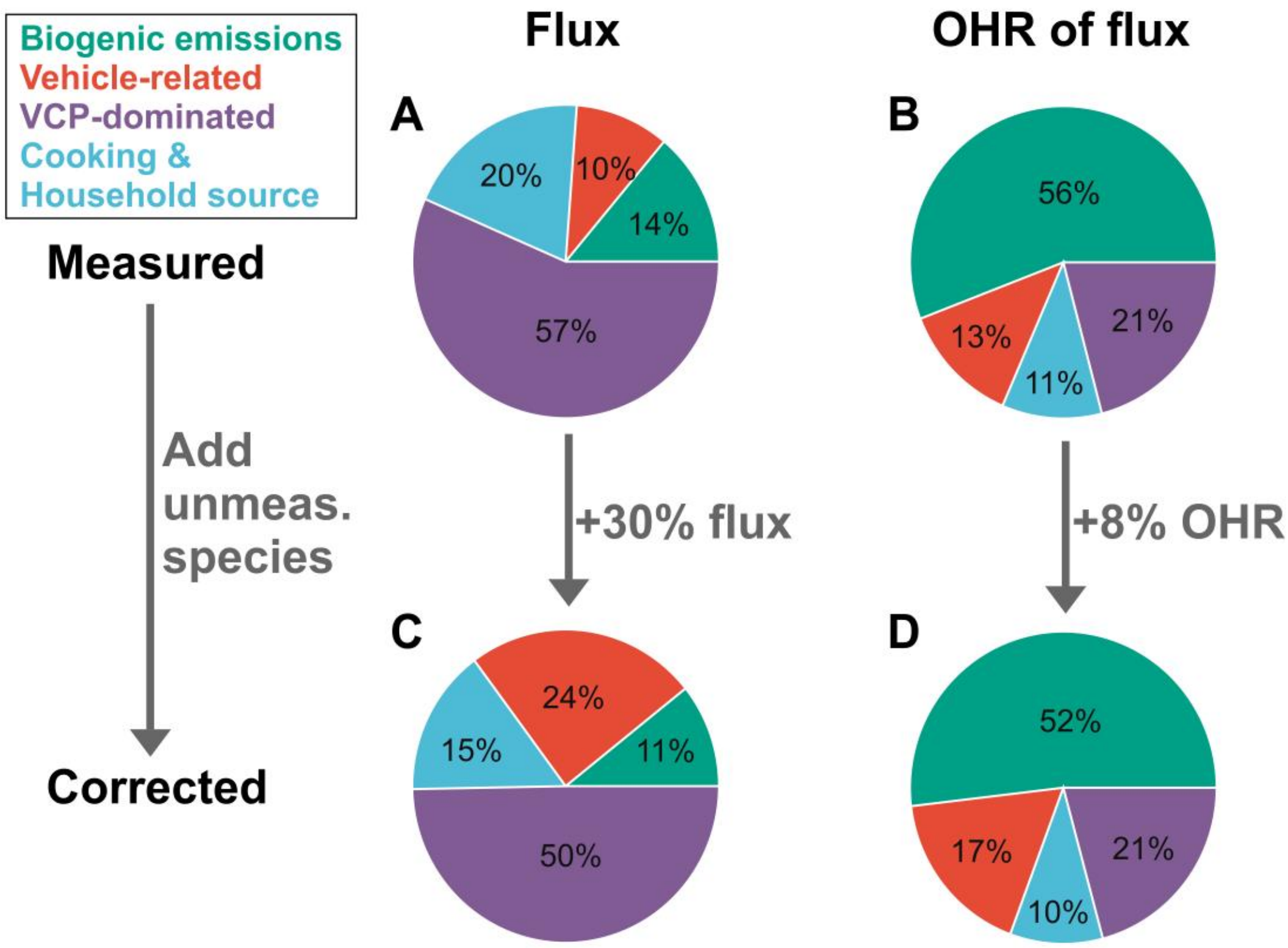

Fig. S7. **Contributions of emission sources to VOC flux and OHR of flux resolved by PMF analysis.** The contributions of the four emission sources from PMF analysis to (**A**) the total measured VOC flux, (**B**) the OHR of the measured VOC flux, (**C**) the estimated total VOC flux and (**D**) the OHR of the estimated total VOC flux after adding the unmeasured species. The VCP-related emission source is the combination of the two resolved factors (VCP-related: OVOCs-rich and VCP-related: aromatics-rich) in the 5-factor PMF solution.

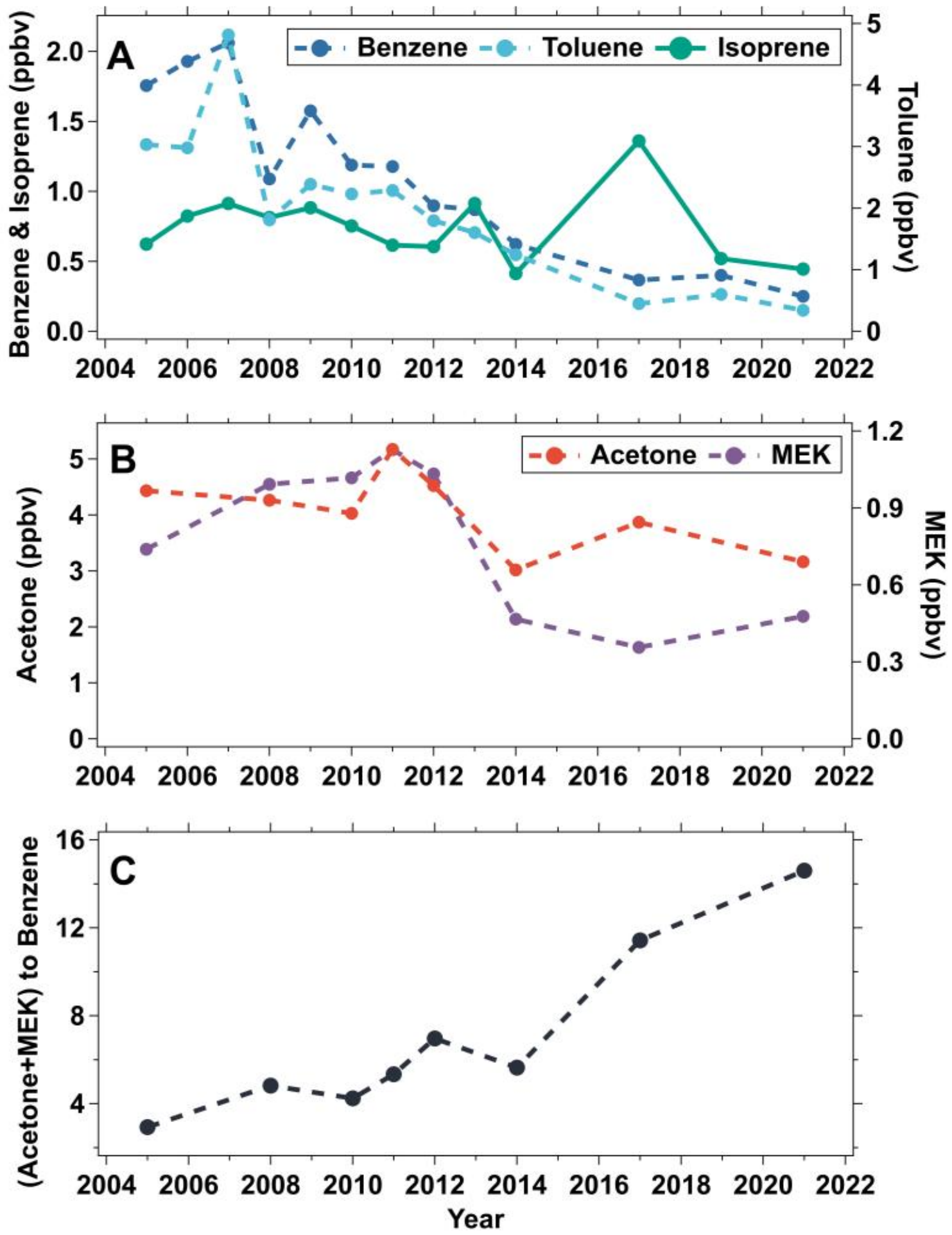

Fig. S8. Long-term trend of VOC species measured in urban Beijing during the summertime: (**A**) benzene, toluene, and isoprene, (**B**) acetone and MEK, (**C**) the concentration ratio of (acetone + MEK) to benzene. The averaged concentrations for the whole day are shown for the species except isoprene, which is the average concentration during 10:00-16:00.

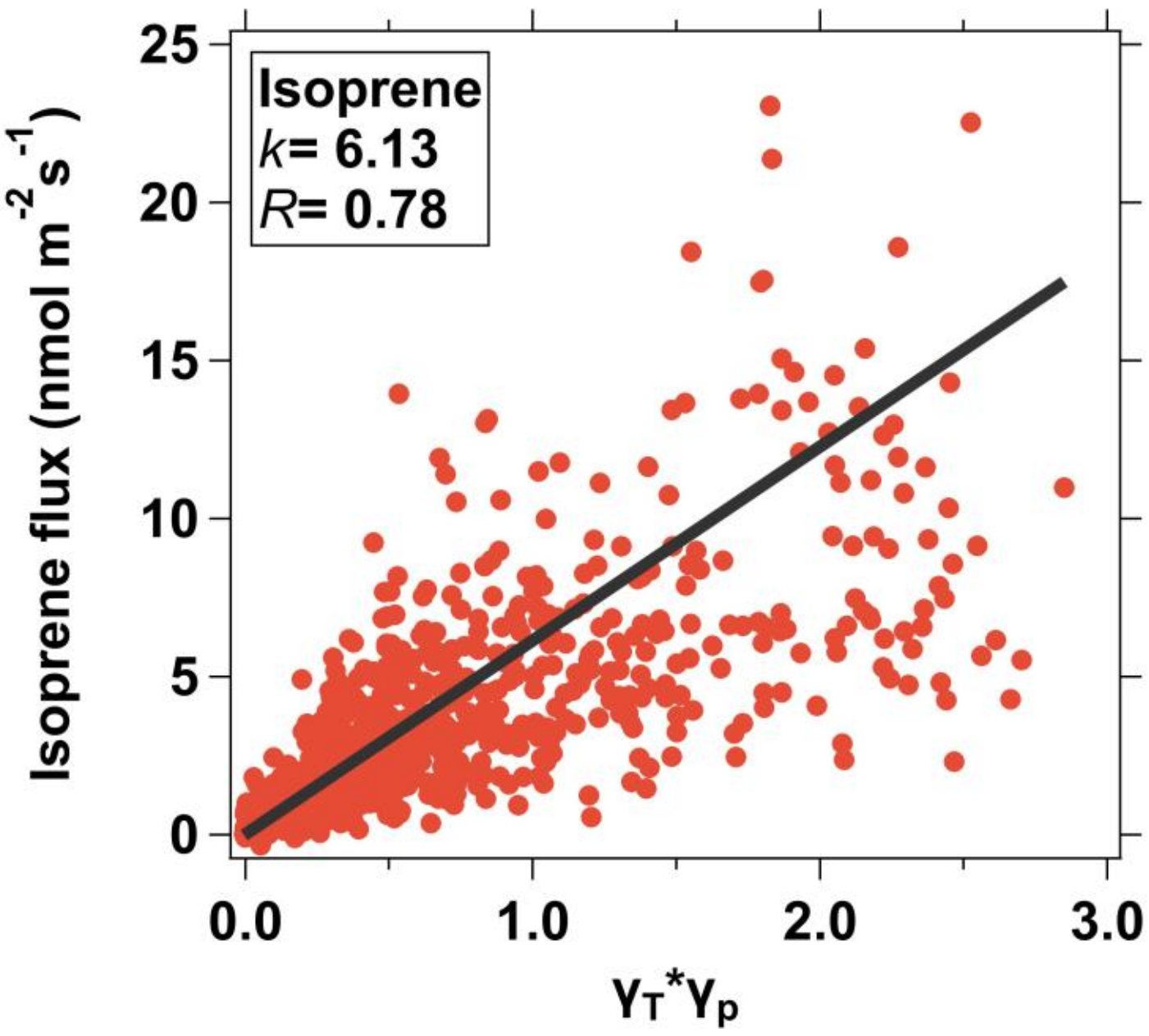

Fig. S9. Scatterplot of measured isoprene flux versus the product of the light activity parameters (*γ_P*) and temperature activity parameters (*γ_T*). The slope of the scatterplot represents the standardized isoprene flux (temperature at 30 °C and PAR at 1000 μmol m^{-2} s^{-1}).

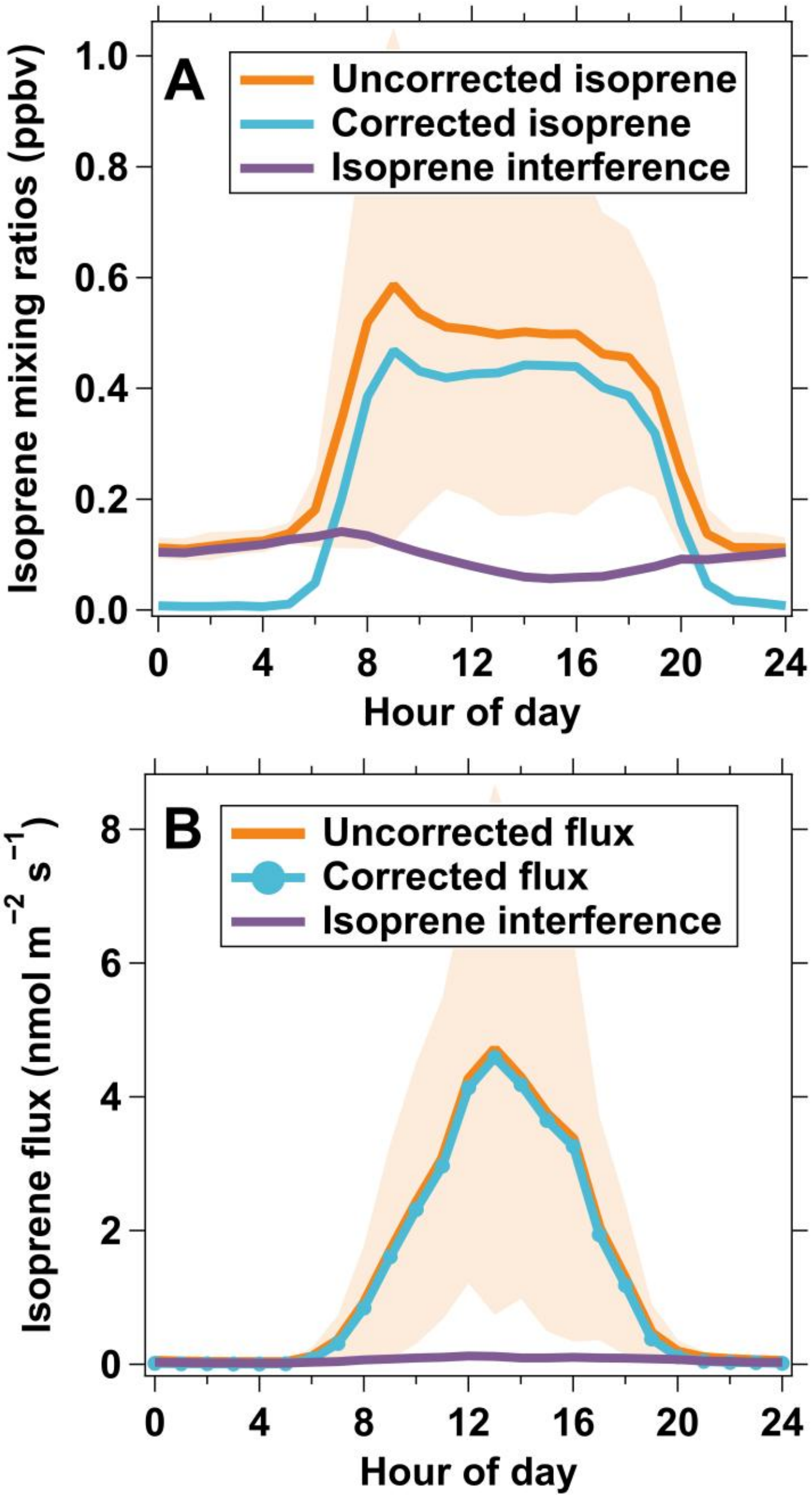

Fig. S10. Evaluation of isoprene interference on isoprene measurements by PTR-ToF during the campaign. Diurnal patterns of (**A**) concentrations and (**B**) fluxes for uncorrected isoprene, corrected isoprene, and isoprene interferences (Uncorrected - Corrected) over the entire campaign. The solid lines represent the mean values, and the shaded areas indicate the standard deviations of the corresponding isoprene concentrations or fluxes.

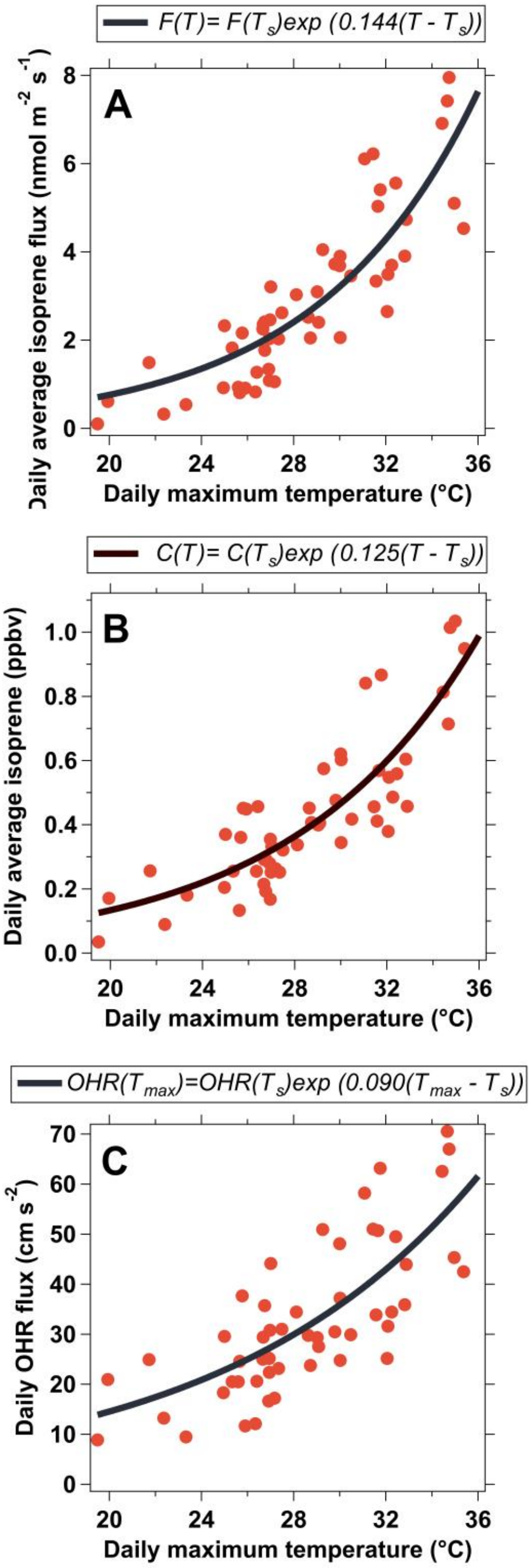

Fig. S11. Temperature dependence of (A) isoprene flux, (B) isoprene concentration and (C) total OHR_{flux}. Red data points in (A)-(C) indicate daytime daily means (08:00–18:00). The data were fitted using $Y(T) = Y(T_s) \times \exp[\beta (T - T_s)]$, where $Y(T_s)$ is the Y parameter at T_s (25 °C), β is the temperature response factor, and T is ambient temperature.

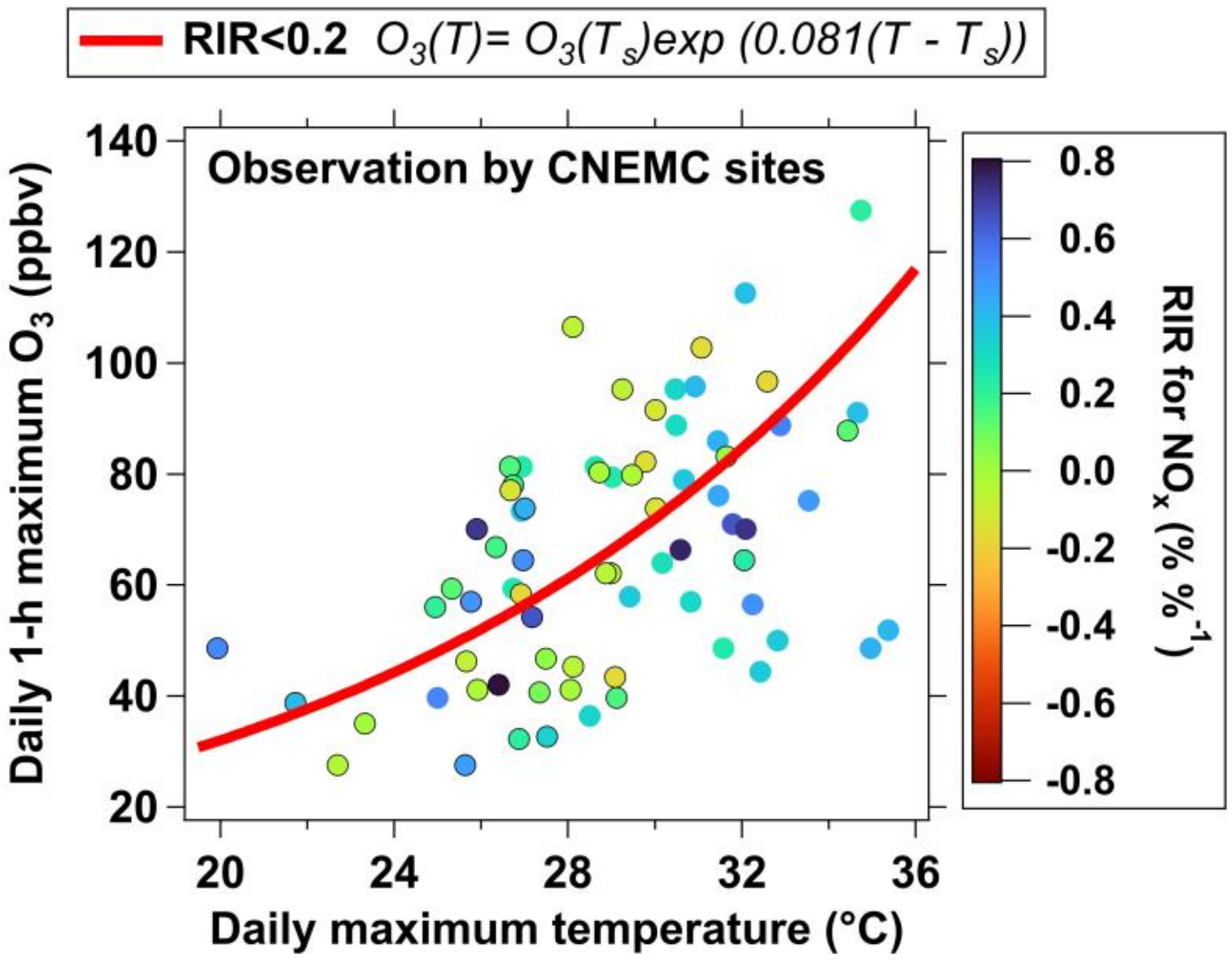

Fig. S12. Temperature dependence for ozone concentrations measured by the sites of the CNEMC network in Beijing. The averages of the daily 1-h maximum of ozone for the 12 CNEMC sites are determined and shown in this figure. The red line is fitted from the data points (with black outer circle) with RIR for NOx<0.2 % $\%^{-1}$.

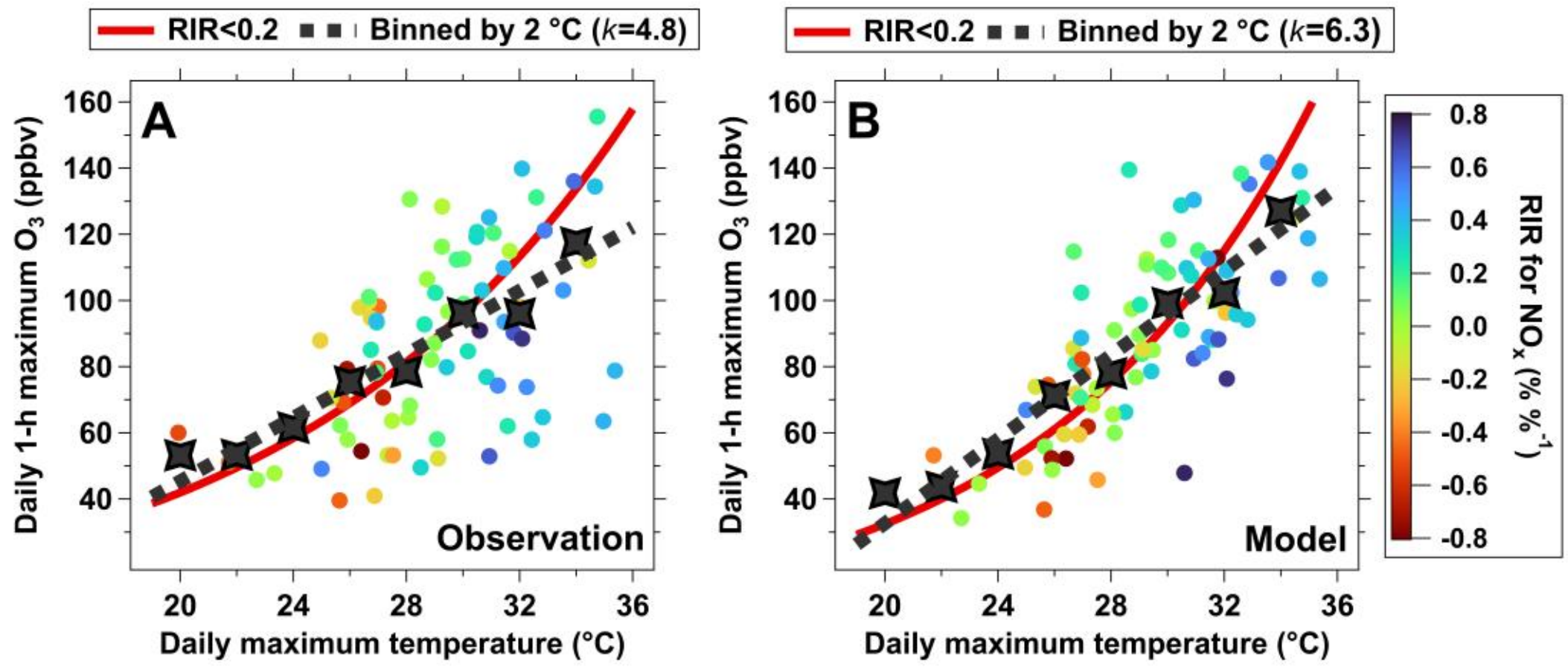

Fig. S13. Comparison of fitting methods for ozone temperature dependence. Temperature dependence for (**A**) observed ozone concentrations, and (**B**) modeled ozone concentrations. The red solid lines and the black dashed line represent the fit results obtained using the exponential curve in this study and the linear fit used in previous studies (*72*), respectively.

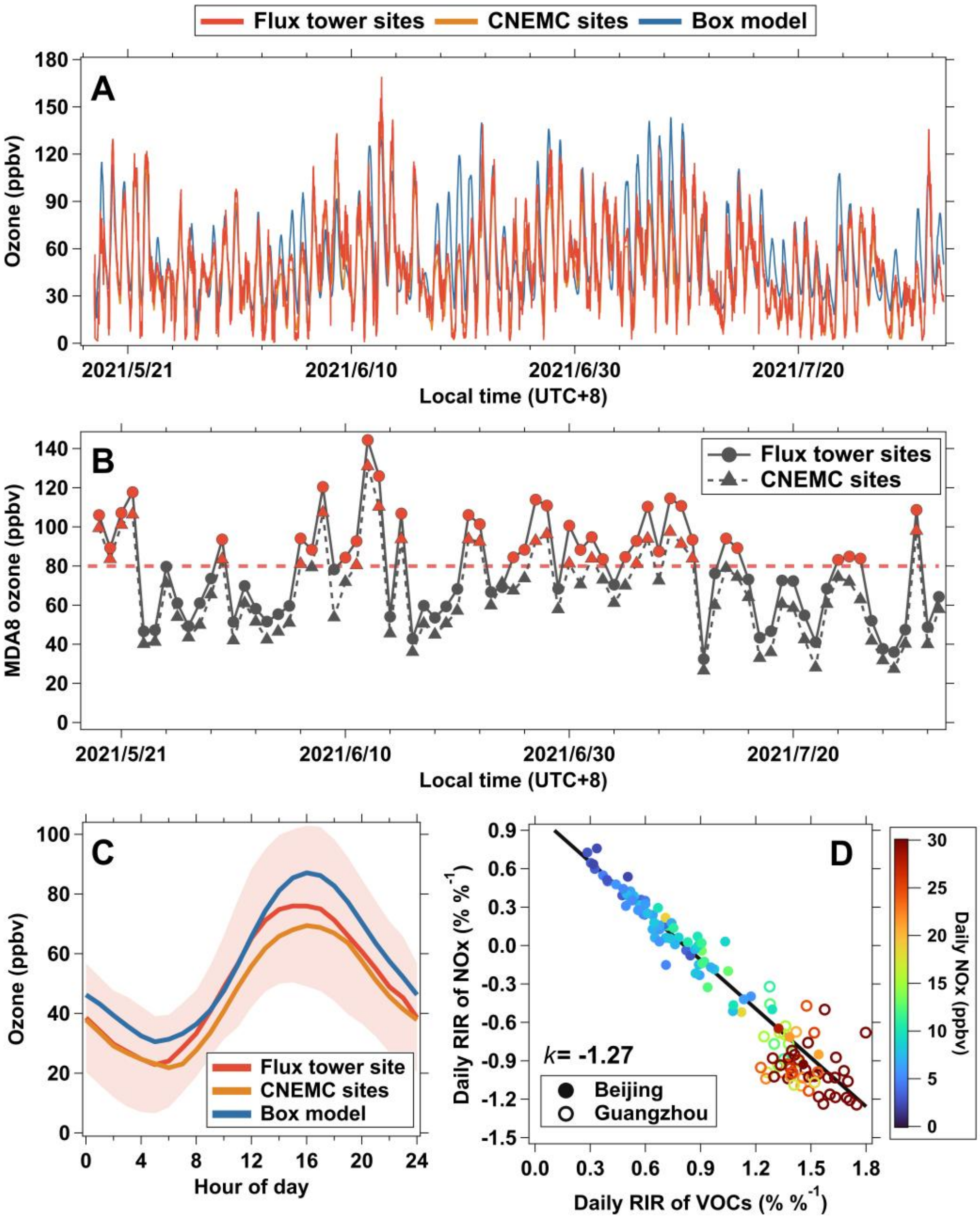

Fig. S14. Summary of measured and modeled ozone analysis for urban areas of Beijing and Guangzhou. (**A**) time series of ozone, (**B**) time series of maximum daily average 8-h (MDA8) ozone and (**C**) diurnal patterns at the flux tower site, the CNEMC sites, and modeled ozone from the box model. (**D**) Scatterplot of the daily average RIR of NOx versus the daily average RIR of VOCs from the box model for both Beijing and Guangzhou. The red data points shown in (B) represent the ozone-polluted day (MDA8 ozone >80 ppbv). The shaded area in (C) represents the standard deviations of observed ozone data from the flux tower site. The data points in (D) are color-coded using the daytime concentration of NOx averaged from the same time frame as the average of RIR.

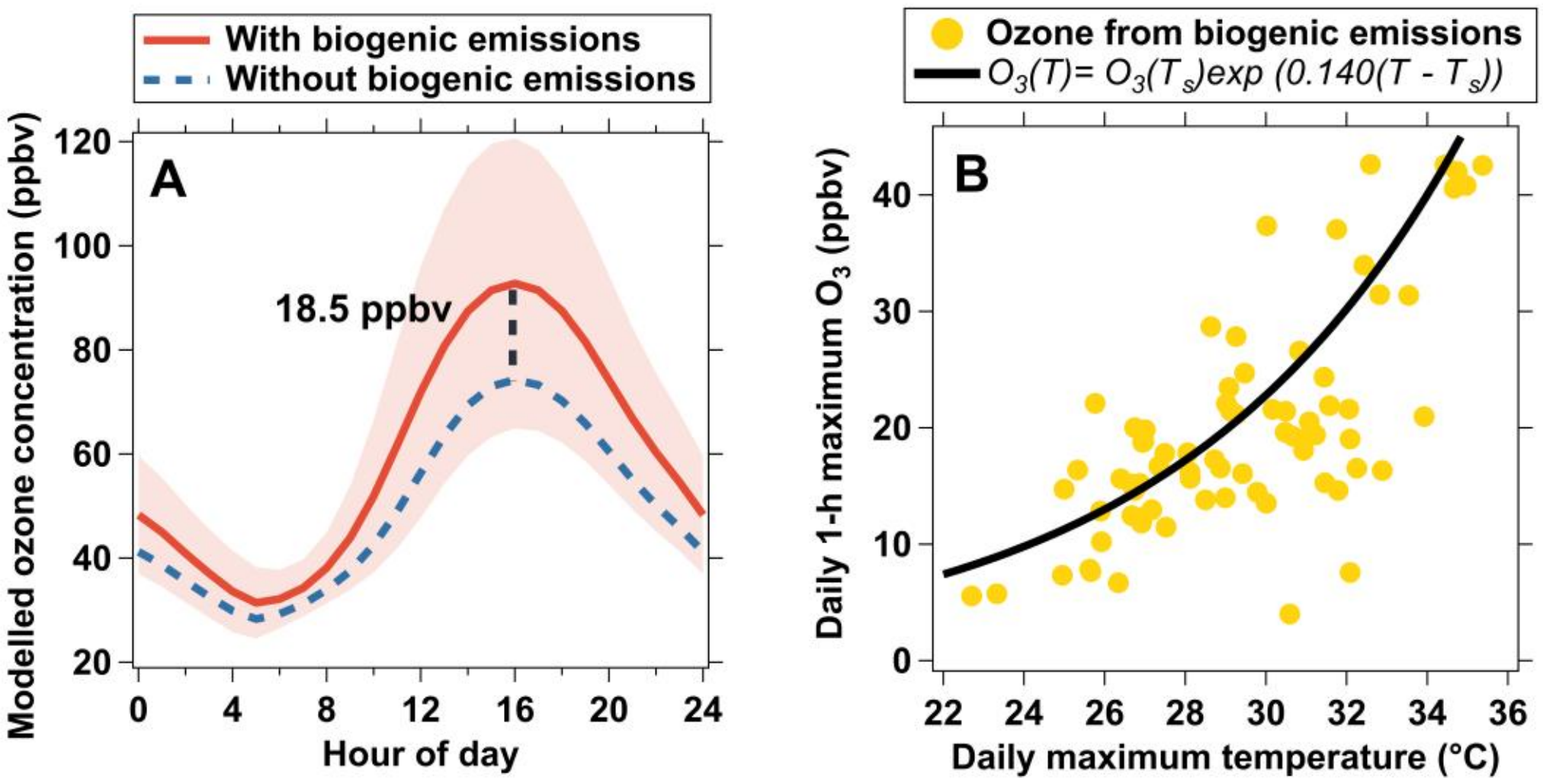

Fig. S15. Evaluating the impact of biogenic emissions on ozone formation in Beijing by box model simulations. (**A**) Diurnal patterns of modeled ozone concentration with and without biogenic emissions in model runs. (**B**) Temperature dependence for the daily 1-h maximum modeled ozone from biogenic emissions. The modeled ozone from biogenic emissions is determined by the difference between the modeled ozone with and without isoprene, monoterpenes and MVK&MACR contributed by biogenic emissions.

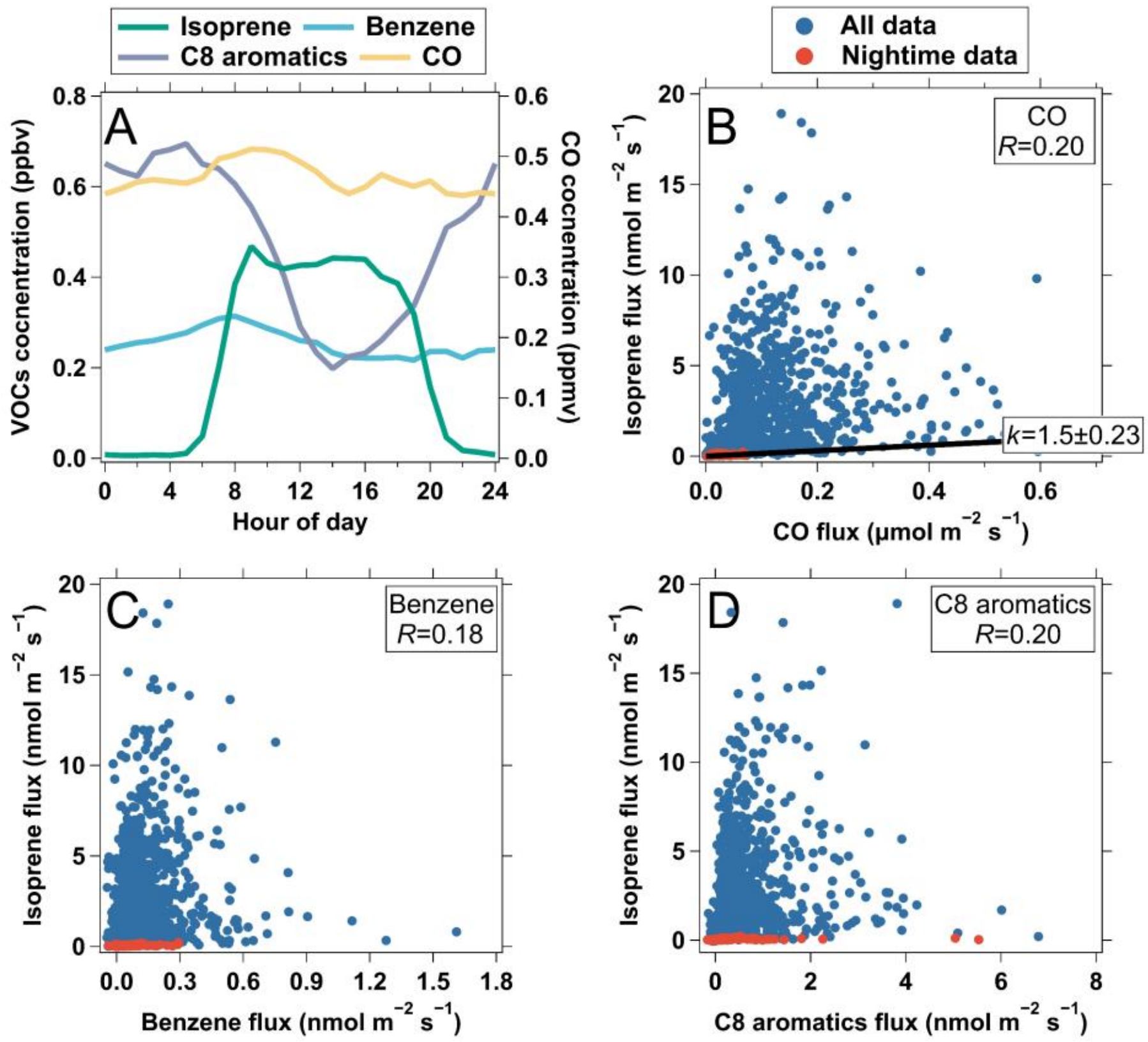

Fig. S16. **Diurnal variations of isoprene, benzene, C8 aromatics, and CO concentrations during the Beijing campaign.** (B–D) Scatterplots of isoprene flux versus CO, benzene and C8 aromatics flux. The black line in (B) shows the linear regression between isoprene and CO fluxes during the nighttime period (22:00–04:00). The regression slope represents the ratio of isoprene flux to CO flux from anthropogenic sources.

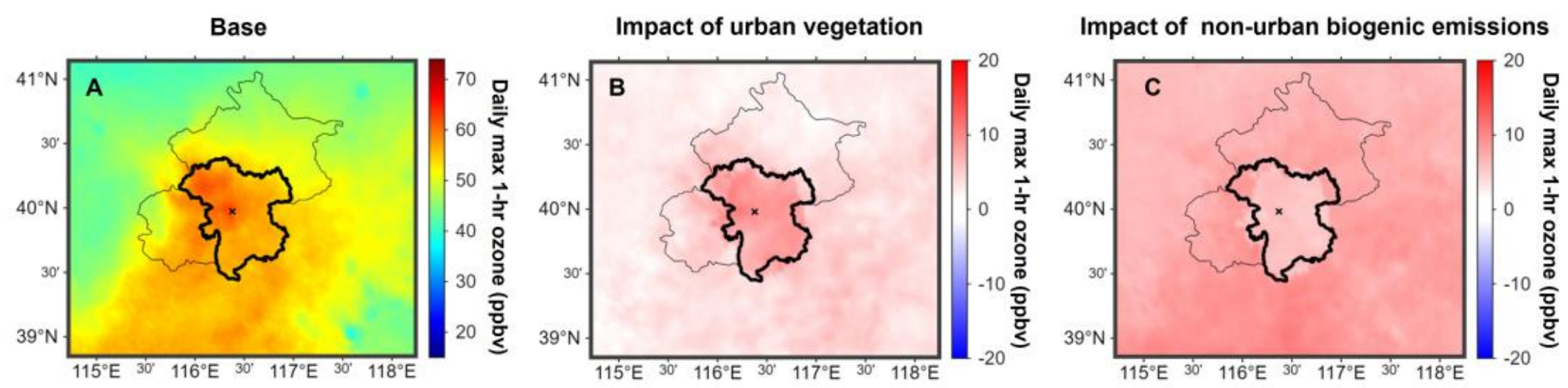

Fig. S17. Spatial distributions of WRF-Chem simulated average daily 1-h maximum ozone (ppbv) in Beijing and its surrounding areas from 20 May to 30 June 2021. (**A**) Simulated ozone concentrations in the Base scenario (all emissions included). (**B**) Impacts of urban vegetation emissions within the built-up area of Beijing on peak ozone enhancements. (**C**) Impact of non-urban biogenic emissions on peak ozone enhancements. The thin black outlines represent the administrative boundary of Beijing, while the thick black outlines delineate the urban built-up area used for evaluating urban vegetation emissions. The cross mark indicates the location of the flux measurement site.

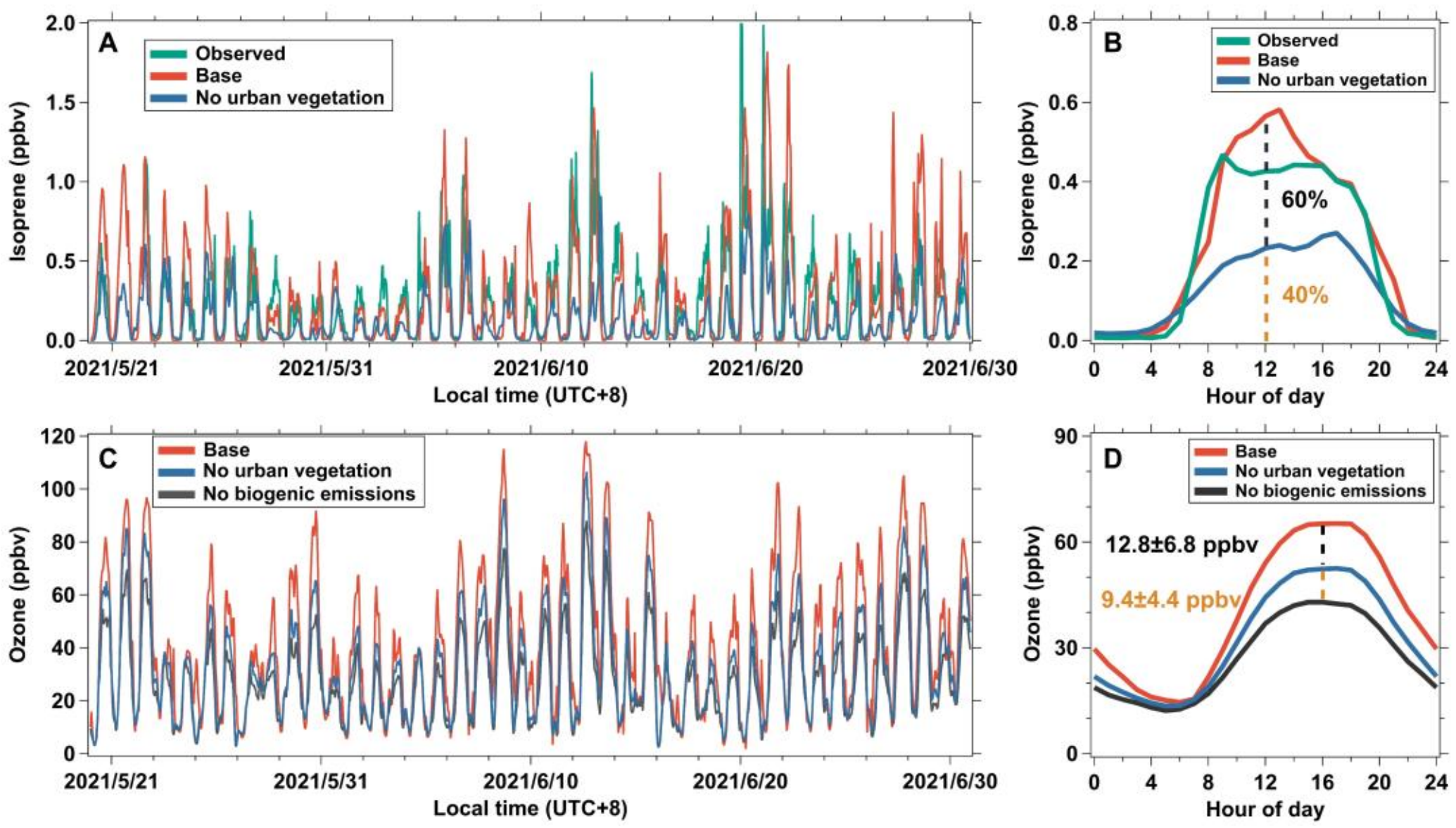

Fig. S18. WRF-Chem simulated isoprene and ozone concentrations in Beijing's built-up area from 20 May to 30 June 2021. (**A**) Time series and (**B**) diurnal patterns of isoprene from observations (at our measurement site) and WRF-Chem simulations. (**C**) Time series and (**D**) diurnal patterns of simulated ozone concentrations. The "Base", "No urban vegetation", and "No biogenic emissions" scenarios represent WRF-Chem runs with all emissions, without urban vegetation, and without total biogenic emissions, respectively.

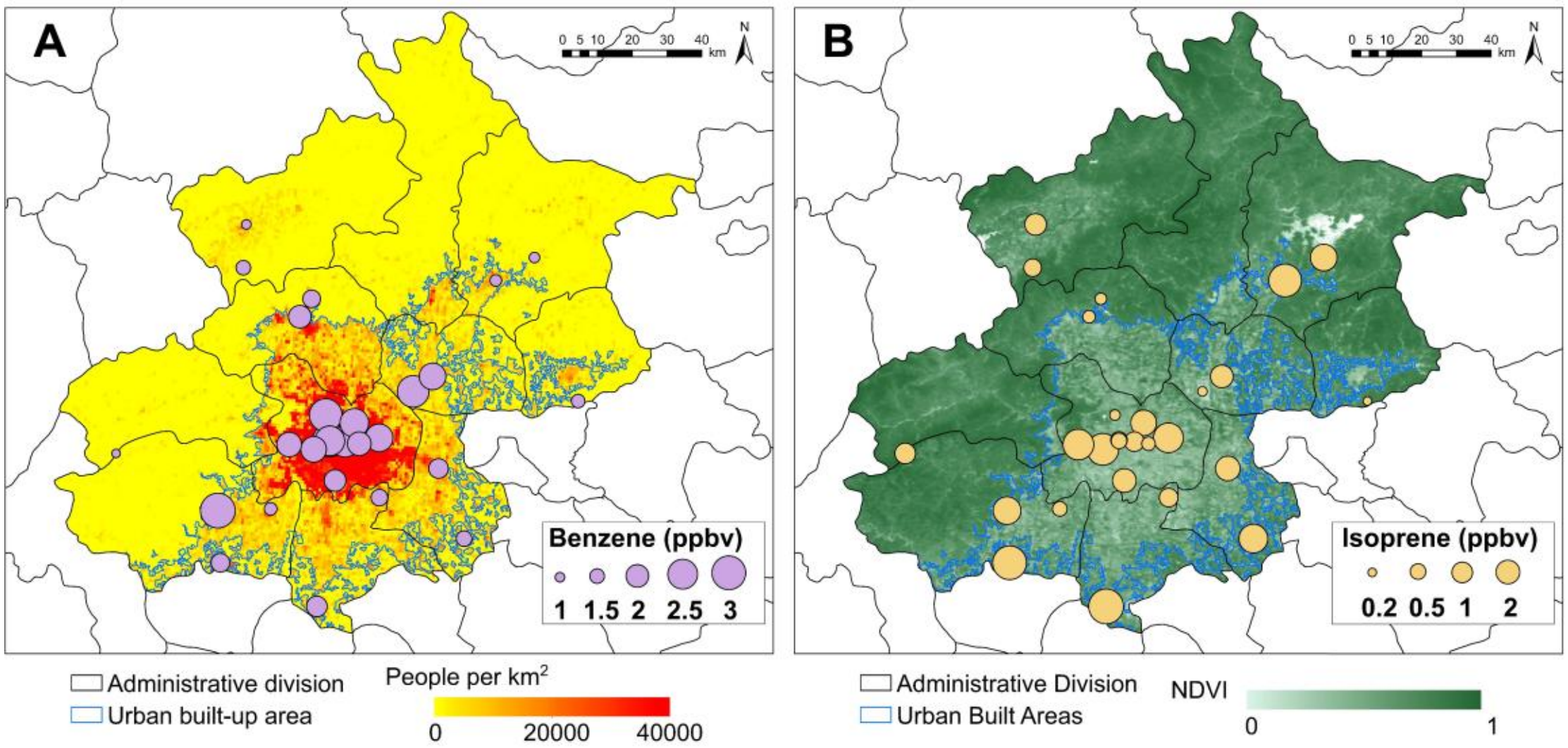

Fig. S19. Spatial variation of benzene and isoprene concentrations in Beijing. (**A**) Benzene and (**B**) isoprene were measured concurrently at 13:00 (LT) on a single day in July 2010. Population density and NDVI distribution in the administrative region of Beijing are shown. The urban built-up area is also shown in both plots. Isoprene concentrations are observed at similar magnitudes in city centers and rural areas. Benzene concentrations are largest in the city core, at the airport and in some industrial regions, and lowest in the rural areas.

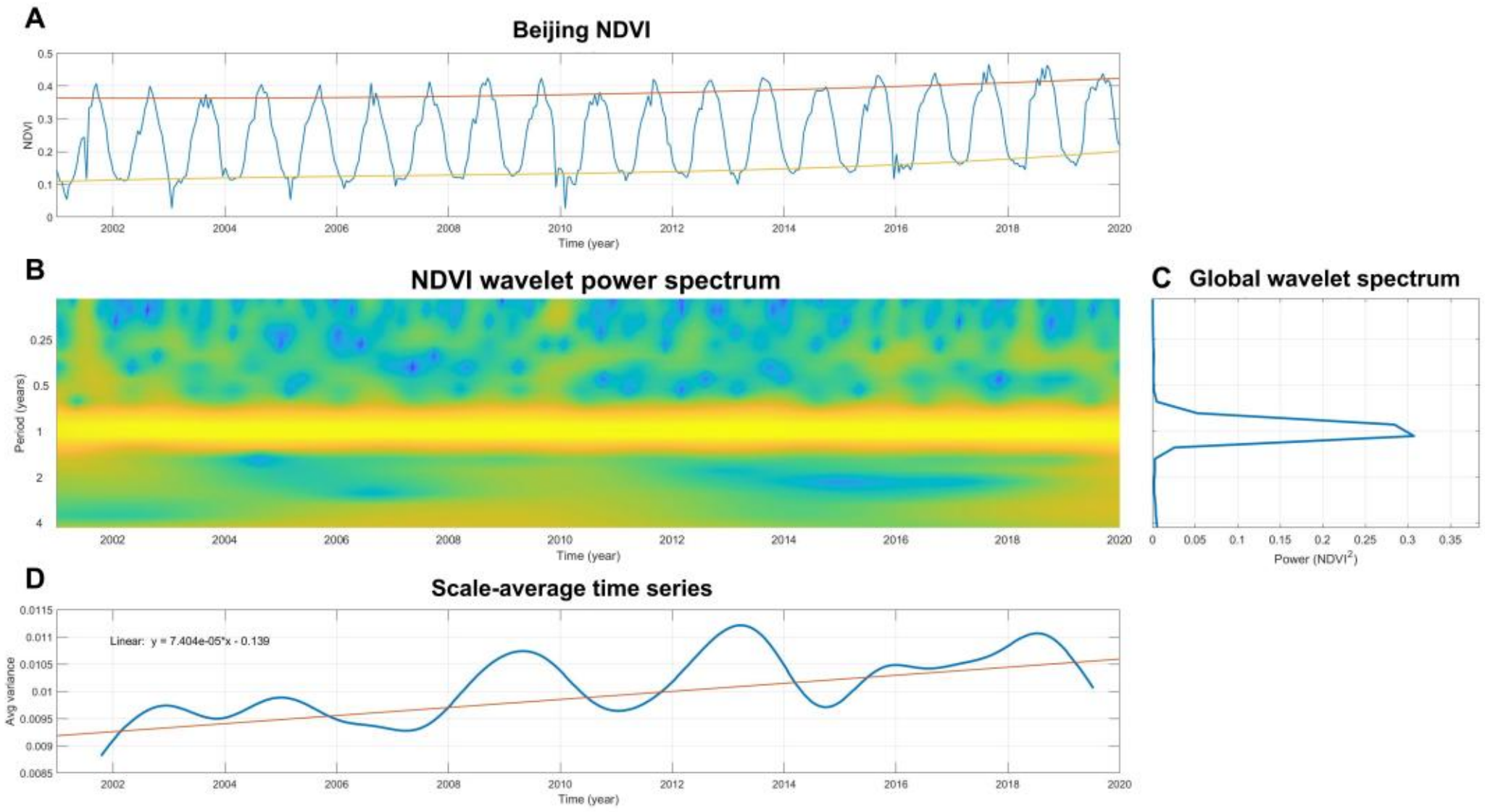

Fig. S20. Wavelet analysis of NDVI in Beijing in the past 20 years: (**A**) detrended NDVI (blue), winter (yellow) and summer (red) trend lines, (**B**) wavelet spectrum, (**C**) global wavelet spectrum, (**D**) yearly trend of NDVI. The solid lines in (A) display the annual NDVI trends for winter (yellow) and summer (red) in Beijing over the past 20 years, while (D) shows the overall annual NDVI trend for the same period.

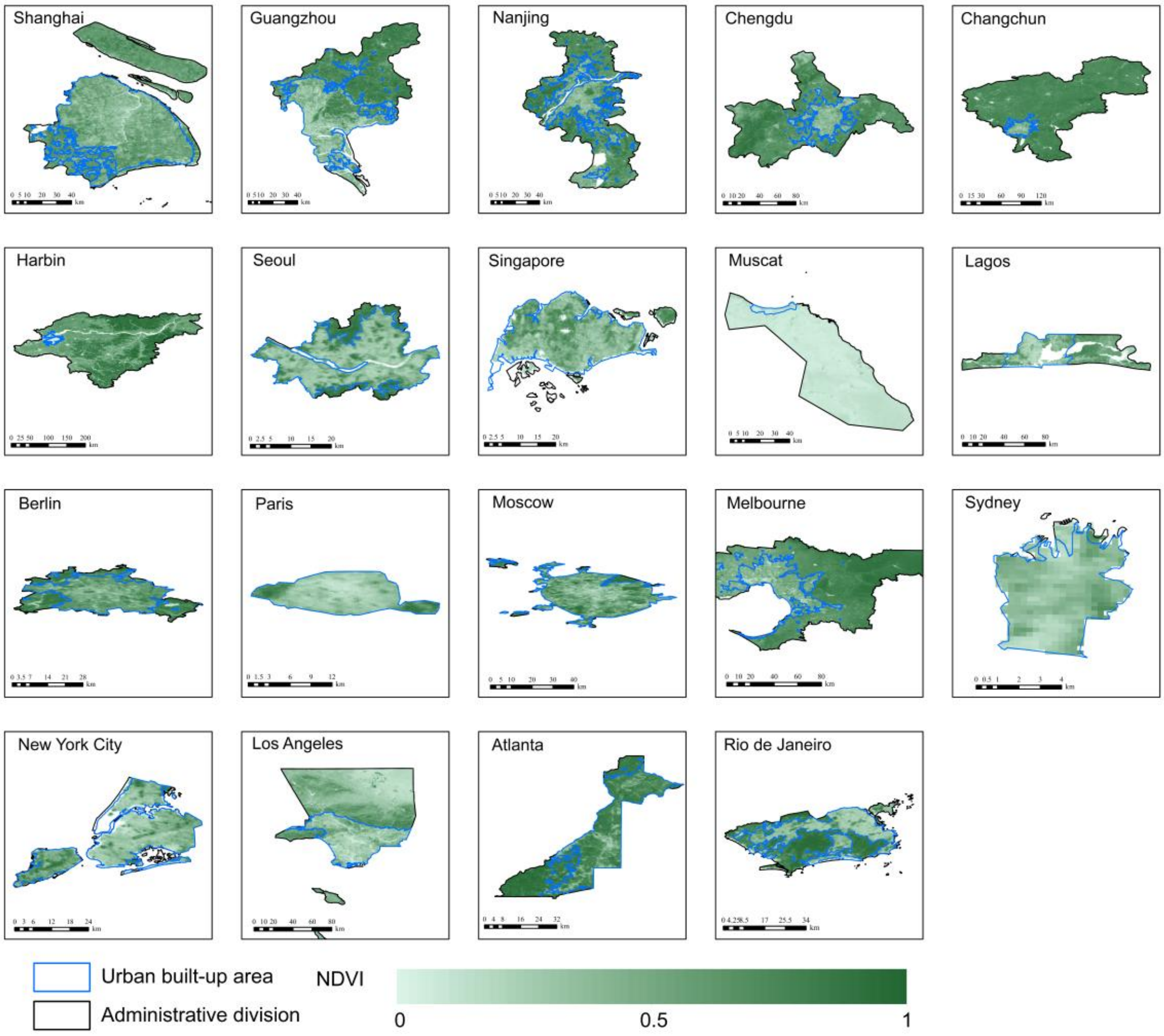

Fig. S21. The NDVI maps of the surveyed cities. The blue lines represent the boundary of urban built-up areas for each city.

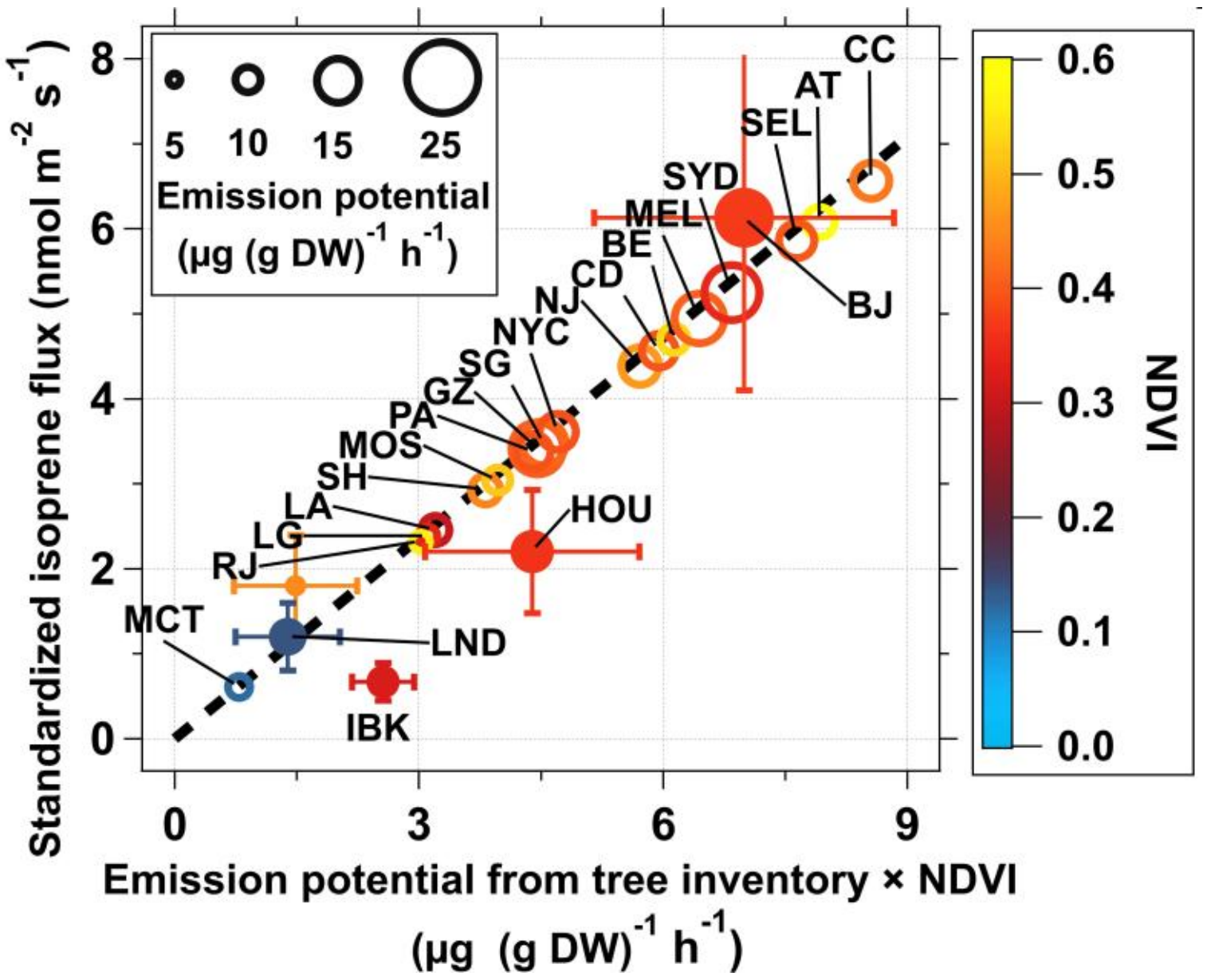

Fig. S22. Estimated standardized isoprene emissions of cities by normalized difference vegetation index (NDVI). Scatterplots show the observed standardized isoprene flux versus the products of average isoprene emission potential and NDVI for corresponding cities. The solid circles represent data points obtained from observations around the world (*74–77*), and the hollow circles represent the predicted results for other cities. The black dashed line shows the fitting results obtained using the weighted Orthogonal Distance Regression (ODR) method based on the solid circles. The symbol size is proportional to the average isoprene emission potential from the tree inventory of the corresponding cities.

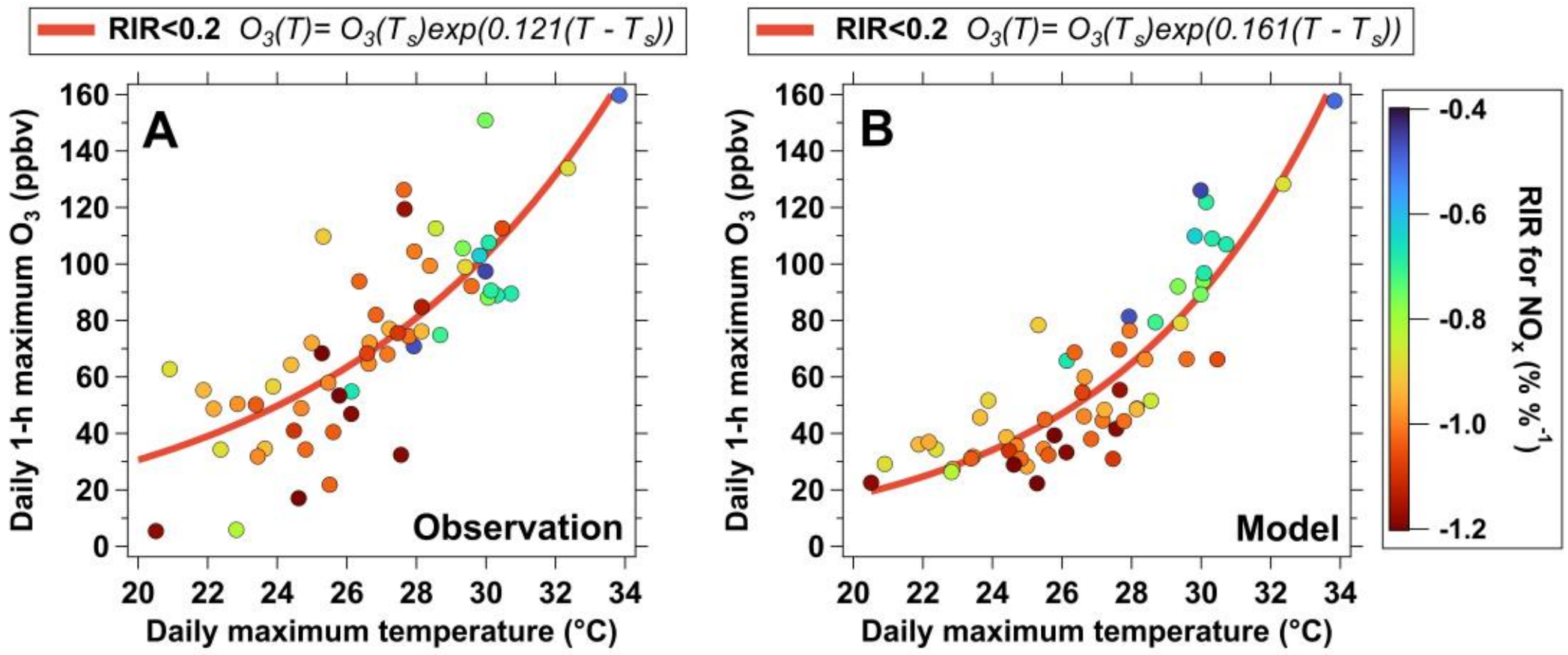

Fig. S23. Temperature dependence for (A) observed ozone concentrations, and (B) modeled ozone concentrations for the urban area of Guangzhou (*55*). The colored lines in (A) and (B) are fitted using $Y(T) = Y(T_s) \times \exp[\beta\ (T - T_s)]$ from ozone concentrations, where $Y(T_s)$ is the Y parameter at T_s (25 °C), β is the temperature response factor, and T is ambient temperature.

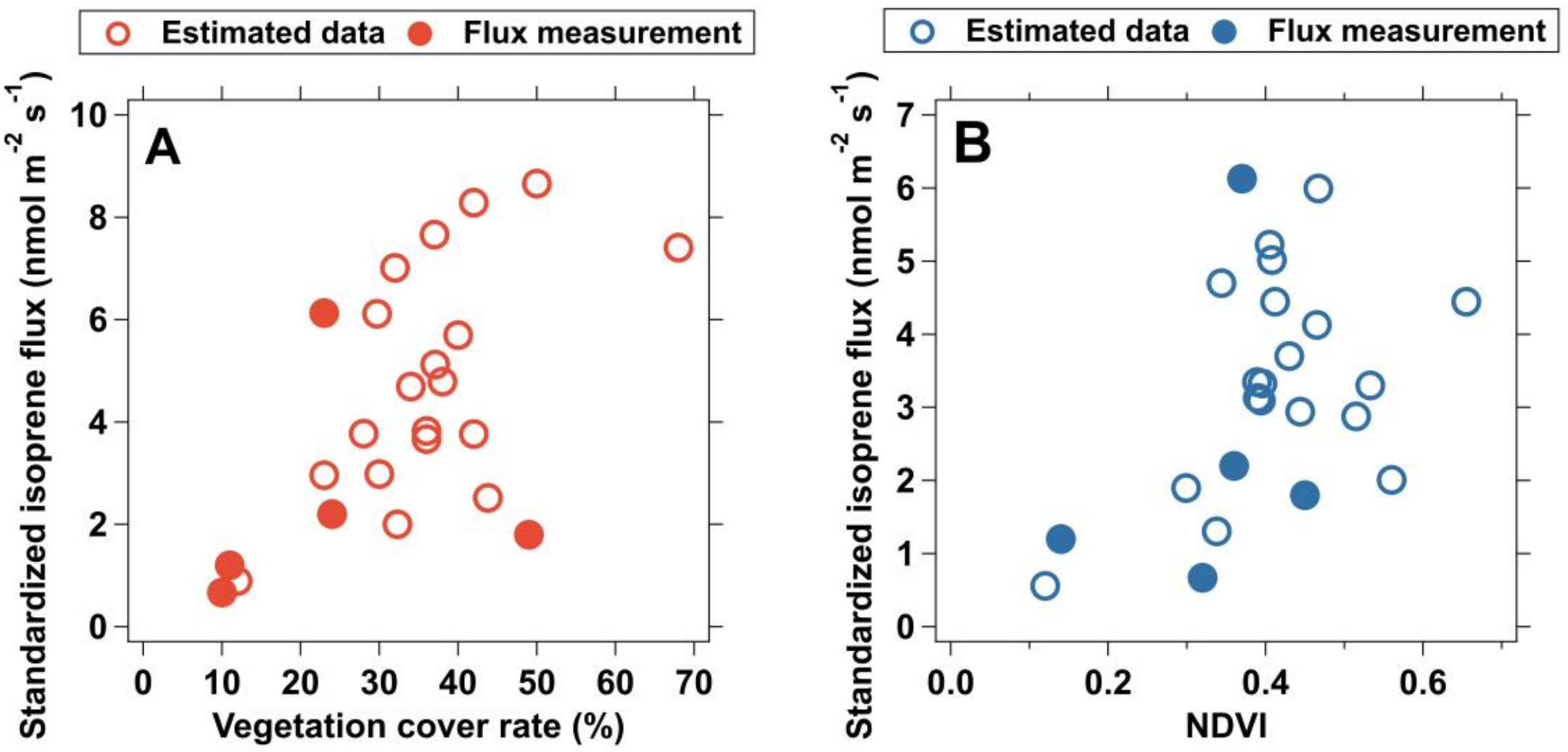

Fig. S24. Correlation analysis between vegetation cover rate, NDVI, and standardized isoprene flux. Scatterplots show the standardized isoprene flux (including estimated and measured results) versus (**A**) vegetation cover rate and (**B**) NDVI. The estimated standardized isoprene flux results shown in (A) and (B) were determined using vegetation cover rate and NDVI, respectively.

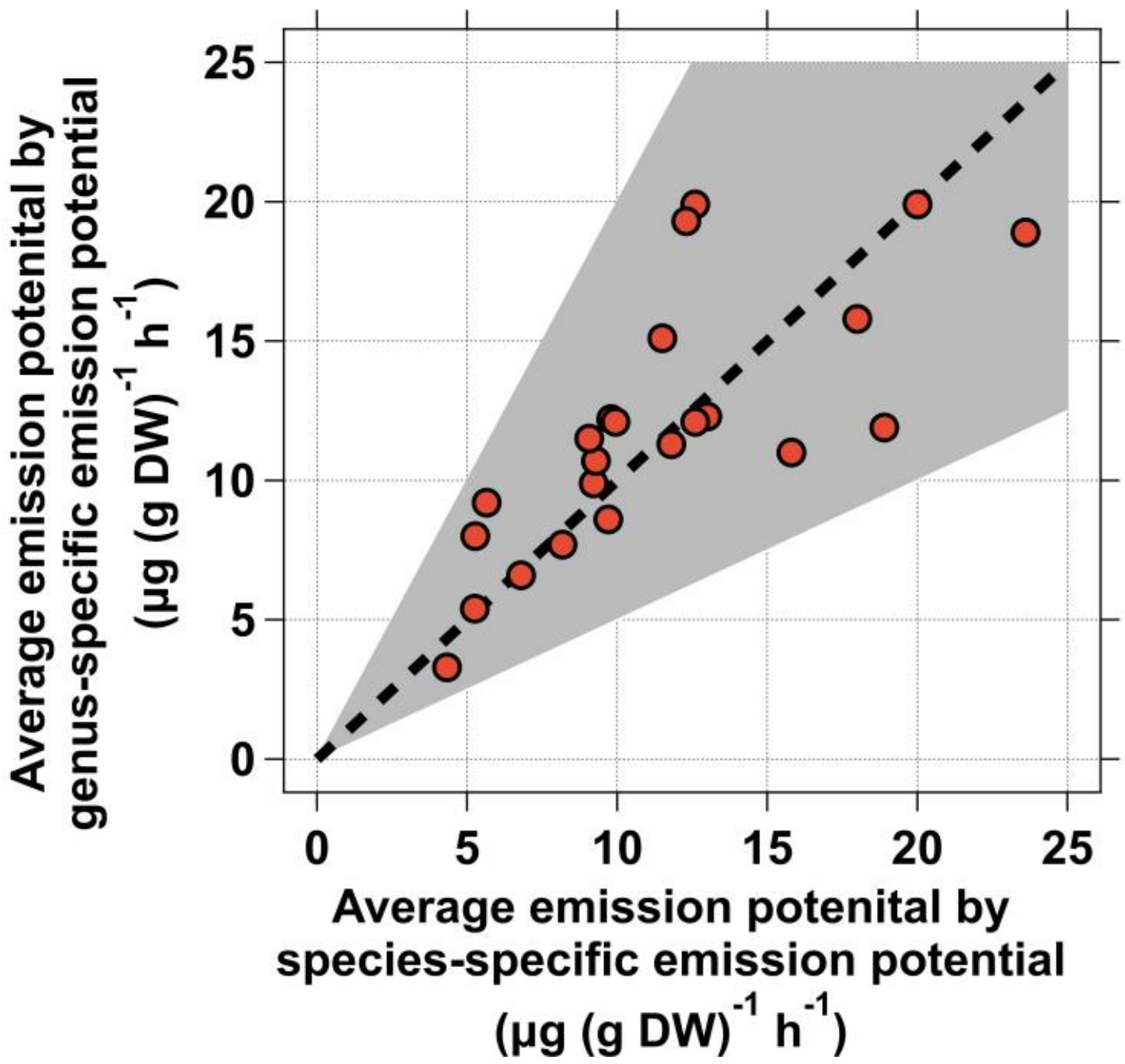

Fig. S25. Comparison of average emission potential estimates using species-specific and genus-specific emission potential data. The dashed black line denotes the 1:1 reference line and the gray shaded area indicates deviations within a factor of two.

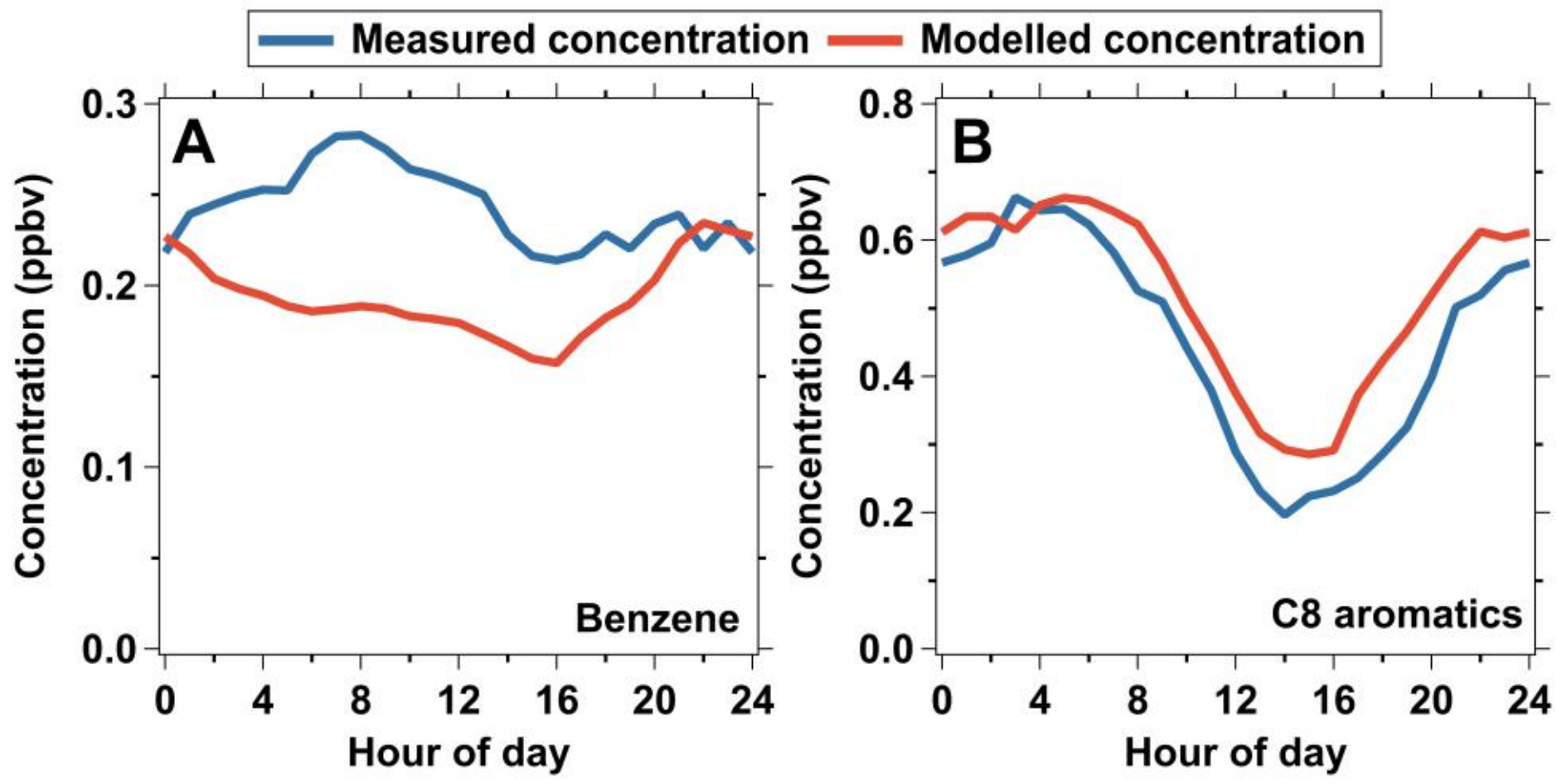

Fig. S26. Comparison of measured and simulated diurnal patterns of aromatic species. (**A**) Benzene and (**B**) C8 aromatics at a height of 102 m. Simulated concentrations were obtained by using a flux-driven box model as detailed in Text S6.

Table S1. Mass/charge ratio, ion formula and the identified VOC of the measured ions with detectable flux by PTR-ToF.

m/z	Ion formula	VOC identity	m/z	Ion formula	VOC identity
31.018	CH_2OH^+	Formaldehyde	106.078	$C_8H_{10}^+$	
33.033	CH_4OH^+	Methanol	107.034	$C_3H_6O_4H^+$	C3 4-oxy 1-DBE
41.039	$C_3H_5^+$	Propyne & fragments	107.070	$C_4H_{10}O_3H^+$	C4-acid water cluster
42.034	$C_2H_3NH^+$	Acetonitrile	107.107	$C_5H_{14}O_2H^+$	C5 alcohol water cluster
43.018	$C_2H_2OH^+$	Ethenone & fragments	107.049	$C_7H_6OH^+$	Benzaldehyde
43.054	$C_3H_6H^+$	Propene	107.086	$C_8H_{10}H^+$	C8 aromatics
45.033	$C_2H_4OH^+$	Acetaldehyde	108.102	$C_4H_{13}NO_2H^+$	C4 hydroxy amine water cluster
46.029	CH_3NOH^+	Formamide	108.081	$C_7H_9NH^+$	Toluidine
47.049	$C_2H_6OH^+$	Ethanol	109.101	$C_8H_{12}H^+$	Terpene fragment
48.044	CH_5NOH^+	Hydroxy methyl amine	111.080	$C_7H_{10}OH^+$	C3 furan
49.028	$CH_4O_2H^+$	Methanediol	111.117	$C_8H_{14}H^+$	C8 dienes
49.052	CH_6NOH^+		113.096	$C_7H_{12}OH^+$	Ethyl cyclopentanone
53.039	$C_4H_4H^+$	1-Buten-3-yne	113.132	$C_8H_{16}H^+$	Dimethyl cyclohexanes
54.034	$C_3H_3NH^+$	Acrylonitrile	115.075	$C_6H_{10}O_2H^+$	Dimethyl furanone
57.033	$C_3H_4OH^+$	Acrolein	115.112	$C_7H_{14}OH^+$	Heptanal

m/z	Ion formula	VOC identity	m/z	Ion formula	VOC identity
57.070	$C_4H_9^+$	Butenes & fragment	115.148	$C_8H_{18}H^+$	
58.065	$C_3H_7NH^+$	Propene amine	117.091	$C_6H_{12}O_2H^+$	Butyl ester acetic acid
59.049	$C_3H_6OH^+$	Acetone	117.127	$C_7H_{16}OH^+$	Butyl acetate
61.040	$CN_2H_4OH^+$		117.070	$C_9H_8H^+$	Indene
61.028	$C_2H_4O_2H^+$	Acetic acid	118.123	$C_6H_{15}NOH^+$	Hydroxy C6 amine
62.024	$CH_3NO_2H^+$	Nitromethane	119.070	$C_5H_{10}O_3H^+$	Diethyl carbonate
63.044	$C_2H_6O_2H^+$	1,2-ethanediol	119.107	$C_6H_{14}O_2H^+$	2-Butoxyethanol
63.026	$C_2H_6SH^+$	Dimethyl sulfide	119.089	$C_6H_{14}SH^+$	C6 thiols/sulfides
64.076	$C_2H_9NOH^+$	ethyl amine water cluster	119.086	$C_9H_{10}H^+$	Indane
65.039	$C_5H_4H^+$	Pentadiyne & fragments	120.066	$C_4H_9NO_3H^+$	C4 nitrates
67.054	$C_5H_6H^+$	1,3-Cyclopentadiene	120.102	$C_5H_{13}NO_2H^+$	C5 amide water cluster
68.026	$C_4H_3OH^+$		120.138	$C_6H_{17}NOH^+$	C6 amide water cluster
68.049	$C_4H_5NH^+$	Pyrrole & butene nitrile	120.081	$C_8H_9NH^+$	Dihydro pyridine
69.008	$C_2N_2OH^+$	Cyanogen isocyanate	121.086	$C_5H_{12}O_3H^+$	C5 acid water cluster
69.033	$C_4H_4OH^+$	Furan	121.122	$C_6H_{16}O_2H^+$	C6 alcohol water cluster
69.070	$C_5H_8H^+$	isoprene	121.101	$C_9H_{12}H^+$	C9 aromatics

m/z	Ion formula	VOC identity	m/z	Ion formula	VOC identity
70.065	$C_4H_7NH^+$	Butane nitrile	122.118	$C_5H_{15}NO_2H^+$	C5 hydroxy amine water cluster
71.049	$C_4H_6OH^+$	MVK & MACR	122.096	$C_8H_{11}NH^+$	C8 aromatic amines
71.086	$C_5H_{10}H^+$	Pentene & methyl butene	123.117	$C_9H_{14}H^+$	Santene & fragment
72.081	$C_4H_9NH^+$	Butene amine	125.132	$C_9H_{16}H^+$	trimethylcyclohex ane fragment
73.028	$C_3H_4O_2H^+$	Methyl glyoxal & acrylic acid	127.112	$C_8H_{14}OH^+$	6-MHO & cyclooctenone & (E)-2-octenal
73.065	$C_4H_8OH^+$	MEK	127.148	$C_9H_{18}H^+$	Trimethylcyclohe xane
75.044	$C_3H_6O_2H^+$	methyl acetate & hydroxyacetone	129.164	$C_9H_{20}H^+$	Naphthalene
75.080	$C_4H_{10}OH^+$	Butanols	133.086	$C_6H_{12}O_3H^+$	2-Butoxyethyl acetate
77.023	$C_2H_4O_3H^+$	Glycolic acid	133.122	$C_7H_{16}O_2H^+$	C7 saturated diols
78.055	$C_2H_7NO_2H^+$	C2 amide water cluster	133.076	$C_8H_8N_2H^+$	
79.063	$C_2H_9NO_2^+$	C3 amine water cluster	133.101	$C_{10}H_{12}H^+$	Tetrahydro naphthalene
79.075	$C_3H_{10}O_2H^+$	C2 hydroxy amine water cluster	134.118	$C_6H_{15}NO_2H^+$	C6 amide water cluster
79.054	$C_6H_6H^+$	Benzene	134.154	$C_7H_{19}NOH^+$	C7 amine water cluster
80.034	$CH_5NO_3H^+$	Trihydroxy methyl amine	134.096	$C_9H_{11}NH^+$	C9 1-nitro 5-DBE
81.055	$C_2H_8O_3H^+$	ethane diol water cluster	134.109	$C_{10}H_{14}^+$	C10 aromatics charge transfer

m/z	Ion formula	VOC identity	m/z	Ion formula	VOC identity
81.070	$C_6H_8H^+$		135.102	$C_6H_{14}O_3H^+$	C6 acid water cluster
82.065	$C_5H_7NH^+$	Methyl pyrrole	135.138	$C_7H_{18}O_2H^+$	C7 alcohol water cluster
82.078	$C_6H_9H^+$		135.117	$C_{10}H_{14}H^+$	C10 aromatics
83.060	$C_4H_6N_2H^+$	1-methyl pyrazole	136.112	$C_9H_{13}NH^+$	C9 aromatic amines
83.049	$C_5H_6OH^+$	Methylfuran	137.117	$C_6H_{16}O_3H^+$	C6 diol water cluster
83.086	$C_6H_{10}H^+$		137.107	$C_8H_{12}N_2H^+$	2,3,5,6-tetramethyl pyrazine
84.081	$C_5H_9NH^+$	Pentane nitriles	137.096	$C_9H_{12}OH^+$	4-propylphenol& dimethyl anisole
84.093	$C_6H_{11}H^+$	Cyclohexene	137.132	$C_{10}H_{16}H^+$	Monoterpenes
85.028	$C_4H_4O_2H^+$	2-(3H)Furanone	138.128	$C_9H_{15}NH^+$	C9 acrylonitrile
85.065	$C_5H_8OH^+$	C5 carbonyl	139.148	$C_{10}H_{18}H^+$	
85.101	$C_6H_{12}H^+$	2-Methyl-2-pentene	141.127	$C_9H_{16}OH^+$	Nonenal
86.096	$C_5H_{11}NH^+$	Piperidine & methyl pyrrolidine	141.164	$C_{10}H_{20}H^+$	
87.044	$C_4H_6O_2H^+$	Vinyl acetate	143.107	$C_8H_{14}O_2H^+$	Butyl methacrylate
87.080	$C_5H_{10}OH^+$	Pentanones	143.143	$C_9H_{18}OH^+$	Nonanal
88.076	$C_4H_9NOH^+$	C4 amides & C2 acetamides	143.179	$C_{10}H_{22}H^+$	Methyl naphthalene
89.060	$C_4H_8O_2H^+$	Ethyl acetate	146.081	$C_6H_{11}NO_3H^+$	C6-ene nitrates

m/z	Ion formula	VOC identity	m/z	Ion formula	VOC identity
89.096	$C_5H_{12}OH^+$	Pentanols	147.102	$C_7H_{14}O_3H^+$	ethyl 3-ethoxypropionate
90.091	$C_4H_{11}NOH^+$	Hydroxy C4 amines	147.138	$C_8H_{18}O_2H^+$	2-Ethyl-1,3-hexanediol
91.039	$C_3H_6O_3H^+$	Dimethyl carbonate	147.120	$C_8H_{18}SH^+$	C8 thiols/sulfides
91.075	$C_4H_{10}O_2H^+$	Butane diols	147.117	$C_{11}H_{14}H^+$	4-isopropyl styrene
91.054	$C_7H_6H^+$	Aromatic fragments	149.117	$C_7H_{16}O_3H^+$	C7 acid water cluster
92.034	$C_2H_5NO_3H^+$	Ethyl nitrate	149.154	$C_8H_{20}O_2H^+$	C8 alcohol water cluster
92.071	$C_3H_9NO_2H^+$	C3 amide water cluster	149.132	$C_{11}H_{16}H^+$	C11 aromatics
92.107	$C_4H_{13}NOH^+$	C4 amine water cluster	151.148	$C_{11}H_{18}H^+$	
93.055	$C_3H_8O_3H^+$	propanoic acid water cluster	153.127	$C_{10}H_{16}OH^+$	Camphor
93.091	$C_4H_{12}O_2H^+$	C4 alcohol water cluster	153.164	$C_{11}H_{20}H^+$	Acenaphtylene
93.070	$C_7H_8H^+$	Toluene	155.179	$C_{11}H_{22}H^+$	
94.086	$C_3H_{11}NO_2H^+$	C3 hydroxy amine water cluster	157.159	$C_{10}H_{20}OH^+$	Decanal
94.065	$C_6H_7NH^+$	2-methyl pyridine	161.154	$C_9H_{20}O_2H^+$	C9 saturated diols
95.086	$C_7H_{10}H^+$		161.136	$C_9H_{20}SH^+$	C9 thiols/sulfides
96.081	$C_6H_9NH^+$	1-ethyl pyrrole	165.149	$C_8H_{20}O_3H^+$	C8 diol water cluster
96.093	$C_7H_{11}H^+$	/	165.164	$C_{12}H_{20}H^+$	

m/z	Ion formula	VOC identity	m/z	Ion formula	VOC identity
97.050	$C_2H_8O_4H^+$	acetic acid water cluster	166.172	$C_{12}H_{21}H^+$	
97.028	$C_5H_4O_2H^+$	Furfurals	169.195	$C_{12}H_{24}H^+$	
97.011	$C_5H_4SH^+$	cyclopentadiene sulfone	179.164	$C_9H_{22}O_3H^+$	C9 diol water cluster
97.065	$C_6H_8OH^+$	Dimethyl furan	179.179	$C_{13}H_{22}H^+$	
97.101	$C_7H_{12}H^+$	Methylcyclohexene & fragment	181.195	$C_{13}H_{24}H^+$	
98.096	$C_6H_{11}NH^+$	4-methyl pentanenitrile	183.211	$C_{13}H_{26}H^+$	
99.044	$C_5H_6O_2H^+$	2-furanmethanol	193.159	$C_{13}H_{20}OH^+$	Damascone
99.080	$C_6H_{10}OH^+$	cyclohexanone	193.195	$C_{14}H_{24}H^+$	
101.060	$C_5H_8O_2H^+$	Methyl methacrylate	205.195	$C_{15}H_{24}H^+$	Sesquiterpenes
101.096	$C_6H_{12}OH^+$	C6 Carbonyls	207.174	$C_{14}H_{22}OH^+$	Norpatchoulenol
101.132	$C_7H_{16}H^+$	Heptanes	207.211	$C_{15}H_{26}H^+$	
102.091	$C_5H_{11}NOH^+$	Nitro C4 alkenes	211.206	$C_{14}H_{26}OH^+$	Sandalore
102.128	$C_6H_{15}NH^+$	C6 amines	211.242	$C_{15}H_{30}H^+$	
103.039	$C_4H_6O_3H^+$	acetic anhydride	219.211	$C_{16}H_{26}H^+$	Perhydropyrene
103.075	$C_5H_{10}O_2H^+$	Propyl acetate	223.064	$C_6H_{18}O_3Si_3H^+$	D3 siloxane
103.054	$C_8H_6H^+$	Ethynyl benzene	223.242	$C_{16}H_{30}H^+$	
105.055	$C_4H_8O_3H^+$	hydroxy or peroxy butanoic acid	225.258	$C_{16}H_{32}H^+$	

m/z	Ion formula	VOC identity	m/z	Ion formula	VOC identity
105.091	$C_5H_{12}O_2H^+$	pentane diols	297.082	$C_8H_{24}O_4Si_4H^+$	D4 siloxane
105.070	$C_8H_8H^+$	Styrene	371.101	$C_{10}H_{30}O_5Si_5H^+$	D5 siloxane
106.086	$C_4H_{11}NO_2H^+$	C4 dihydroxy amine	445.120	$C_{12}H_{36}O_6Si_6H^+$	D6 siloxane
106.123	$C_5H_{15}NOH^+$	C4 amine water cluster			
106.065	$C_7H_7NH^+$	Vinyl pyridine			

Table S2. The ratio (*R*) of the sum of alkanes, ethene and acetylene to the sum of OVOCs and aromatics for domestic VCP emissions and vehicle exhausts measured by Wang et al. (*91*).

Emission source	*R* = (alkanes + ethene + acetylene)/(OVOCs + aromtics)
Coatings	0.01
Adhesives	0.01
Printing inks	0.01
Cleaning products	0.11
Personal care products	0.31
Pesticides	2.34
Non-industrial VCP	0.14
Gasoline vehicle exhaust	2.19

Table S3. Rate constants with OH radical of the measured VOCs. The OH rate constants presented in the table are measured at a temperature of 298 K.

m/z	Ion formula	OH rate constant (10^{-12} cm^3 molecule^{-1} s^{-1})	References
31.018	CH_2OH^+	9.4	Atkinson et al. (*29*)
33.033	CH_4OH^+	0.8	Atkinson et al. (*29*)
41.039	$C_3H_5^+$	0.7	Atkinson et al. (*29*)
42.034	$C_2H_3NH^+$	0.02	Gilman et al. (*128*)
43.054	$C_3H_6H^+$	29.0	Atkinson et al. (*29*)
45.033	$C_2H_4OH^+$	15.0	Atkinson et al. (*29*)
46.029	CH_3NOH^+	1.5	NIST database (*129*)
47.049	$C_2H_6OH^+$	3.2	Atkinson et al. (*29*)
49.028	$CH_4O_2H^+$	7.0	NIST database (*129*)
53.039	$C_4H_4H^+$	20.0	Atkinson et al. (*29*)
54.034	$C_3H_3NH^+$	4.0	Gilman et al. (*128*)
57.033	$C_3H_4OH^+$	20.0	Gilman et al. (*128*)
57.070	$C_4H_9^+$	31.0	Atkinson et al. (*29*)
58.065	$C_3H_7NH^+$	15.0	Koss et al. (*130*)
59.049	$C_3H_6OH^+$	0.2	Atkinson et al. (*29*)
61.028	$C_2H_4O_2H^+$	3.7	NIST database (*129*)
62.024	$CH_3NO_2H^+$	0.3	Gilman et al. (*128*)
63.044	$C_2H_6O_2H^+$	14.5	Pfannerstill et al. (*131*)
63.026	$C_2H_6SH^+$	6.0	NIST database (*129*)
67.054	$C_5H_6H^+$	92.0	Gilman et al. (*128*)
68.049	$C_4H_5NH^+$	111.4	Gilman et al. (*128*)
69.033	$C_4H_4OH^+$	40.0	Gilman et al. (*128*)

m/z	Ion formula	OH rate constant (10^{-12} cm^3 $molecule^{-1}$ s^{-1})	References
69.070	$C_5H_8H^+$	100.0	Atkinson et al. (*29*)
70.065	$C_4H_7NH^+$	7.7	Koss et al. (*130*)
71.049	$C_4H_6OH^+$	24.8	Atkinson et al. (*29*)
71.086	$C_5H_{10}H^+$	31.4	Atkinson et al. (*29*)
72.081	$C_4H_9NH^+$	25.0	Koss et al. (*130*)
73.028	$C_3H_4O_2H^+$	21.1	Koss et al. (*130*)
73.065	$C_4H_8OH^+$	5.5	Koss et al. (*130*)
75.044	$C_3H_6O_2H^+$	0.3	NIST database (*129*)
75.080	$C_4H_{10}OH^+$	8.5	Gilman et al. (*128*)
77.023	$C_2H_4O_3H^+$	12.1	Gilman et al. (*128*)
79.054	$C_6H_6H^+$	1.2	Atkinson et al. (*29*)
82.065	$C_5H_7NH^+$	62.7	Gilman et al. (*128*)
83.049	$C_5H_6OH^+$	90.6	Liljegren et al. (*132*)
83.086	$C_6H_{10}H^+$	86.9	Wang et al. (*94*)
84.081	$C_5H_9NH^+$	0.5	Koss et al. (*130*)
85.028	$C_4H_4O_2H^+$	44.5	Gilman et al. (*128*)
85.065	$C_5H_8OH^+$	11.5	NIST database (*129*)
85.101	$C_6H_{12}H^+$	66.3	Wang et al. (*94*)
87.044	$C_4H_6O_2H^+$	12.8	Wang et al. (*94*)
87.080	$C_5H_{10}OH^+$	7.9	Atkinson et al. (*29*)
89.060	$C_4H_8O_2H^+$	1.8	Koss et al. (*130*)
89.096	$C_5H_{12}OH^+$	16.4	Wang et al. (*94*)
91.039	$C_3H_6O_3H^+$	0.3	Wang et al. (*94*)

m/z	Ion formula	OH rate constant (10^{-12} cm^3 $molecule^{-1}$ s^{-1})	References
91.075	$C_4H_{10}O_2H^+$	36.7	Wang et al. (*94*)
93.070	$C_7H_8H^+$	5.6	Atkinson et al. (*29*)
94.065	$C_6H_7NH^+$	2.6	Wu et al. (*55*)
95.086	$C_7H_{10}H^+$	46.8	Pfannerstill et al. (*131*)
96.081	$C_6H_9NH^+$	145.0	Koss et al. (*130*)
97.028	$C_5H_4O_2H^+$	35.6	Gilman et al. (*128*)
97.065	$C_6H_8OH^+$	132.0	Wu et al. (*55*)
97.101	$C_7H_{12}H^+$	79.8	NIST database (*129*)
98.096	$C_6H_{11}NH^+$	5.0	Koss et al. (*130*)
99.044	$C_5H_6O_2H^+$	13.6	Koss et al. (*130*)
99.080	$C_6H_{10}OH^+$	5.4	Wu et al. (*55*)
101.060	$C_5H_8O_2H^+$	82.6	Wang et al. (*94*)
101.096	$C_6H_{12}OH^+$	18.6	Koss et al. (*130*)
101.132	$C_7H_{16}H^+$	6.8	Pfannerstill et al. (*131*)
103.039	$C_4H_6O_3H^+$	43.0	Koss et al. (*130*)
103.075	$C_5H_{10}O_2H^+$	3.5	Wang et al. (*94*)
103.054	$C_8H_6H^+$	1.0	Gilman et al. (*128*)
105.070	$C_8H_8H^+$	58.0	Atkinson et al. (*29*)
106.065	$C_7H_7NH^+$	57.0	NIST database (*129*)
107.070	$C_4H_{10}O_3H^+$	6.0	Gilman et al. (*128*)
107.049	$C_7H_6OH^+$	12.0	Atkinson et al. (*29*)
107.086	$C_8H_{10}H^+$	13.2	Wu et al. (*55*)
108.081	$C_7H_9NH^+$	3.2	NIST database (*129*)

m/z	Ion formula	OH rate constant (10^{-12} cm^3 $molecule^{-1}$ s^{-1})	References
109.101	$C_8H_{12}H^+$	107.0	Pfannerstill et al. (*131*)
111.080	$C_7H_{10}OH^+$	23.3	Koss et al. (*130*)
111.117	$C_8H_{14}H^+$	120.0	Pfannerstill et al. (*131*)
113.096	$C_7H_{12}OH^+$	10.0	NIST database (*129*)
115.075	$C_6H_{10}O_2H^+$	20.0	Koss et al. (*130*)
115.112	$C_7H_{14}OH^+$	21.4	Atkinson et al. (*29*)
117.091	$C_6H_{12}O_2H^+$	5.1	Picquet et al. (*133*)
117.127	$C_7H_{16}OH^+$	13.6	Wang et al. (*94*)
117.070	$C_9H_8H^+$	78.0	Atkinson et al. (*29*)
119.070	$C_5H_{10}O_3H^+$	8.8	Wang et al. (*94*)
119.107	$C_6H_{14}O_2H^+$	23.1	Wang et al. (*94*)
119.086	$C_9H_{10}H^+$	50.4	Atkinson et al. (*29*)
120.081	$C_8H_9NH^+$	0.5	Koss et al. (*130*)
121.101	$C_9H_{12}H^+$	22.0	Wang et al. (*94*)
127.112	$C_8H_{14}OH^+$	98.8	Pfannerstill et al. (*131*)
133.086	$C_6H_{12}O_3H^+$	9.9	Wang et al. (*94*)
133.122	$C_7H_{16}O_2H^+$	29.3	Wang et al. (*94*)
133.101	$C_{10}H_{12}H^+$	33.0	NIST database (*129*)
135.117	$C_{10}H_{14}H^+$	9.5	Koss et al. (*130*)
137.132	$C_{10}H_{16}H^+$	162.8	Koss et al. (*130*)
141.127	$C_9H_{16}OH^+$	43.7	Pfannerstill et al. (*131*)
143.143	$C_9H_{18}OH^+$	24.1	Pfannerstill et al. (*131*)
147.102	$C_7H_{14}O_3H^+$	22.9	Baxley et al. (*134*)

m/z	Ion formula	OH rate constant (10^{-12} cm^3 molecule^{-1} s^{-1})	References
147.138	$C_8H_{18}O_2H^+$	273.1	Wang et al. (*94*)
147.117	$C_{11}H_{14}H^+$	58.0	Pfannerstill et al. (*131*)
149.132	$C_{11}H_{16}H^+$	50.0	Koss et al. (*130*)
153.127	$C_{10}H_{16}OH^+$	4.3	Wu et al. (*55*)
157.159	$C_{10}H_{20}OH^+$	13.0	Atkinson et al. (*29*)
205.195	$C_{15}H_{24}H^+$	300.0	Gilman et al. (*128*)
223.064	$C_6H_{18}O_3Si_3H^+$	0.9	Bernard et al. (*135*)
297.082	$C_8H_{24}O_4Si_4H^+$	1.2	Bernard et al. (*135*)
371.101	$C_{10}H_{30}O_5Si_5H^+$	2.9	Bernard et al. (*135*)

Table S4. Top five species for each PMF-resolved factor, including their fractional contributions and OH reaction rate constants (k_{OH}). The sources of k_{OH} values are provided in Table S3.

Factor	Ions	Compounds	Fraction (%)	OH rate constant (10^{-12} cm^3 $molecule^{-1}$ s^{-1})
Biogenic emissions	m69 $C_5H_8H^+$	Isoprene	60.4	100.0
	m71 $C_4H_6OH^+$	MVK+MACR	9.4	24.8
	m47 $C_2H_6OH^+$	Ethanol	4.3	3.2
	m61 $C_2H_4O2H^+$	Acetic acid	4.0	3.7
	m45 $C_2H_4OH^+$	Acetaldehyde	3.1	15.0
Vehicle-related	m57 $C_4H_8H^+$	Butenes	23.8	31.0
	m33 CH_4OH^+	Methanol	21.2	0.8
	m47 $C_2H_6OH^+$	Ethanol	8.8	3.2
	m71 $C_5H_{10}H^+$	Pentenes	6.5	31.4
	m93 $C_7H_8H^+$	Toluene	5.8	5.6
Cooking and household sources	m47 $C_2H_6OH^+$	Ethanol	43.4	3.2
	m33 CH_4OH^+	Methanol	13.7	0.8
	m61 $C_2H_4O_2H^+$	Acetic acid	9.1	3.7
	m45 $C_2H_4OH^+$	Acetaldehyde	4.3	15.0
	m59 $C_3H_6OH^+$	Acetone	3.6	0.2
VCP-related: OVOCs-rich	m47 $C_2H_6OH^+$	Ethanol	52.2	3.2
	m33 CH_4OH^+	Methanol	18.3	0.8
	m31 CH_2OH^+	Formaldehyde	11.3	9.4
	m61 $C_2H_4O_2H^+$	Acetic acid	4.1	3.7
	m45 $C_2H_4OH^+$	Acetaldehyde	2.9	15.0
VCP-related: aromatics-rich	m33 CH_4OH^+	Methanol	33.9	0.8
	m61 $C_2H_4O_2H^+$	Acetic acid	20.1	3.7
	m107 $C_8H_{10}H^+$	C8 aromatics	10.5	13.2
	m47 $C_2H_6OH^+$	Ethanol	7.6	3.2
	m93 $C_7H_8H^+$	Toluene	4.1	5.6

Table S5. Latitude, vegetation cover rate, NDVI, average emission potential, standardized isoprene flux, and the fraction of isoprene emitting trees in surveyed cities. The fraction of isoprene emitting trees is defined as the percentage of the urban tree population with an emission potential of ≥10 μg (g DW)$^{-1}$ h^{-1}.

Type	Site/City	Latitude	Vegetation cover rate (%)	NDVI	Average emission potential (μg (g DW)$^{-1}$ h^{-1})	Standardized isoprene flux by γ_v (nmol m^{-2} s^{-1})*	Standardized isoprene flux by NDVI (nmol m^{-2} s^{-1})*	Fraction of isoprene emitting trees (%)
Measured Sites	Beijing, China	39°58′ 33′′ N	23%	0.37	18.9	6.13		34.7
	Houston, US	29°47′ 22′′ N	24%	0.36	12.2	2.2		27.0
	London, UK	51°30′ 42′′ N	11%	0.13	9.9	1.2		30.2
	Innsbruck, Austria	47°15′ 51′′ N	10%	0.34	8.0	0.67		16.4
	Helsinki, Finland	60°12′ N	47%	0.45	3.3	1.8		6.2
Cities	Harbin, China	45° N	30%	0.47	28.7	8.93	10.28	48.5
	Changchun, China	44° N	37%	0.43	19.9	7.73	6.56	30.6
	Chengdu, China	30° N	38%	0.39	15.1	6.01	4.56	27.9
	Nanjing, China	32° N	40%	0.47	12.3	5.15	4.39	39.6
	Guangzhou, China	23° N	37%	0.41	11	4.26	3.42	35.4
	Shanghai, China	31° N	36%	0.44	8.6	3.24	2.93	18.9

Type	Site/City	Latitude	Vegetation cover rate (%)	NDVI	Average emission potential (μg (g DW)$^{-1}$ h^{-1})	Standardized isoprene flux by γ_v (nmol m^{-2} s^{-1})*	Standardized isoprene flux by NDVI (nmol m^{-2} s^{-1})*	Fraction of isoprene emitting trees (%)
	Seoul, South Korea	37° N	28%	0.39	19.3	5.66	5.86	44.9
	Singapore	1° N	50%	0.41	11	5.76	3.48	35.7
	Muscat, Oman	24° N	12%	0.12	6.6	0.83	0.61	25.2
	Atlanta, US	33° N	68%	0.66	12.1	8.62	6.08	24.0
	New York City, US	41° N	34%	0.39	12.0	4.31	3.61	31.8
	Los Angeles, US	34° N	36%	0.30	10.7	4.03	2.45	34.1
	Paris, France	42° N	23%	0.34	11.3	2.72	3.38	33.6
	Berlin, Germany	52° N	30%	0.53	11.5	3.61	4.70	24.6
	Moscow, Russia	55° N	42%	0.52	7.7	3.39	3.04	12.5
	Sydney, Australia	33° S	32%	0.34	19.9	6.67	5.25	65.9
	Melbourne, Australia	37° S	42%	0.41	15.8	6.95	4.94	55.7
	Lagos, Nigeria	6° N	32%	0.34	9.2	3.11	2.39	34.6
	Rio de Janeiro, Brazil	22° S	44%	0.38	5.4	2.48	2.32	6.0

* For cities without direct measurements, two estimates of standardized isoprene flux are provided, calculated separately using vegetation cover fraction and NDVI, based on the city-level average emission potential.

Table S6. Urban tree inventory and built-up area characteristics across different cities for predicting isoprene emission potential. The total urban built-up area represents the area shown in the NDVI maps in Fig. S21 for each surveyed city. Notably, the data for Paris, Lagos, and Sydney represent the area and inventory of Paris city, Lagos metropolis, and the City of Sydney, respectively, while other cities follow the standard urban administrative boundaries. The urban tree inventory is categorized into two types: urban street trees (S) and urban park trees (P), where "S+P" indicates that both categories are included in the city's inventory used for statistics.

City	Area of urban built-up region (km^2)	Total number of tree species used for statistics	Type of urban tree inventory
Harbin, China	771	20	S+P
Changchun, China	805	26	S+P
Chengdu, China	1587	22	S+P
Nanjing, China	1638	24	S+P
Guangzhou, China	2875	29	S+P
Shanghai, China	4054	12	S+P
Seoul, South Korea	483	20	P
Singapore	608	54	S+P
Muscat, Oman	110	31	P
Atlanta, US	896	50	S
New York City, US	734	70	S
Los Angeles, US	3098	104	S+P
Paris, France	104	100	S+P
Berlin, Germany	552	100	S+P
Moscow, Russia	995	15	S+P
Sydney, Australia	25	100	S+P
Melbourne, Australia	1818	100	S+P
Lagos, Nigeria	163	46	S
Rio de Janeiro, Brazil	565	60	S

Dataset S1. (separate file). Tree inventories of surveyed cities, the corresponding emission potential of tree species, and key tree species for controlling isoprene emissions.

REFERENCES

1. W. Huang, H. Xu, J. Wu, M. Ren, Y. Ke, J. Qiao, Toward cleaner air and better health: Current state, challenges, and priorities. *Science* **385**, 386–390 (2024).

2. J. Vazquez Santiago, H. Hata, E. J. Martinez-Noriega, K. Inoue, Ozone trends and their sensitivity in global megacities under the warming climate. *Nat. Commun.* **15**, 10236 (2024).

3. X. Zhou, M. Li, X. Huang, T. Liu, H. Zhang, X. Qi, Z. Wang, Y. Qin, G. Geng, J. Wang, X. Chi, A. Ding, Urban meteorology–chemistry coupling in compound heat–ozone extremes. *Nat. Cities* **2**, 847–856 (2025).

4. Z. L. Fleming, R. M. Doherty, E. Von Schneidemesser, C. S. Malley, O. R. Cooper, J. P. Pinto, A. Colette, X. Xu, D. Simpson, M. G. Schultz, A. S. Lefohn, S. Hamad, R. Moolla, S. Solberg, Z. Feng, Tropospheric Ozone Assessment Report: Present-day ozone distribution and trends relevant to human health. *Elem. Sci. Anth.* **6**, 12 (2018).

5. X. Lu, L. Zhang, X. Wang, M. Gao, K. Li, Y. Zhang, X. Yue, Y. Zhang, Rapid increases in warm-season surface ozone and resulting health impact in China since 2013. *Environ. Sci. Technol. Lett.* **7**, 240–247 (2020).

6. D. Jacob, *Introduction to Atmospheric Chemistry* (Princeton Univ. Press, 1999).

7. J. H. Seinfeld, S. N. Pandis, *Atmospheric Chemistry and Physics: From Air Pollution to Climate Change* (Wiley, ed. 3, 2016).

8. U. Im, R. Bianconi, E. Solazzo, I. Kioutsioukis, A. Badia, A. Balzarini, R. Baró, R. Bellasio, D. Brunner, C. Chemel, G. Curci, J. Flemming, R. Forkel, L. Giordano, P. Jiménez-Guerrero, M. Hirtl, A. Hodzic, L. Honzak, O. Jorba, C. Knote, J. J. P. Kuenen, P. A. Makar, A. Manders-Groot, L. Neal, J. L. Pérez, G. Pirovano, G. Pouliot, R. San Jose, N. Savage, W. Schroder, R. S. Sokhi, D. Syrakov, A. Torian, P. Tuccella, J. Werhahn, R. Wolke, K. Yahya, R. Zabkar, Y. Zhang, J. Zhang, C. Hogrefe, S. Galmarini, Evaluation of operational on-line-coupled regional air quality models over Europe and North America in the context of AQMEII phase 2. Part I: Ozone. *Atmos. Environ.* **115**, 404–420 (2015).

9. K. W. Appel, A. B. Gilliland, G. Sarwar, R. C. Gilliam, Evaluation of the Community Multiscale Air Quality (CMAQ) model version 4.5: Sensitivities impacting model performance. *Atmos. Environ.* **41**, 9603–9615 (2007).

10. J. Yang, Y. Zhao, Performance and application of air quality models on ozone simulation in China—A review. *Atmos. Environ.* **293**, 119446 (2023).

11. A. C. Lewis, N. Carslaw, P. J. Marriott, R. M. Kinghorn, P. Morrison, A. L. Lee, K. D. Bartle, M. J. Pilling, A larger pool of ozone-forming carbon compounds in urban atmospheres. *Nature* **405**, 778–781 (2000).

12. C. L. Heald, J. H. Kroll, The fuel of atmospheric chemistry: Toward a complete description of reactive organic carbon. *Sci. Adv.* **6**, eaay8967 (2020).

13. B. C. McDonald, J. A. de Gouw, J. B. Gilman, S. H. Jathar, A. Akherati, C. D. Cappa, J. L. Jimenez, J. Lee-Taylor, P. L. Hayes, S. A. McKeen, Y. Y. Cui, S.-W. Kim, D. R. Gentner, G. Isaacman-VanWertz, A. H. Goldstein, R. A. Harley, G. J. Frost, J. M. Roberts, T. B. Ryerson, M. Trainer, Volatile chemical products emerging as largest petrochemical source of urban organic emissions. *Science* **359**, 760–764 (2018).

14. A. C. Lewis, The changing face of urban air pollution. *Science* **359**, 744–745 (2018).

15. A. C. Lewis, J. R. Hopkins, D. C. Carslaw, J. F. Hamilton, B. S. Nelson, G. Stewart, J. Dernie, N. Passant, T. Murrells, An increasing role for solvent emissions and implications for future measurements of volatile organic compounds. *Philos. Trans. Lond. Ser. A Math. Phys. Eng. Sci.* **378**, 20190328 (2020).

16. A. B. Guenther, X. Jiang, C. L. Heald, T. Sakulyanontvittaya, T. Duhl, L. K. Emmons, X. Wang, The Model of Emissions of Gases and Aerosols from Nature version 2.1 (MEGAN2.1): An extended and updated framework for modeling biogenic emissions. *Geosci. Model Dev.* **5**, 1471–1492 (2012).

17. W. L. Chameides, R. W. Lindsay, J. Richardson, C. S. Kiang, The role of biogenic hydrocarbons in urban photochemical smog: Atlanta as a case study. *Science* **241**, 1473–1475 (1988).

18. M. Trainer, E. J. Williams, D. D. Parrish, M. P. Buhr, E. J. Allwine, H. H. Westberg, F. C. Fehsenfeld, S. C. Liu, Models and observations of the impact of natural hydrocarbons on rural ozone. *Nature* **329**, 705–707 (1987).

19. S. Gu, A. Guenther, C. Faiola, Effects of anthropogenic and biogenic volatile organic compounds on Los Angeles air quality. *Environ. Sci. Technol.* **55**, 12191–12201 (2021).

20. H. L. Schlaerth, S. J. Silva, Y. Li, Characterizing ozone sensitivity to urban greening in Los Angeles under current day and future anthropogenic emissions scenarios. *J. Geophys. Res. Atmos.* **128**, e2023JD039199 (2023).

21. Y. Gao, M. Ma, F. Yan, H. Su, S. Wang, H. Liao, B. Zhao, X. Wang, Y. Sun, J. R. Hopkins, Q. Chen, P. Fu, A. C. Lewis, Q. Qiu, X. Yao, H. Gao, Impacts of biogenic emissions from urban landscapes on summer ozone and secondary organic aerosol formation in megacities. *Sci. Total Environ.* **814**, 152654 (2022).

22. M. Ma, Y. Gao, A. Ding, H. Su, H. Liao, S. Wang, X. Wang, B. Zhao, S. Zhang, P. Fu, A. B. Guenther, M. Wang, S. Li, B. Chu, X. Yao, H. Gao, Development and assessment of a high-resolution biogenic emission inventory from urban green spaces in China. *Environ. Sci. Technol.* **56**, 175–184 (2022).

23. D. Wei, C. Cao, A. Karambelas, J. Mak, A. Reinmann, R. Commane, High-resolution modeling of summertime biogenic isoprene emissions in New York City. *Environ. Sci. Technol.* **58**, 13783–13794 (2024).

24. E. Y. Pfannerstill, C. Arata, Q. Zhu, B. C. Schulze, R. Ward, R. Woods, C. Harkins, R. H. Schwantes, J. H. Seinfeld, A. Bucholtz, R. C. Cohen, A. H. Goldstein, Temperature-dependent emissions dominate aerosol and ozone formation in Los Angeles. *Science* **384**, 1324–1329 (2024).

25. J.-H. Park, A. H. Goldstein, J. Timkovsky, S. Fares, R. Weber, J. Karlik, R. Holzinger, Active atmosphere-ecosystem exchange of the vast majority of detected volatile organic compounds. *Science* **341**, 643–647 (2013).

26. D. B. Millet, H. D. Alwe, X. Chen, M. J. Deventer, T. J. Griffis, R. Holzinger, S. B. Bertman, P. S. Rickly, P. S. Stevens, T. Léonardis, N. Locoge, S. Dusanter, G. S. Tyndall, S. L. Alvarez, M. H. Erickson, J. H. Flynn, Bidirectional ecosystem–atmosphere fluxes of volatile organic compounds across the mass spectrum: How many matter? *ACS Earth Space Chem.* **2**, 764–777 (2018).

27. X. He, B. Yuan, Y. Huangfu, S. Wang, Y. Chen, X. Zhang, Y. Sun, T. Karl, M. Shao, Comprehensive characterization and source analysis of VOC fluxes in a Chinese megacity. *Environ. Sci. Technol.* **60**, 6477–6488 (2026).

28. B. Yuan, A. R. Koss, C. Warneke, M. Coggon, K. Sekimoto, J. A. de Gouw, Proton-transfer-reaction mass spectrometry: Applications in atmospheric sciences. *Chem. Rev.* **117**, 13187–13229 (2017).

29. R. Atkinson, J. Arey, Atmospheric degradation of volatile organic compounds. *Chem. Rev.* **103**, 4605–4638 (2003).

30. E. Y. Pfannerstill, C. Arata, Q. Zhu, B. C. Schulze, R. Woods, C. Harkins, R. H. Schwantes, B. C. McDonald, J. H. Seinfeld, A. Bucholtz, R. C. Cohen, A. H. Goldstein, Comparison between spatially resolved airborne flux measurements and emission inventories of volatile organic compounds in Los Angeles. *Environ. Sci. Technol.* **57**, 15533–15545 (2023).

31. M. M. Coggon, G. I. Gkatzelis, B. C. McDonald, J. B. Gilman, R. H. Schwantes, N. Abuhassan, K. C. Aikin, M. F. Arend, T. A. Berkoff, S. S. Brown, T. L. Campos, R. R. Dickerson, G. Gronoff, J. F. Hurley, G. Isaacman-VanWertz, A. R. Koss, M. Li, S. A. McKeen, F. Moshary, J. Peischl, V. Pospisilova, X. Ren, A. Wilson, Y. Wu, M. Trainer, C. Warneke, Volatile chemical product emissions enhance ozone and modulate urban chemistry. *Proc. Natl. Acad. Sci. U.S.A.* **118**, e2026653118 (2021).

32. A. B. Guenther, P. R. Zimmerman, P. C. Harley, R. K. Monson, R. Fall, Isoprene and monoterpene emission rate variability: Model evaluations and sensitivity analyses. *J. Geophys. Res. Atmos.* **98**, 12609–12617 (1993).

33. S. E. Pusede, A. L. Steiner, R. C. Cohen, Temperature and recent trends in the chemistry of continental surface ozone. *Chem. Rev.* **115**, 3898–3918 (2015).

34. D. J. Rasmussen, J. Hu, A. Mahmud, M. J. Kleeman, The ozone–climate penalty: Past, present, and future. *Environ. Sci. Technol.* **47**, 14258–14266 (2013).

35. P. Wang, Y. Yang, H. Li, L. Chen, R. Dang, D. Xue, B. Li, J. Tang, L. R. Leung, H. Liao, North China Plain as a hot spot of ozone pollution exacerbated by extreme high temperatures. *Atmos. Chem. Phys.* **22**, 4705–4719 (2022).

36. P. Yang, G. Ren, W. Liu, Spatial and temporal characteristics of beijing urban heat island intensity. *J. Appl. Meteorol. Climatol.* **52**, 1803–1816 (2013).

37. K. C. Wells, D. B. Millet, V. H. Payne, M. J. Deventer, K. H. Bates, J. A. De Gouw, M. Graus, C. Warneke, A. Wisthaler, J. D. Fuentes, Satellite isoprene retrievals constrain emissions and atmospheric oxidation. *Nature* **585**, 225–233 (2020).

38. W. J. F. Acton, Z. Huang, B. Davison, W. S. Drysdale, P. Fu, M. Hollaway, B. Langford, J. Lee, Y. Liu, S. Metzger, N. Mullinger, E. Nemitz, C. E. Reeves, F. A. Squires, A. R. Vaughan, X. Wang, Z. Wang, O. Wild, Q. Zhang, Y. Zhang, C. N. Hewitt, Surface–atmosphere fluxes of volatile organic compounds in Beijing. *Atmos. Chem. Phys.* **20**, 15101–15125 (2020).

39. W. J. F. Acton, S. Schallhart, B. Langford, A. Valach, P. Rantala, S. Fares, G. Carriero, R. Tillmann, S. J. Tomlinson, U. Dragosits, D. Gianelle, C. N. Hewitt, E. Nemitz, Canopy-scale flux measurements and bottom-up emission estimates of volatile organic compounds from a mixed oak and hornbeam forest in northern Italy. *Atmos. Chem. Phys.* **16**, 7149–7170 (2016).

40. Beijing Municipal Foresty and Parks Bureau, "Beijing's Forest City Development Plan (2018-2035)" (Beijing Municipal Foresty and Parks Bureau, 2020); https://yllhj.beijing.gov.cn/zwgk/ghxx/gh/202004/t20200423_1881046.shtml.

41. A. Guenther, J. Greenberg, P. Harley, D. Helmig, L. Klinger, L. Vierling, P. Zimmerman, C. Geron, Leaf, branch, stand and landscape scale measurements of volatile organic compound fluxes from U.S. woodlands. *Tree Physiol.* **16**, 17–24 (1996).

42. A. Ghirardo, J. Xie, X. Zheng, Y. Wang, R. Grote, K. Block, J. Wildt, T. Mentel, A. Kiendler-Scharr, M. Hallquist, K. Butterbach-Bahl, J.-P. Schnitzler, Urban stress-induced biogenic VOC emissions and SOA-forming potentials in Beijing. *Atmos. Chem. Phys.* **16**, 2901–2920 (2016).

43. P. Yan, J. Yang, Species diversity of urban forests in China. *Urban For. Urban Green.* **28**, 160–166 (2017).

44. C. Geron, P. Harley, A. Guenther, Isoprene emission capacity for US tree species. *Atmos. Environ.* **35**, 3341–3352 (2001).

45. Intergovernmental Panel On Climate Change (IPCC), *Climate Change 2022: Impacts, Adaptation, and Vulnerability. Contribution of Working Group II to the Sixth Assessment Report of the Intergovernmental Panel on Climate Change* (Cambridge Univ. Press, ed. 1, 2023).

46. Y. Li, S. Schubert, J. P. Kropp, D. Rybski, On the influence of density and morphology on the Urban Heat Island intensity. *Nat. Commun.* **11**, 2647 (2020).

47. L. Sun, J. Chen, Q. Li, D. Huang, Dramatic uneven urbanization of large cities throughout the world in recent decades. *Nat. Commun.* **11**, 5366 (2020).

48. J. Byron, J. Kreuzwieser, G. Purser, J. Van Haren, S. N. Ladd, L. K. Meredith, C. Werner, J. Williams, Chiral monoterpenes reveal forest emission mechanisms and drought responses. *Nature* **609**, 307–312 (2022).

49. R. Seco, T. Karl, A. Guenther, K. P. Hosman, S. G. Pallardy, L. Gu, C. Geron, P. Harley, S. Kim, Ecosystem-scale volatile organic compound fluxes during an extreme drought in a broadleaf temperate forest of the Missouri Ozarks (central USA). *Glob. Change Biol.* **21**, 3657–3674 (2015).

50. Z. Feng, P. Büker, H. Pleijel, L. Emberson, P. E. Karlsson, J. Uddling, A unifying explanation for variation in ozone sensitivity among woody plants. *Glob. Change Biol.* **24**, 78–84 (2018).

51. S. Li, X. Yuan, S. Li, Y. Zhou, S. Wang, K. Zhang, E. Agathokleous, J. D. Blande, Z. Feng, Extreme heat event alters BVOC responses to elevated ozone: From physiology to emission patterns. *Environ. Sci. Technol.* **59**, 12132–12144 (2025).

52. S. Wu, B. Chen, C. Webster, B. Xu, P. Gong, Improved human greenspace exposure equality during 21st century urbanization. *Nat. Commun.* **14**, 6460 (2023).

53. The State Council of China, "Guiding Opinions of the General Office of the State Council on Scientific Greening" (2021); https://www.gov.cn/zhengce/zhengceku/2021-06/02/content_5614922.htm.

54. K. Sekimoto, S.-M. Li, B. Yuan, A. Koss, M. Coggon, C. Warneke, J. de Gouw, Calculation of the sensitivity of proton-transfer-reaction mass spectrometry (PTR-MS) for organic trace gases using molecular properties. *Int. J. Mass Spectrom.* **421**, 71–94 (2017).

55. C. Wu, C. Wang, S. Wang, W. Wang, B. Yuan, J. Qi, B. Wang, H. Wang, C. Wang, W. Song, X. Wang, W. Hu, S. Lou, C. Ye, Y. Peng, Z. Wang, Y. Huangfu, Y. Xie, M. Zhu, J. Zheng, X. Wang, B. Jiang, Z. Zhang, M. Shao, Measurement report: Important contributions of oxygenated compounds to emissions and chemistry of volatile organic compounds in urban air. *Atmos. Chem. Phys.* **20**, 14769–14785 (2020).

56. X. He, B. Yuan, C. Wu, S. Wang, C. Wang, Y. Huangfu, J. Qi, N. Ma, W. Xu, M. Wang, W. Chen, H. Su, Y. Cheng, M. Shao, Volatile organic compounds in wintertime North China Plain: Insights from measurements of proton transfer reaction time-of-flight mass spectrometer (PTR-ToF-MS). *J. Environ. Sci.* **114**, 98–114 (2022).

57. M. M. Coggon, C. E. Stockwell, M. S. Claflin, E. Y. Pfannerstill, L. Xu, J. B. Gilman, J. Marcantonio, C. Cao, K. Bates, G. I. Gkatzelis, A. Lamplugh, E. F. Katz, C. Arata, E. C. Apel, R. S. Hornbrook, F. Piel, F. Majluf, D. R. Blake, A. Wisthaler, M. Canagaratna, B. M. Lerner, A. H. Goldstein, J. E. Mak, C. Warneke, Identifying and correcting interferences to

PTR-ToF-MS measurements of isoprene and other urban volatile organic compounds. *Atmos. Meas. Tech.* **17**, 801–825 (2024).

58. M. Striednig, M. Graus, T. D. Märk, T. G. Karl, InnFLUX – an open-source code for conventional and disjunct eddy covariance analysis of trace gas measurements: An urban test case. *Atmos. Meas. Tech.* **13**, 1447–1465 (2020).

59. Y. Huangfu, B. Yuan, X. He, Z. Liu, Y. Zhang, T. Karl, M. Striednig, Y. Ding, X. Chen, H. Li, H. Zheng, M. Chang, X. Wang, M. Shao, Natural gas leakage ratio determined from flux measurements of methane in Urban Beijing. *Environ. Sci. Technol. Lett.* **11**, 1025–1031 (2024).

60. N. Kljun, P. Calanca, M. W. Rotach, H. P. Schmid, A simple two-dimensional parameterisation for Flux Footprint Prediction (FFP). *Geosci. Model Dev.* **8**, 3695–3713 (2015).

61. P. Paatero, U. Tapper, Positive matrix factorization: A non-negative factor model with optimal utilization of error estimates of data values. *Environmetrics* **5**, 111–126 (1994).

62. I. M. Ulbrich, M. R. Canagaratna, Q. Zhang, D. R. Worsnop, J. L. Jimenez, Interpretation of organic components from Positive Matrix Factorization of aerosol mass spectrometric data. *Atmos. Chem. Phys.* **9**, 2891–2918 (2009).

63. M. Wang, M. Shao, W. Chen, B. Yuan, S. Lu, Q. Zhang, L. Zeng, Q. Wang, A temporally and spatially resolved validation of emission inventories by measurements of ambient volatile organic compounds in Beijing, China. *Atmos. Chem. Phys.* **14**, 5871–5891 (2014).

64. B. Yuan, M. Shao, J. de Gouw, D. D. Parrish, S. Lu, M. Wang, L. Zeng, Q. Zhang, Y. Song, J. Zhang, M. Hu, Volatile organic compounds (VOCs) in urban air: How chemistry affects the interpretation of positive matrix factorization (PMF) analysis. *J. Geophys. Res. Atmos.* **117**, D24302 (2012).

65. X. L. Cheng, X. M. Liu, Y. J. Liu, F. Hu, Characteristics of CO_2 concentration and flux in the beijing urban area. *J. Geophys. Res. Atmos.* **123**, 1785–1801 (2018).

66. Z. Mo, M. Shao, W. Wang, Y. Liu, M. Wang, S. Lu, Evaluation of biogenic isoprene emissions and their contribution to ozone formation by ground-based measurements in Beijing, China. *Sci. Total Environ.* **627**, 1485–1494 (2018).

67. G. M. Wolfe, M. R. Marvin, S. J. Roberts, K. R. Travis, J. Liao, The Framework for 0-D Atmospheric Modeling (F0AM) v3.1. *Geosci. Model Dev.* **9**, 3309–3319 (2016).

68. M. Li, Q. Zhang, B. Zheng, D. Tong, Y. Lei, F. Liu, C. Hong, S. Kang, L. Yan, Y. Zhang, Y. Bo, H. Su, Y. Cheng, K. He, Persistent growth of anthropogenic non-methane volatile organic compound (NMVOC) emissions in China during 1990–2017: Drivers, speciation and ozone formation potential. *Atmos. Chem. Phys.* **19**, 8897–8913 (2019).

69. Q. Xie, W. Chen, B. Yuan, Y. Huangfu, X. He, L. Wu, M. Liu, Y. You, M. Shao, X. Wang, Significant but overlooked: The role of anthropogenic monoterpenes in ozone formation in a Chinese megacity. *Environ. Sci. Technol.* **59**, 8085–8098 (2025).

70. W. S. Goliff, W. R. Stockwell, C. V. Lawson, The Regional Atmospheric Chemistry Mechanism, version 2. *Atmos. Environ.* **68**, 174–185 (2013).

71. P. Di Carlo, W. H. Brune, M. Martinez, H. Harder, R. Lesher, X. Ren, T. Thornberry, M. A. Carroll, V. Young, P. B. Shepson, D. Riemer, E. Apel, C. Campbell, Missing OH reactivity in a forest: Evidence for unknown reactive biogenic VOCs. *Science* **304**, 722–725 (2004).

72. A. L. Steiner, A. J. Davis, S. Sillman, R. C. Owen, A. M. Michalak, A. M. Fiore, Observed suppression of ozone formation at extremely high temperatures due to chemical and biophysical feedbacks. *Proc. Natl. Acad. Sci. U.S.A.* **107**, 19685–19690 (2010).

73. A. Guenther, T. Karl, P. Harley, C. Wiedinmyer, P. I. Palmer, C. Geron, Estimates of global terrestrial isoprene emissions using MEGAN (Model of Emissions of Gases and Aerosols from Nature). *Atmos. Chem. Phys.* **6**, 3181–3210 (2006).

74. L. Kaser, A. Peron, M. Graus, M. Striednig, G. Wohlfahrt, S. Juráň, T. Karl, Interannual variability of terpenoid emissions in an alpine city. *Atmos. Chem. Phys.* **22**, 5603–5618 (2022).

75. A. C. Valach, B. Langford, E. Nemitz, A. R. MacKenzie, C. N. Hewitt, Seasonal and diurnal trends in concentrations and fluxes of volatile organic compounds in central London. *Atmos. Chem. Phys.* **15**, 7777–7796 (2015).

76. C. Park, G. W. Schade, I. Boedeker, Characteristics of the flux of isoprene and its oxidation products in an urban area. *J. Geophys. Res. Atmos.* **116**, D21303 (2011).

77. P. Rantala, L. Järvi, R. Taipale, T. K. Laurila, J. Patokoski, M. K. Kajos, M. Kurppa, S. Haapanala, E. Siivola, T. Petäjä, T. M. Ruuskanen, J. Rinne, Anthropogenic and biogenic influence on VOC fluxes at an urban background site in Helsinki, Finland. *Atmos. Chem. Phys.* **16**, 7981–8007 (2016).

78. A. H. Goldstein, M. L. Goulden, J. W. Munger, S. C. Wofsy, C. D. Geron, Seasonal course of isoprene emissions from a midlatitude deciduous forest. *J. Geophys. Res. Atmos.* **103**, 31045–31056 (1998).

79. B. Davison, R. Taipale, B. Langford, P. Misztal, S. Fares, G. Matteucci, F. Loreto, J. N. Cape, J. Rinne, C. N. Hewitt, Concentrations and fluxes of biogenic volatile organic compounds above a Mediterranean macchia ecosystem in western Italy. *Biogeosciences* **6**, 1655–1670 (2009).

80. M. J. Potosnak, L. LeStourgeon, S. G. Pallardy, K. P. Hosman, L. Gu, T. Karl, C. Geron, A. B. Guenther, Observed and modeled ecosystem isoprene fluxes from an oak-dominated temperate forest and the influence of drought stress. *Atmos. Environ.* **84**, 314–322 (2014).

81. E. Gomes Alves, R. Aquino Santana, C. Quaresma Dias-Júnior, S. Botía, T. Taylor, A. M. Yáñez-Serrano, J. Kesselmeier, E. Bourtsoukidis, J. Williams, P. I. Lembo Silveira De Assis, G. Martins, R. De Souza, S. Duvoisin Júnior, A. Guenther, D. Gu, A. Tsokankunku, M. Sörgel, B. Nelson, D. Pinto, S. Komiya, D. Martins Rosa, B. Weber, C. Barbosa, M. Robin, K. J. Feeley, A. Duque, V. Londoño Lemos, M. P. Contreras, A. Idarraga, N. López, C. Husby, B. Jestrow, I. M. Cely Toro, Intra- and interannual changes in isoprene emission from central Amazonia. *Atmos. Chem. Phys.* **23**, 8149–8168 (2023).

82. T. Karl, A. Guenther, A. Turnipseed, G. Tyndall, P. Artaxo, S. Martin, Rapid formation of isoprene photo-oxidation products observed in Amazonia. *Atmos. Chem. Phys.* **9**, 7753–7767 (2009).

83. X. Lee, W. Massman, B. Law, *Handbook of Micrometeorology: A Guide for Surface Flux Measurement and Analysis* (Springer Science & Business Media, 2004), vol. 29.

84. T. Foken, *Micrometeorology* (Springer, 2017).

85. W. Lindinger, A. Hansel, A. Jordan, On-line monitoring of volatile organic compounds at pptv levels by means of proton-transfer-reaction mass spectrometry (PTR-MS) medical applications, food control and environmental research. *Int. J. Mass Spectrom. Ion Process.* **173**, 191–241 (1998).

86. M. Gueneron, M. H. Erickson, G. S. VanderSchelden, B. T. Jobson, PTR-MS fragmentation patterns of gasoline hydrocarbons. *Int. J. Mass Spectrom.* **379**, 97–109 (2015).

87. S. Kim, T. Karl, D. Helmig, R. Daly, R. Rasmussen, A. Guenther, Measurement of atmospheric sesquiterpenes by proton transfer reaction-mass spectrometry (PTR-MS). *Atmos. Meas. Tech.* **2**, 99–112 (2009).

88. T. Berndt, N. Hyttinen, H. Herrmann, A. Hansel, First oxidation products from the reaction of hydroxyl radicals with isoprene for pristine environmental conditions. *Commun. Chem.* **2**, 21 (2019).

89. F. Klein, S. M. Platt, N. J. Farren, A. Detournay, E. A. Bruns, C. Bozzetti, K. R. Daellenbach, D. Kilic, N. K. Kumar, S. M. Pieber, J. G. Slowik, B. Temime-Roussel, N. Marchand, J. F. Hamilton, U. Baltensperger, A. S. H. Prévôt, I. El Haddad, Characterization of gas-phase organics using proton transfer reaction time-of-flight mass spectrometry: Cooking emissions. *Environ. Sci. Technol.* **50**, 1243–1250 (2016).

90. C. E. Stockwell, P. R. Veres, J. Williams, R. J. Yokelson, Characterization of biomass burning emissions from cooking fires, peat, crop residue, and other fuels with high-resolution proton-transfer-reaction time-of-flight mass spectrometry. *Atmos. Chem. Phys.* **15**, 845–865 (2015).

91. S. Wang, B. Yuan, C. Wu, C. Wang, T. Li, X. He, Y. Huangfu, J. Qi, X.-B. Li, Q. Sha, M. Zhu, S. Lou, H. Wang, T. Karl, M. Graus, Z. Yuan, M. Shao, Oxygenated volatile organic compounds (VOCs) as significant but varied contributors to VOC emissions from vehicles. *Atmos. Chem. Phys.* **22**, 9703–9720 (2022).

92. B. T. Jobson, M. L. Alexander, G. D. Maupin, G. G. Muntean, On-line analysis of organic compounds in diesel exhaust using a proton transfer reaction mass spectrometer (PTR-MS). *Int. J. Mass Spectrom.* **245**, 78–89 (2005).

93. K. M. Seltzer, E. Pennington, V. Rao, B. N. Murphy, M. Strum, K. K. Isaacs, H. O. T. Pye, Reactive organic carbon emissions from volatile chemical products. *Atmos. Chem. Phys.* **21**, 5079–5100 (2021).

94. S. Wang, B. Yuan, X. He, R. Cui, X. Song, Y. Chen, C. Wu, C. Wang, Y. Huangfu, X. Li, B. Wang, M. Shao, Emission characteristics of reactive organic gases (ROGs) from industrial volatile chemical products (VCPs) in the Pearl River Delta (PRD), China. *Atmos. Chem. Phys.* **24**, 7101–7121 (2024).

95. S. Zhu, C. Yan, J. Zheng, C. Chen, H. Ning, D. Yang, M. Wang, Y. Ma, J. Zhan, C. Hua, R. Yin, Y. Li, Y. Liu, J. Jiang, L. Yao, L. Wang, M. Kulmala, D. R. Worsnop, Observation and source apportionment of atmospheric alkaline gases in Urban Beijing. *Environ. Sci. Technol.* **56**, 17545–17555 (2022).

96. Z. Mo, R. Cui, B. Yuan, H. Cai, B. C. McDonald, M. Li, J. Zheng, M. Shao, A mass-balance-based emission inventory of non-methane volatile organic compounds (NMVOCs) for solvent use in China. *Atmos. Chem. Phys.* **21**, 13655–13666 (2021).

97. S. Liu, B. Barletta, R. S. Hornbrook, A. Fried, J. Peischl, S. Meinardi, M. Coggon, A. Lamplugh, J. B. Gilman, G. I. Gkatzelis, C. Warneke, E. C. Apel, A. J. Hills, I. Bourgeois, J. Walega, P. Weibring, D. Richter, T. Kuwayama, M. FitzGibbon, D. Blake, Composition and reactivity of volatile organic compounds in the South Coast Air Basin and San Joaquin Valley of California. *Atmos. Chem. Phys.* **22**, 10937–10954 (2022).

98. R. F. Hansen, S. M. Griffith, S. Dusanter, J. B. Gilman, M. Graus, W. C. Kuster, P. R. Veres, J. A. de Gouw, C. Warneke, R. A. Washenfelder, C. J. Young, S. S. Brown, S. L. Alvarez, J. H. Flynn, N. E. Grossberg, B. Lefer, B. Rappenglueck, P. S. Stevens, Measurements of total OH reactivity during CalNex-LA. *J. Geophys. Res. Atmos.* **126**, e2020JD032988 (2021).

99. M. P. Vermeuel, D. B. Millet, D. K. Farmer, M. A. Pothier, M. F. Link, M. Riches, S. Williams, L. A. Garofalo, Closing the reactive carbon flux budget: Observations from dual mass spectrometers over a coniferous forest. *J. Geophys. Res. Atmos.* **128**, e2023JD038753 (2023).

100. F. Feng, X. Yang, B. Jia, X. Li, X. Li, C. Xu, K. Wang, Variability of urban fractional vegetation cover and its driving factors in 328 cities in China. *Sci. China Earth Sci.* **67**, 466–482 (2024).

101. R. Zhang, Y. Huangfu, B. Yuan, X. Zhang, X. He, T. Karl, Y. Ding, H. Li, K.-E. Min, Y. Xiong, X.-B. Li, Z. Peng, J. Chi, M. Chang, X. Wang, M. Shao, Unexpected V-shaped temperature dependence of methane fluxes informing its sources in a megacity of China. *Sci. Bull.*, 10.1016/j.scib.2026.03.005 (2026).

102. A. T. Archibald, S. T. Turnock, P. T. Griffiths, T. Cox, R. G. Derwent, C. Knote, M. Shin, On the changes in surface ozone over the twenty-first century: Sensitivity to changes in surface temperature and chemical mechanisms. *Philos. Trans. Lond. Ser. A Math. Phys. Eng. Sci.* **378**, 20190329 (2020).

103. T. Karl, M. Striednig, M. Graus, A. Hammerle, G. Wohlfahrt, Urban flux measurements reveal a large pool of oxygenated volatile organic compound emissions. *Proc. Natl. Acad. Sci. U.S.A.* **115**, 1186–1191 (2018).

104. T. Karl, C. Lamprecht, M. Graus, A. Cede, M. Tiefengraber, J. Vila-Guerau de Arellano, D. Gurarie, D. Lenschow, High urban NOx triggers a substantial chemical downward flux of ozone. *Sci. Adv.* **9**, eadd2365 (2023).

105. T.-M. Fu, H. Tian, Climate change penalty to ozone air quality: Review of current understandings and knowledge gaps. *Curr. Pollution Rep.* **5**, 159–171 (2019).

106. K. D. Lu, F. Rohrer, F. Holland, H. Fuchs, B. Bohn, T. Brauers, C. C. Chang, R. Häseler, M. Hu, K. Kita, Y. Kondo, X. Li, S. R. Lou, S. Nehr, M. Shao, L. M. Zeng, A. Wahner, Y. H. Zhang, A. Hofzumahaus, Observation and modelling of OH and HO2 concentrations in the Pearl River Delta 2006: A missing OH source in a VOC rich atmosphere. *Atmos. Chem. Phys.* **12**, 1541–1569 (2012).

107. C. A. Cardelino, W. L. Chameides, An observation-based model for analyzing ozone precursor relationships in the urban atmosphere. *J. Air Waste Manag. Assoc.* **45**, 161–180 (1995).

108. N. S. Panji, D. F. McGlynn, L. E. R. Barry, T. M. Scanlon, M. T. Lerdau, S. E. Pusede, G. Isaacman-VanWertz, Constraining light dependency in modeled emissions through comparison to observed biogenic volatile organic compound (BVOC) concentrations in a southeastern US forest. *Atmos. Chem. Phys.* **24**, 12495–12507 (2024).

109. Y. Zhang, H. Ran, A. Guenther, Q. Zhang, C. George, W. Mellouki, G. Sheng, P. Peng, X. Wang, Improved modelling of biogenic emissions in human-disturbed forest edges and urban areas. *Nat. Commun.* **16**, 8064 (2025).

110. Y. Ren, Z. Qu, Y. Du, R. Xu, D. Ma, G. Yang, Y. Shi, X. Fan, A. Tani, P. Guo, Y. Ge, J. Chang, Air quality and health effects of biogenic volatile organic compounds emissions from urban green spaces and the mitigation strategies. *Environ. Pollut.* **230**, 849–861 (2017).

111. J. Kesselmeier, M. Staudt, Biogenic volatile organic compounds (VOC): An overview on emission, physiology and ecology. *J. Atmos. Chem.* **33**, 23–88 (1999).

112. E. Von Schneidemesser, P. S. Monks, V. Gros, J. Gauduin, O. Sanchez, How important is biogenic isoprene in an urban environment? A study in London and Paris. *Geophys. Res. Lett.* **38**, L19804 (2011).

113. T. D. Sharkey, A. E. Wiberley, A. R. Donohue, Isoprene emission from plants: Why and how. *Ann. Bot.* **101**, 5–18 (2007).

114. T. Karl, E. Apel, A. Hodzic, D. D. Riemer, D. R. Blake, C. Wiedinmyer, Emissions of volatile organic compounds inferred from airborne flux measurements over a megacity. *Atmos. Chem. Phys.* **9**, 271–285 (2009).

115. X. Lun, Y. Lin, F. Chai, C. Fan, H. Li, J. Liu, Reviews of emission of biogenic volatile organic compounds (BVOCs) in Asia. *J. Environ. Sci.* **95**, 266–277 (2020).

116. L. F. Klinger, Q.-J. Li, A. B. Guenther, J. P. Greenberg, B. Baker, J.-H. Bai, Assessment of volatile organic compound emissions from ecosystems of China. *J. Geophys. Res. Atmos.* **107**, 4603 (2002).

117. X. Yuan, Y. Xu, V. Calatayud, Z. Li, Z. Feng, F. Loreto, Emissions of isoprene and monoterpenes from urban tree species in China and relationships with their driving factors. *Atmos. Environ.* **314**, 120096 (2023).

118. H. E. Stewart, A highly spatially and temporally resolved inventory for biogenic isoprene and monoterpene emissions: Model description and application to Great Britain. *J. Geophys. Res. Atmos.* **108**, 4644 (2003).

119. R. S. Defries, J. R. G. Townshend, NDVI-derived land cover classifications at a global scale. *Int. J. Remote Sens.* **15**, 3567–3586 (1994).

120. N. Pettorelli, J. O. Vik, A. Mysterud, J.-M. Gaillard, C. J. Tucker, N. Chr, Stenseth, Using the satellite-derived NDVI to assess ecological responses to environmental change. *Trends Ecol. Evol.* **20**, 503–510 (2005).

121. K. Didan, MOD13Q1 MODIS/Terra Vegetation Indices 16-Day L3 Global 250m SIN Grid V006, NASA Land Processes Distributed Active Archive Center (2015); https://doi.org/10.5067/MODIS/MOD13Q1.006.

122. X. Li, P. Gong, Y. Zhou, J. Wang, Y. Bai, B. Chen, T. Hu, Y. Xiao, B. Xu, J. Yang, X. Liu, W. Cai, H. Huang, T. Wu, X. Wang, P. Lin, X. Li, J. Chen, C. He, X. Li, L. Yu, N. Clinton, Z. Zhu, Mapping global urban boundaries from the global artificial impervious area (GAIA) data. *Environ. Res. Lett.* **15**, 094044 (2020).

123. B. Langford, W. Acton, C. Ammann, A. Valach, E. Nemitz, Eddy-covariance data with low signal-to-noise ratio: Time-lag determination, uncertainties and limit of detection. *Atmos. Meas. Tech.* **8**, 4197–4213 (2015).

124. G. A. Norris, R. Vedantham, K. Wade, S. Brown, J. Prouty, C. Foley, "EPA Positive Matrix Factorization (PMF) 3.0 Funda mentals and User Guide" (Environmental Protection Agency, 2008).

125. G. Isaacman-VanWertz, B. Aumont, Impact of organic molecular structure on the estimation of atmospherically relevant physicochemical parameters. *Atmos. Chem. Phys.* **21**, 6541–6563 (2021).

126. S. Yang, B. Yuan, Y. Peng, S. Huang, W. Chen, W. Hu, C. Pei, J. Zhou, D. D. Parrish, W. Wang, X. He, C. Cheng, X.-B. Li, X. Yang, Y. Song, H. Wang, J. Qi, B. Wang, C. Wang, C. Wang, Z. Wang, T. Li, E. Zheng, S. Wang, C. Wu, M. Cai, C. Ye, W. Song, P. Cheng, D. Chen, X. Wang, Z. Zhang, X. Wang, J. Zheng, M. Shao, The formation and mitigation of nitrate pollution: Comparison between urban and suburban environments. *Atmos. Chem. Phys.* **22**, 4539–4556 (2022).

127. C. A. Cantrell, Technical Note: Review of methods for linear least-squares fitting of data and application to atmospheric chemistry problems. *Atmos. Chem. Phys.* **8**, 5477–5487 (2008).

128. J. B. Gilman, B. M. Lerner, W. C. Kuster, P. D. Goldan, C. Warneke, P. R. Veres, J. M. Roberts, J. A. de Gouw, I. R. Burling, R. J. Yokelson, Biomass burning emissions and potential air quality impacts of volatile organic compounds and other trace gases from fuels common in the US. *Atmos. Chem. Phys.* **15**, 13915–13938 (2015).

129. J. Manion, R. Huie, R. Levin, D. Burgess, V. Orkin, W. Tsang, W. McGivern, J. Hudgens, V. Knyazev, D. Atkinson, E. Chai, A. Tereza, C. Lin, T. Allison, W. Mallard, F. Westley, J. Herron, R. Hampson, D. Frizzell, NIST Chemical Kinetics Database, version Standard Reference Database 17, Version 7.1 (Web Version), Release 1.6.8, Data Version 2024 (NIST, 2024).

130. A. R. Koss, K. Sekimoto, J. B. Gilman, V. Selimovic, M. M. Coggon, K. J. Zarzana, B. Yuan, B. M. Lerner, S. S. Brown, J. L. Jimenez, J. Krechmer, J. M. Roberts, C. Warneke, R. J. Yokelson, J. de Gouw, Non-methane organic gas emissions from biomass burning: Identification, quantification, and emission factors from PTR-ToF during the FIREX 2016 laboratory experiment. *Atmos. Chem. Phys.* **18**, 3299–3319 (2018).

131. E. Y. Pfannerstill, N. Wang, A. Edtbauer, E. Bourtsoukidis, J. N. Crowley, D. Dienhart, P. G. Eger, L. Ernle, H. Fischer, B. Hottmann, J.-D. Paris, C. Stönner, I. Tadic, D. Walter, J. Lelieveld, J. Williams, Shipborne measurements of total OH reactivity around the Arabian Peninsula and its role in ozone chemistry. *Atmos. Chem. Phys.* **19**, 11501–11523 (2019).

132. J. A. Liljegren, P. S. Stevens, Measurements of the kinetics of the reaction of OH radicals with 3-methylfuran at low pressure. *Int. J. Chem. Kinet.* **45**, 787–794 (2013).

133. B. Picquet, S. Heroux, A. Chebbi, J.-F. Doussin, R. Durand-Jolibois, A. Monod, H. Loirat, P. Carlier, Kinetics of the reactions of OH radicals with some oxygenated volatile organic compounds under simulated atmospheric conditions. *Int. J. Chem. Kinet.* **30**, 839–847 (1998).

134. J. S. Baxley, M. V. Henley, J. R. Wells, The hydroxyl radical reaction rate constant and products of ethyl 3-ethoxypropionate. *Int. J. Chem. Kinet.* **29**, 637–644 (1997).

135. F. Bernard, D. K. Papanastasiou, V. C. Papadimitriou, J. B. Burkholder, Temperature dependent rate coefficients for the gas-phase reaction of the OH radical with linear (L2 , L3) and cyclic (D3 , D4) permethylsiloxanes. *J. Phys. Chem. A* **122**, 4252–4264 (2018).
